# Plasma proteomic profiling identifies CD33 as a marker of HIV control in natural infection and after therapeutic vaccination

**DOI:** 10.1016/j.ebiom.2023.104732

**Published:** 2023-07-26

**Authors:** Clara Duran-Castells, Anna Prats, Bruna Oriol-Tordera, Anuska Llano, Cristina Galvez, Javier Martinez-Picado, Ester Ballana, Edurne Garcia-Vidal, Bonaventura Clotet, Jose A. Muñoz-Moreno, Thomas Hanke, José Moltó, Beatriz Mothe, Christian Brander, Marta Ruiz-Riol

**Affiliations:** a IrsiCaixa AIDS Research Institute Badalona, Hospital Universitari Germans Trias i Pujol, Badalona, Spain; b Universitat Autònoma de Barcelona, Spain; c Fight Infections Foundation and Infectious Diseases Department, Hospital Universitari Germans Trias i Pujol, Badalona, Spain; d University of Vic-Central University of Catalonia (UVic-UCC), Vic, Spain; e Faculty of Psychology and Education Sciences, Universitat Oberta de Catalunya (UOC), Barcelona, Spain; f Catalan Institution for Research Advanced Studies (ICREA), Barcelona, Spain; g CIBER de Enfermedades Infecciosas (CIBERINFEC), Madrid, Spain; h The Jenner Institute, University of Oxford, Oxford OX3 7DQ, UK; i Joint Research Center for Human Retrovirus Infection, Kumamoto University, Kumamoto, Japan

**Keywords:** Control of HIV-1 infection, Plasma proteomics, Inflammation, Neurological function, Siglec-3/CD33, Kick and kill HIV cure strategy

## Abstract

**Background:**

Biomarkers predicting the outcome of HIV-1 virus control in natural infection and after therapeutic interventions in HIV-1 cure trials remain poorly defined. The BCN02 trial (NCT02616874), combined a T-cell vaccine with romidepsin (RMD), a cancer-drug that was used to promote HIV-1 latency reversal and which has also been shown to have beneficial effects on neurofunction. We conducted longitudinal plasma proteomics analyses in trial participants to define biomarkers associated with virus control during monitored antiretroviral pause (MAP) and to identify novel therapeutic targets that can improve future cure strategies.

**Methods:**

BCN02 was a phase I, open-label, single-arm clinical trial in early-treated, HIV infected individuals. Longitudinal plasma proteomes were analyzed in 11 BCN02 participants, including 8 participants that showed a rapid HIV-1 plasma rebound during a monitored antiretroviral pause (MAP-NC, ‘non-controllers’) and 3 that remained off ART with sustained plasma viremia <2000 copies/ml (MAP-C, ‘controllers’). Inflammatory and neurological proteomes in plasma were evaluated and integration data analysis (viral and neurocognitive parameters) was performed. Validation studies were conducted in a cohort of untreated HIV-1+ individuals (n = 96) and *in vitro* viral replication assays using an anti-CD33 antibody were used for functional validation.

**Findings:**

Inflammatory plasma proteomes in BCN02 participants showed marked longitudinal alterations. Strong proteome differences were also observed between MAP-C and MAP-NC, including in baseline timepoints. CD33/Siglec-3 was the unique plasma marker with the ability to discriminate between MAPC-C and MAP-NC at all study timepoints and showed positive correlations with viral parameters. Analyses in an untreated cohort of PLWH confirmed the positive correlation between viral parameters and CD33 plasma levels, as well as PBMC gene expression. Finally, adding an anti-CD33 antibody to *in vitro* virus cultures significantly reduced HIV-1 replication and proviral levels in T cells and macrophages.

**Interpretation:**

This study indicates that CD33/Siglec-3 may serve as a predictor of HIV-1 control and as potential therapeutic tool to improve future cure strategies.

**Funding:**

Spanish Science and Innovation Ministry (SAF2017-89726-R and PID2020-119710RB-I00), 10.13039/100000002NIH (P01-AI131568), 10.13039/501100000780European Commission (GA101057548) and a 10.13039/501100016387Grifols research agreement.

**Research in context**

**Evidence before this study**

Strategies for an effective HIV cure are complicated by the ability of the virus to establish latent reservoirs in infected cells. The BCN02 clinical study (NCT02616874) used a “kick and kill” strategy, combining the latency reversing agent (RMD) and a therapeutic HIV-1 vaccine with the aim to reactivate the viral reservoir and trigger effective antiviral immune responses. During the subsequent treatment interruption phase, 32% of the participants were able to control viral replication to levels below 2000 viral copies/ml. For further refinement of cure strategies and to increase the safety of such trials, there is an urgent need to identify non-invasive biomarkers that can predict viral control before ART interruption. The definition of such markers may also identify novel therapeutic targets that can improve future cure strategies. In this clinical setting, CD33/Siglec-3 emerged as a biomarker for predicting whether individuals will control or not control viremia during ART interruption, even when assessed in samples drawn at the baseline timepoint. Aside from defining CD33/Siglec-3 as an early predictor of virus control post treatment stop, our *in vitro* targeting experiments also support the use specific antibodies directed against CD33/Siglec-3 in future therapeutic trials. Since CD33 targeting has also found entry in the treatment of neurocognitive disorders, such an intervention in HIV infected individual may also help to stem against the cognitive impairments observed in HIV infection.

**Added value of this study**

In the present study, we observed that the administration of an HIV-1 therapeutic vaccine together with the latency reversing agent RMD has an impact on the plasma proteomic profiles, including inflammatory and neurological markers. In particular, we identify the CD33 protein, also known as sialic acid binding immunoglobulin-like lectin 3 (Siglec-3), as a soluble plasma marker that can discriminate MAP-C from MAP-NC already at baseline time points before RMD and vaccine administration. Validation analyses in an unrelated cohort of chronically HIV-1+ individuals and *in vitro* experiments suggest that CD33/Siglec-3 may play an important role in the HIV-1 life cycle and in vivo viral control and could be employed therapeutically.

**Implications of all the available evidence**

Our study identifies for the first time CD33/Siglec-3 as a biological marker of HIV-1 control in the absence of antiretroviral treatment and points towards its critical involvement HIV-1 infection. Other Siglec family members have been reported to be responsible for the capture and storage of pathogens by mature dendritic cells and for trans-infection between infected dendritic cells and target cells (e.g., Siglec-1 and Siglec-7). Here we identify plasma CD33/Siglec-3 levels as a biomarker associated with HIV-1 control during the clinical BCN02 trial but also in a large cohort of individuals with chronic, untreated HIV-1 infection. Moreover, addition of anti-CD33 monoclonal antibodies to *in vitro* virus cultures reduced HIV-1 replication, suggesting that CD33 may be required for HIV-1 replication. The use of CD33 monoclonal antibodies was previously studied in acute myeloid leukemia and in hepatitis B infection. Interestingly, several interventions targeting CD33 are also tested as potential therapies for Alzheimer’s disease (AD), as reduced cell surface CD33 levels allowed more efficient phagocytic clearance of pathogenic Amyloid beta (Aβ) and provide protection against disease. Our data thus suggest that further explorations and studies of the CD33/Siglec-3 protein could have significant implications for HIV-1 cure and also for the understanding and potential treatment of HIV-1 associated neurocognitive disorders.

## Introduction

Since its identification in the 1980’s, the human immunodeficiency virus HIV type 1 (HIV-1) represents a major global public health issue. Although combination antiretroviral therapy (ART) effectively suppresses HIV-1 replication and slows disease progression, there is no effective cure for HIV-1 infection. Moreover, ART is associated with viral resistance and treatment-related side effects and, even more importantly, is not accessible worldwide, reinforcing the urgent need for a HIV cure. During the last decade, different cure strategies have been explored, some of them based on latency reactivators, such as histone deacetylase inhibitors, which make host-cell chromatin more accessible to transcription factors. However their use can also impact host gene expression,^1^ which in the case of the Latency Reversal Agent (LRA) romidepsin (RMD), has previously been associated with beneficial effects on the central nervous system through the regulation of the synaptic plasticity.^2^

BCN02 was a single-arm, open-label clinical trial (NCT02616874) that enrolled 15 ART-suppressed early-treated, HIV-1+ individuals to receive a combination of 3 series of the LRA RMD and MVA.HIVconsv vaccines.^3^^,^^4^ The HIVconsv immunogen is a chimeric protein sequence assembled from 14 highly conserved domains derived in HIV-1 Gag, Pol, Vif, and Env.^3^, ^4^, ^5^ On the other hand, RMD has shown increased histone-3 acetylation levels causing a relaxation of the host chromatin and consequently favoring reactivation of latent HIV-1 virus in ex vivo experiments.^6^ Importantly, the BCN02 clinical trial also included a monitored antiretroviral pause (MAP) for a period of 32 weeks to evaluate the clinical efficacy of the intervention. Of 11 individuals evaluated during MAP, 8 individuals reached premature ART-restart criteria (MAP non controllers, MAP-NC) while 3 participants were able to control the virus throughout the entire 32 weeks MAP period to levels below 2000 HIV-1 copies/ml (MAP controllers, MAP-C).^3^ Interestingly, the outcome in BCN02 has also been linked to specific signatures in the host microbiome and the host gene epigenetic landscape, suggesting that possibly pre-existing particularities among HIV-1+ individuals may condition the success of the such therapeutic interventions.^7^^,^^8^

Although the BCN02 clinical trial included early treated individuals who initiated ART less than 6 months after infection and who therefore may present with smaller and less-diverse viral reservoirs, the virus systematically rebounds from this reservoir and is the major challenge for cure strategies. While the contribution of different anatomical compartments of the viral reservoirs to rebound remains unclear, it is known that after acquisition, HIV-1 also infects the central nervous system (CNS) and represents one of the anatomical compartments for latently infected cells.^9^ Infection of the CNS has also been related to the occurrence of HIV-1 associated neurological disorders (HAND)^10^ and eradication of this reservoir may pose specific challenges for LRA drug penetrability to the CNS. Interestingly, in animal studies the LRA RMD protected CNS through remodeling chromatin and regulating synaptic plasticity.^11^ RMD administration was also explored in Alzheimer’s disease (AD), where it increased the gene expression of neurotrophic factors and reduced AD-associated biochemical and cellular changes.^2^ Therefore, the inclusion of RMD in the BCN02 trial offered the opportunity to assess its effect on virus reactivation and neurological impact, including cognitive status, functional outcomes, and brain imaging evaluations in the trial participants.^12^ Although no alterations in neurocognitive or functional status and brain imaging have previously been shown in this study cohort,^12^ subjacent brain injury in asymptomatic forms cannot be discarded. Relevant to the present study, such subclinical events may be detectable in plasma or CSF fluids as shown in proteomics studies in HIV-1+ individuals suffering HAND, which have identified aberrations in several inflammatory and neurological damage markers.^13^, ^14^, ^15^

To further explore the impact of the therapeutic vaccination and LRA RMD on plasma markers of virus control and CNS integrity, longitudinally plasma proteome profiling was conducted in the BCN02 study samples. Overall, RMD administration and ART interruption strongly increased the protein levels of host factors involved in inflammatory processes but also that of markers of neurological disorders that all correlated with clinical, viral, as well as cognitive parameters. These longitudinal inflammatory/neurological profiles uniquely identified CD33/sialic-acid-binding immunoglobulin-like lectin 3 (Siglec-3), whose elevated plasma levels were associated with MAP non-control. Higher CD33/Siglec-3 plasma levels were also observed in individuals with high viral loads in an unrelated cohort with untreated HIV-1 infection. The clinical relevance of these findings is further supported by our observation that anti-CD33 monoclonal antibody *in vitro* blocked virus replication and integration, thus identifying CD33 as a potentially therapeutic target in HIV-1 infection.

## Methods

### Patients and samples

**Supplementary Figure S1:**
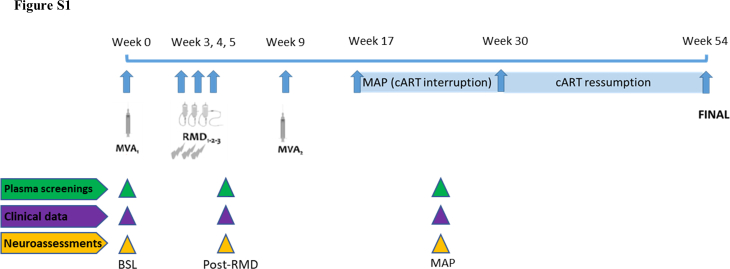
**BCN02 clinical trial scheme.** Schematic representation of the BCN02 clinical trial with sampling timepoints: baseline (BSL timepoint), three RMD infusions at week 3, 4 and 5 (week 6 is post-RMD timepoint) followed by ART interruption at week 17 until viral rebound or week 30 ART resumption (MAP timepoint). Week 54 end of the BCN02 clinical trial. Analyzed timepoints labeled with arrows: baseline (BSL), post-RMD infusions (post-RMD) and monitored antiretroviral pause phase (MAP), when plasma proteomics and neurological assessments were conducted.

The BCN02 clinical trial (NCT02616874) was a phase I, open-label, single-arm, multicenter study conducted in Barcelona (Spain).^3^ Fifteen HIV.consv vaccine recipients were rolled-over from the previous BCN01 trial,^16^ and were immunized with MVA.HIVconsv followed by three weekly-doses of RMD (5 mg/m^2^) and a second MVA.HIVconsv vaccination, before undergoing MAP for a maximum of 32 weeks (Supplementary Figure S1). During MAP, the participants restarted ART treatment if they reached the threshold of 2000 HIV-1 RNA copies/ml. Available plasma samples from 11 participants were used for the longitudinal proteomic profiling analysis, including 8 MAP-NC and 3 MAP-C who completed the 32 weeks of MAP (Supplementary Table S1). Chronic untreated HIV-1+ individuals (n = 96, Supplementary Table S2), enrolled at the IMPACTA clinics (Peru) and at Hospital Germans Trias i Pujol (Spain), were classified according to their degree of control of viral replication; HIV-high n = 47, >50,000 HIV-1 RNA copies/ml and HIV-low n = 49, <10,000 HIV-1 RNA copies/ml. The HIV high had a median CD4 count of 303 cells/mm^3^ (range 11–729 cells/mm^3^) while the HIV low had a median of 712 cells/mm^3^ (range 289–1343 cells/mm^3^). Blood samples from HIV uninfected donors from the Banc de Sang i Teixits in Barcelona were used for *in vitro* studies.

### Plasma proteomic profiling

BCN02 plasma samples were used for evaluation of inflammation, neurology and neuro-exploratory cytokine panels applying PEA (Proximity Extension Assays) by Olink® (https://www.olink.com). Briefly, a pair of oligonucleotide-labeled with specific antibodies binds to the protein present in sample, bringing the two oligonucleotide probes in close proximity and allowing for a specific sequence to be formed by DNA polymerization. The resulting sequence is detected and quantified using standard real-time PCR. Protein concentration was expressed as relative expression levels, using an arbitrary unit (normalized protein expression, NPX) on a Log_2_ scale. A high NPX value corresponds to a high protein concentration (Supplementary Table S3). For validation of relative CD33/Siglec-3 plasma levels in untreated HIV-1+ individuals, proteomic analysis were performed using customized arrays as previously described.^17^

### GEO access of BCN02 transcriptomics

CD33/Siglec-3 gene expression levels in total PBMC were obtained from previously published work.^8^ The used dataset is available in Gene Expression Omnibus (GEO), under the accession number GSE184653. The specific values for CD33/Siglec-3 were obtained after log_2_ transformation of the TMM normalized counts.

### Evaluation of CNS functioning

Neuropsychological evaluation covered 6 cognitive domains to provide a global composite score (NPZ-6), which was recorded as recently published.^12^ In brief, NPZ6 included: Digit Test of the Wechsler Adult Intelligence Scale (WAIS-IV); the Trail Making Test (TMT-A); Grooved Pegboard Test (GPT); California Verbal Learning Test (CVLT-II); Controlled Oral Word Test (COWAT); and the Trail Making Test (TMT-B).

### Determination of integrated HIV-1 proviral DNA

HIV-1 proviral DNA was quantified in total PBMC samples of untreated HIV-1+ individuals and in isolated CD4^+^ T cells of BCN02 clinical trial participants by droplet digital PCR (ddPCR) in duplicates, as previously reported.^18^ Briefly, two different primer/probe sets annealing to the 5′ long terminal repeat and Gag regions, respectively, were used to circumvent sequence mismatch in the patient proviruses, and the *RPP30* housekeeping gene was quantified in parallel to normalize sample input. Raw ddPCR data were analyzed using the QX100™ droplet reader and QuantaSoft v.1.6 software (Bio-Rad).

### Real-time PCR CD33 gene

RNA was isolated from available PBMC dry-pellets from participants in the untreated HIV-1 infection cohort and was retrotranscribed. TaqMan gene expression assay (Applied Biosystems) was used for *CD33* (Hs01076281_m1) detection. *TBP* gene (Hs99999910_m1) was used as the housekeeping gene. Gene amplification was performed in Applied Biosystems 7500 Fast Real-Time PCR System thermocycler, and the relative expression quantification was calculated as 2−ΔCT (where CT is the median threshold cycle from 3 replicates). CD33 gene expression was corrected by CD4 T cells count since this cohort of HIV-high and HIV-low individuals showed significant differences in the group-specific median CD4 T cell counts (Supplementary Table S2).

### HIV-1 infection of PHA-blasts and monocyte-derived macrophages

Isolated PBMCs from HIV-1 uninfected donors were stimulated with 5 μg/ml Phytohaemagglutinin (PHA, Sigma-Aldrich) and 10 U/ml IL-2 (Roche). After 3 days, PHA-blasts were infected with the HIV_NL4-3_ strain (Multiplicity of Infection (MOI): 0.01). For monocyte-derived macrophages (MDMs), PBMCs were depleted using the EasySep™ Human Monocyte Enrichment Kit (Stem Cell). Monocytes were then incubated with macrophage colony-stimulating factor (M-CSF, R&D systems S.L. #216-MC) at 1 μg/ml for 4 days before infection with the HIV_BaL_ strain (MOI: 0.01). HIV-1 infection in PHA-blasts and MDMs was evaluated under different conditions: Zidovudine (AZT, 200 μg/ml) was used as positive control of viral replication inhibition. The anti-CD33 mAb (Rabbit monoclonal [EPR4423], Abcam #ab134115) was used at concentrations ranging from 0.004 μg/μl, to 0.01 μg/μl. After 3 (PHA stimulated T cell blast) and 4 days (MDM), p24 in the culture supernatant was quantified by ELISA (INNOTEST HIV p24 Antigen mAb, Fujirebio, #80563). Total HIV-1 DNA quantification was done on total DNA isolated from cultured cell using the RNA/DNA purification Micro kit (Norgen Biotek Corp., #48700) as recommended by the manufacturer. Quantification of total HIV-1 DNA was determined by a quantitative PCR assay, using TaqMan Universal Master Mix II (Applied Biosystems) in a 7500 real-time PCR system (Applied Biosystems). We used the following set of primers and probe; forward primer: 5′-GACGCAGGACTCGGCTTG-3′ and reverse primer: 5′-ACTGACGCTCTCGCACCC-3′ and probe: 5′-fluorescein amidite (FAM)-TTTGGCGTACTCACCAGTCGCCG-6-carboxytetramethylrhodamine (TAMRA)-3′. *GAPDH* gene (Hs02786624_g1) was used as the housekeeping gene. Gene amplification was performed in an Applied Biosystems 7500 Fast Real-Time PCR System thermocycler, and the relative expression quantification was calculated as 2−Δ*CT* (where *CT* is the median threshold cycle from 2 replicates).

### STRING pathways

For Protein-Protein Interaction Networks, functional enrichment analysis was used to analyze interaction between proteins that showed significantly different concentrations between time points and between MAP-C and MAP-NC groups (https://string-db.org/cgi/input?sessionId=bcCYfoHsbNV0&input_page_active_form=multiple_identifiers). Specifically, for GO categories classification, counts in the network indicate how many proteins in the analysis are annotated in a particular GO term and are added to the graph.

### Statistical analysis

Univariate and multiple comparisons statistical analyses were performed using GraphPad Prism, version 8. For comparisons between patient groups, the Mann–Whitney and Wilcoxon signed rank tests were applied and for multiple group comparisons One-Way ANOVA test corrected for multiple comparisons with original False Discovery Rate (FDR) method using Benjamini and Hochberg method, were used. The Spearman and Pearson test were applied for the correlation analysis with non-parametric and parametric data, respectively. For all analyses, p-values <0.05 were considered statistically significant. Principal Component Analysis (PCAs), Heatmaps, Venn diagrams and correlograms were performed with RStudio version 1.2.5042.

### Ethics

The study was approved by the Comité Ètic d’Investigació Clínica of Hospital Germans Trias i Pujol (CEIC: EO-12-042 and PI-18-183) and all participants provided their written informed consent. All the research involving human research participants was performed in accordance with the Declaration of Helsinki.

### Role of the funding source

The funders of the study did not have any role in the design, data collection, data analyses, interpretation, or writing of the study.

## Results

### Plasma proteomes are impacted upon RMD administration

**Fig. 1:**
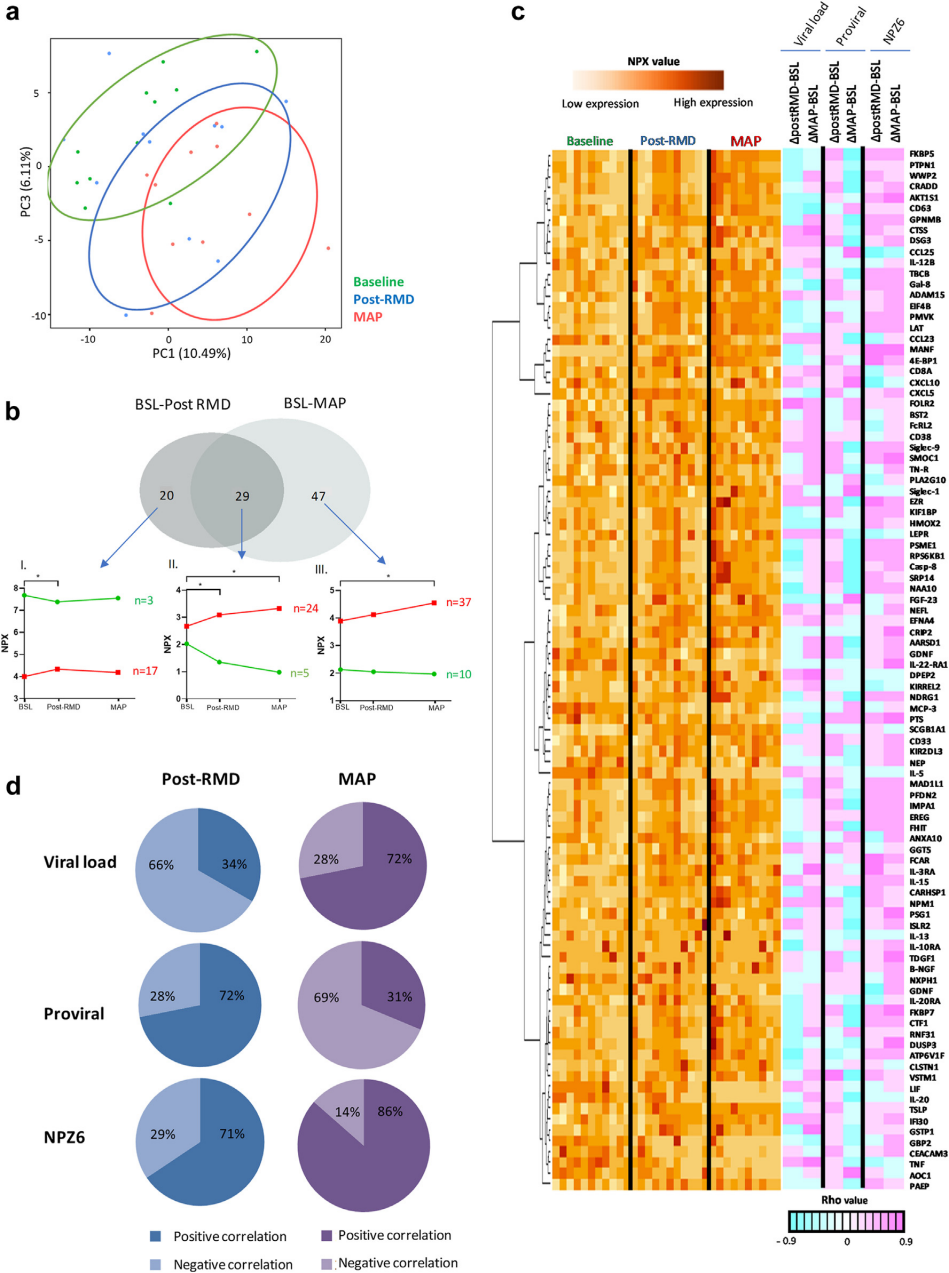
**Changes in plasma proteomes during the BCN02 clinical trial.** Soluble proteomes from 11 participants in the BCN02 trial at baseline (BSL), 1 week after 3 infusions of RMD (post-RMD) and during monitored antiretroviral pause (MAP) timepoints were determined using the proximity extension assay. a) Principal Component Analysis (PCA) reflecting the changes of 276 inflammatory and neurological-related plasma proteins during the BCN02 clinical trial at each tested timepoint. The BSL timepoint are is indicated in green, post-RMD timepoint in blue and MAP timepoint in red, each point representing one participant. b) Venn diagram representing differentially detected proteins when comparing BSL vs post-RMD (n = 49) and BSL vs MAP phase timepoints (n = 76). The different profiles indicate relative abundance of proteins that were up- or down-regulated: *Profile I*: Significant changes only detected when comparing BSL vs post-RMD infusions (n = 20); *Profile II*: Proteins that show significant changes when comparing BSL vs post-RMD as well as BSL vs MAP timepoints (n = 29); and *Profile III*: Significant changes only detected when comparing BSL vs MAP phase (n = 47). The x-axis marks the timepoints of the clinical trial and on the y-axis, the relative levels of the proteins, represented as the mean of NPX values for each protein. Longitudinal changes between BSL vs post-RMD and BSL vs MAP phase timepoints were assessed using Wilcoxon signed rank test. c) Heatmap showing the relative protein levels (NPX) of all significantly modulated proteins from (b) (p < 0.05, n = 96) per patient and timepoint (orange-yellow scale). Each column represents a patient measured at BSL, post-RMD and MAP. Additional columns (blue-pink scale, right of the heatmap) indicate the value of correlations (Spearman’s rho) between individual proteins and either plasma viral load, proviral levels or neurotest score (NPZ6). The Spearman's rank test was used for correlation analyses. d) Pie charts representing the percentages of number of proteins that are negatively or positively correlated with viral parameters (viral load and proviral levels, respectively) and with neurological parameters (NPZ6 evaluation matrix). Blue (post-RMD), purple (MAP), dark color indicates positive correlations and light color negative correlations. The Chi-square test was applied for categorizing positive or negative correlation. Statistical significance was set at p < 0.05.

Focused inflammatory and neurological-tailored proteomes were defined using PEA (Proximity Extension Assay) in plasmas samples from 11 BCN02 participants at baseline (BSL, week 0), 1 week after 3 infusions of RMD (post-RMD, week 6) and MAP timepoints (after MVA vaccination and while off ART; Supplementary Tables S1 and S3 and Supplementary Figure S1), to identify soluble markers modulated by RMD and therapeutic vaccination. Principal Component Analysis (PCA) was based on 276 soluble factors involved in inflammation and neurological processes and which were assessed across the three timepoints of the clinical trial. As shown in Fig. 1a, marked changes were observed after RMD treatment and were intensified during MAP. When compared to BSL, the administration of RMD modulated the plasma levels of 49 proteins, while 76 molecules were modulated during MAP levels (Fig. 1b, Supplementary Table S4). Of the 49 proteins that changed significantly upon RMD treatment, 29 were also modulated during MAP. Proteome profiling analysis of the 20 proteins differentially detected only between BSL and RMD administration timepoints, showed 17 molecules with an increase in relative protein levels (Fig. 1b Profile I. in red and Supplementary Table S4). Most of these proteins were related to “*cytokine release by the interaction with viral proteins*” such as CCL25, CXCL5, IL20RA and IL15 and “*innate and cellular adaptive immune response markers*” like CD8A and CD38. Three proteins showed decreased levels after RMD administration, including CXCL10, CCL23 and GDNF (Fig. 1b Profile I in green and Supplementary Table S4).

When comparing baseline vs MAP timepoints, 47 proteins showed different relative protein levels, with 37 showing and increase (Fig. 1b Profile III. in red, and Supplementary Table S4) and being mostly related with “*cell growth*”, “*proliferation*” or “*cell structure*” such as TBCB, PFDN2, 4E-BP1, NPM1, FGF23, CD63, among others. The 10 proteins that decreased their relative levels over this time frame (Fig. 1b Profile III. in green and Supplementary Table S4) were related to “*response to cytokine*” category like IL20, IL22RA1, LIF, GBP2, SCGB1A1 and NEP and “*regulation of epithelial cell differentiation*” such as GDNF, LIF and IL20.

In addition, the 29 proteins that showed significant changes between both, baseline vs post-RMD and baseline vs MAP comparisons (Supplementary Table S4), were progressively and significantly increased (24 molecules) or decreased (5 proteins) over-time. Most of the increased 24 proteins (Fig. 1b Profile II. In red, Supplementary Table S4) were involved in “*neuronal and axonal functions*” like NEFL, MANF and CLSTN1, while the other 5 molecules that decreased included MCP3, IL5, TNF, CEACAM3 and TDGF1, Fig. 1b Profile II. in green, Supplementary Table S4).

To identify potential drivers of the observed changes in protein levels from baseline to post-RMD and/or MAP timepoints, a correlation analysis was performed between relative protein levels and virological (plasma viral load (pVL) and proviral levels). Correlation analysis also include neurological parameters (NPZ6 (Fig. 1c and d) since RMD treatment has been reported to affect these parameters. The analyses showed that after RMD infusions, 34% of plasma proteins correlated positively and 66% molecules negatively correlated with pVL. During MAP timepoint these percentages changed as now 72% of the proteins positively associated and 28% negatively associated with pVL (Fig. 1c and d and p = 0.0001, chi squared test). In contrast, at post-RMD timepoint the 72% percent of the plasma proteins that were differentially detected between timepoints were positively associated (72%) and only the 28% negatively associated with proviral levels, while during MAP phase these percentages were partially inverted to 31% positively and 69% negatively associated with proviral levels (Fig. 1c and d p = 0.0001, chi squared test).

These data suggest that some proteome profiles at post-RMD are likely reflecting the levels of integrated virus as the participants are on cART treatment, while during MAP, the observed changes mostly reflect the increased viral replication and the activation of the “*interferon signaling pathway*”, with proteins such as IL15, CXCL10, IFI30, TNF, IL3, IL12, IL20 being more abundant.

Interestingly, correlation analysis with neurological parameters (NPZ6) showed that 71% of the molecules correlated positively with NPZ6 neurocognitive test after RMD treatment and during MAP this proportion rose to 85% (p = 0.0083, chi squared test), reflecting the higher number of proteins that was associated with neurological parameters (such as MANF, GDNF, NEFL GPNMB (Fig. 1c) at the completion of the intervention and MAP.

### Plasma proteomes at baseline and after RMD differentiate eventual MAP-controllers and MAP-non-controllers

**Fig. 2:**
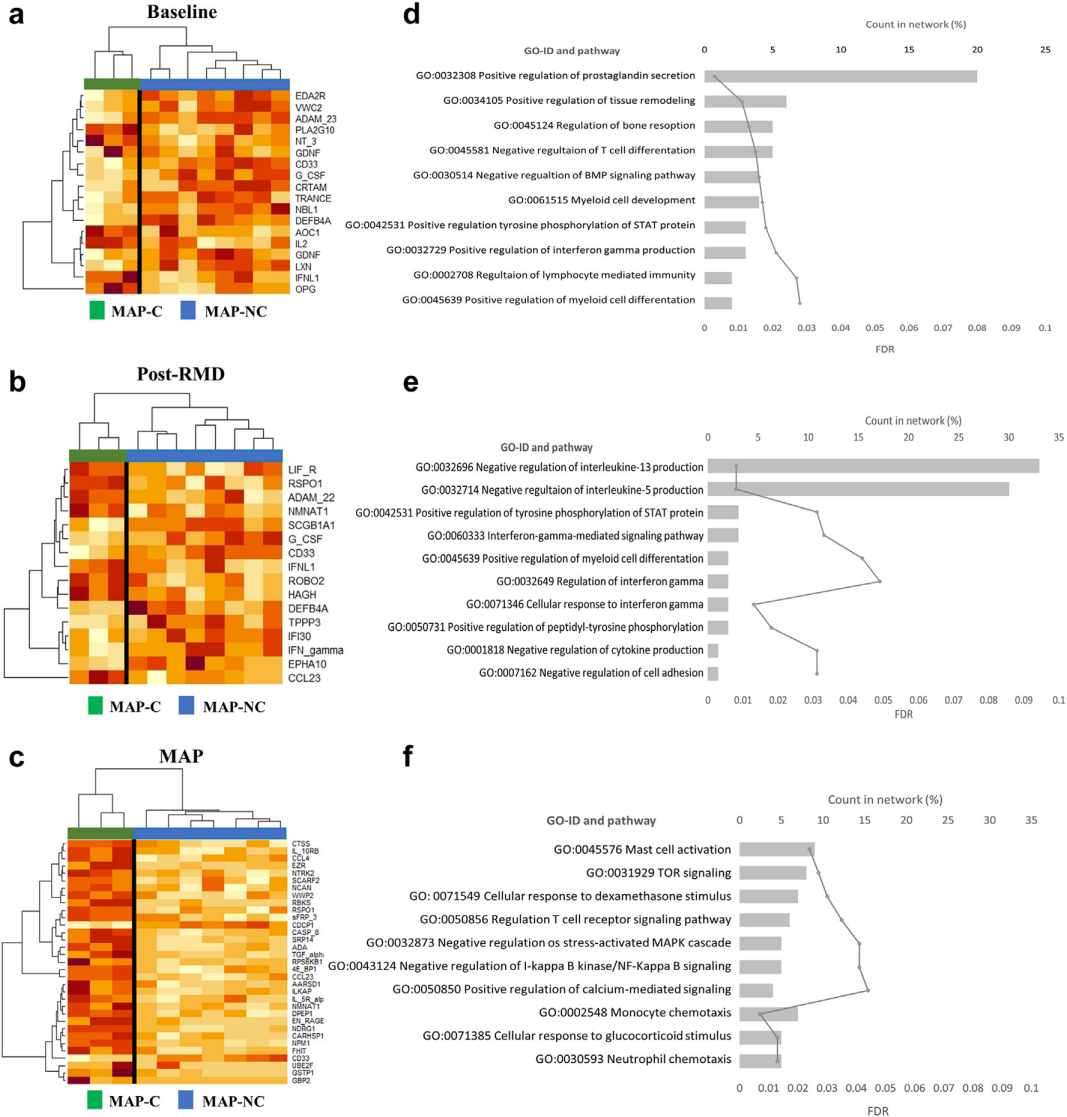
**Differential plasma proteomes between MAP-C and MAP-NC.** a–c) Heatmap of relative protein plasma levels (NPX) of proteins differentially detected between MAP-C and MAP-NC at (a) baseline, (b) post-RMD and (c) the MAP phase of the BCN02 clinical trial. Orange-yellow scale indicates NPX values. Green represents MAP-C and blue MAP-NC participants. d–f) Gene Ontology (GO) classification of proteins that differed significantly between MAP-C and MAP-NC at each timepoint. On the y-axis, gene ontology categories are indicated, on the upper x-axis, counts in network are noted to indicate how many proteins in our network are annotated with a particular term (GO, represented as percentage in histogram) and on the lower x-axis, as continuous line, the false discovery rate (FDR) representation.

**Supplementary Figure S2:**
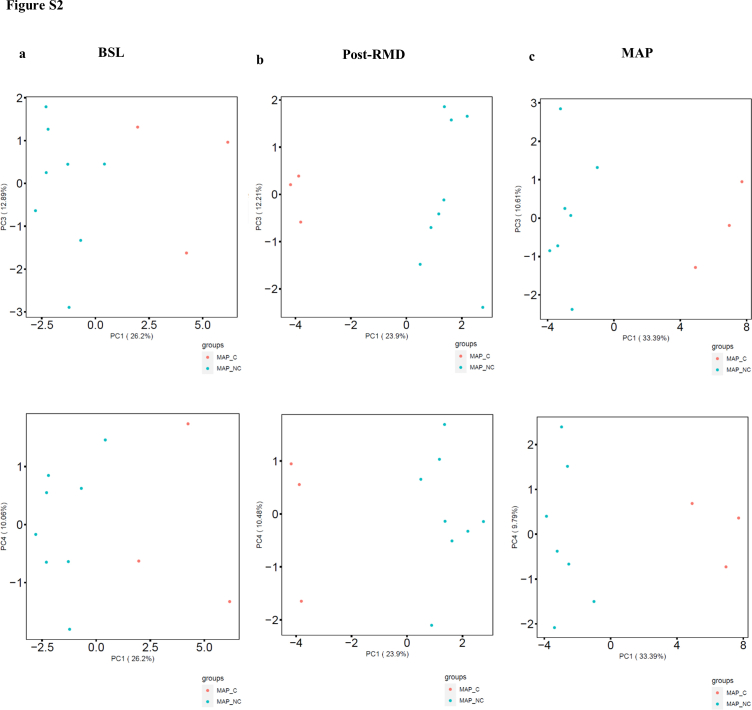
**Principal component analysis (PCA) differentiates MAP-C and MAP-NC.** a–c) PCA plots of differentially detected proteins between MAP-C and MAP-NC at a) baseline, b) post-RMD infusions and c) MAP phase. Orange dots indicate MAP-NC and green dots MAP-C participants. Upper plots show PC1 (x-axes) vs PC3 (y-axes) and lower plots show PC1 (x-axes) vs PC4 (y-axes).

As plasma proteomes were associated with viral loads and proviral levels, we next assessed whether plasma proteome signatures at each timepoint (baseline, post-RMD and MAP) would have the power to differentiate individuals who can or cannot control viral replication during MAP. To that end, we compared the plasma proteomes of MAP-NC individuals (>2000 HIV-1 RNA copies/ml and thus restarting ART) and MAP-C (>32 weeks <2000 HIV-1 RNA copies/ml) at all three timepoints tested (baseline, post-RMD and MAP). PCA plots showed that proteome profiles at all timepoints, including baseline, segregated the two groups (Supplementary Figure S2a–S2c). Specifically, the comparative analysis between MAP-C and MAP-NC by timepoints showed that differences in the proteome of both groups were observed at baseline (18 proteins, Fig. 2a), post-RMD (16 molecules, Fig. 2b) and, with broader profiles, during MAP (33 molecules, Fig. 2c).

To gain insights into the differences at biological level between the observed signals at each timepoint, Gene Ontology (GO) classification was performed (Fig. 2d–f). Aside from the most represented prostaglandin-related ontology, the analysis at baseline indicated a differential representation of genes involved in “*T cell differentiation*”, “*lymphocyte immunity*”, “*interferon-gamma production*” and “*myeloid cell development*” ontologies (Fig. 2d). After RMD administration, the major gene ontologies differentially represented between MAP-C and MAP-NC included “*interferon pathways*” and “*negative regulation of IL5 and IL13 production*” ontologies (Fig. 2e). “*Innate cellular (Mast cells, Neutrophils and Monocytes)*”, “*T cell immunity*” categories and the involvement of “*NF-KB and MAPK cascades*” were the major ontologies differentially represented between both groups at the MAP timepoint (Fig. 2f). Although some proteins involved in neurological processes were significantly altered by the intervention, the two groups (MAP-C and MAP-NC) did not show any distinguishable features in their neurological protein profile at any of the tested time points.

### CD33/Siglec-3 is the unique discriminative plasma factor between MAP-C and MAP-NC across BCN02 trial

**Fig. 3:**
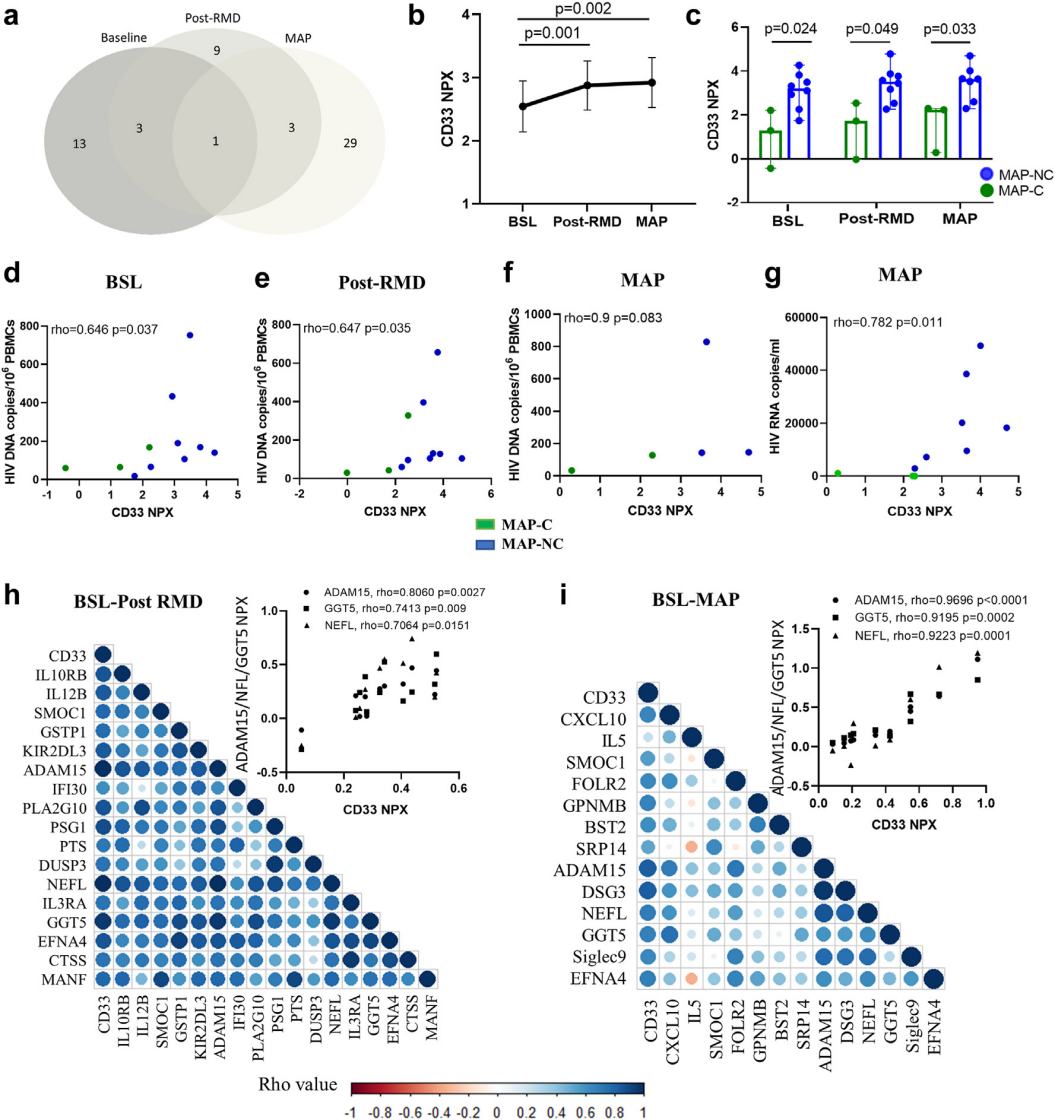
**CD33 plasma protein levels differentiate MAP-C and MAP-NC across the clinical trial.** a) Venn diagram showing differentially detected proteins between MAP-C and MAP-NC in samples from each timepoint of the clinical trial (BSL, post-RMD and MAP). b) Longitudinal representation of the relative plasma CD33 levels (NPX, y-axis) at each timepoint (x-axis). Values are shown as mean and standard error. Longitudinal changes over time were assessed using Wilcoxon signed rank test. c) Cross-sectional analysis of the relative CD33 plasma levels in MAP-C (green color) and MAP-NC (blue color) individuals over the duration of the clinical trial. Clinical trial timepoints are shown on the x-axis, and relative plasma levels of CD33 shown on the y-axis. Values are shown as median and max and min. Group changes per timepoint were assessed using the Mann–Whitney *U* test. d–f) Correlation analysis between relative CD33 protein levels (NPX, x-axis) vs HIV proviral DNA levels (HIV DNA copies/10^6^ PBMCs, y-axis) are shown on the y-axis in each timepoint of the study including (d) baseline, (e) post-RMD and (f) MAP phase. The Spearman's rank test was used for correlation analyses. g) Correlation analysis showing the association between relative CD33 protein levels (NPX, x-axis) vs plasma viral load (HIV RNA copies/ml, x-axis) in MAP phase. Green dots indicate MAP-C and blue dots indicate MAP-NC. The Spearman's rank test was used for correlation analyses. h and i) Correlogram plots of the significantly changed proteins detected between BSL vs post-RMD (h) and BSL vs MAP phase (i) that correlate with CD33 relative plasma levels. Colored dots indicate the level of correlation (blue: positive correlation and red: negative correlation). Upper graphs indicate the strongest associations observed between CD33 and 3 proteins (NEFL, GGT5 and ADAM15) in the neurology panel. The Spearman's rank test was used for correlation analyses. Statistical significance was set at p < 0.05.

**Supplementary Figure S3:**
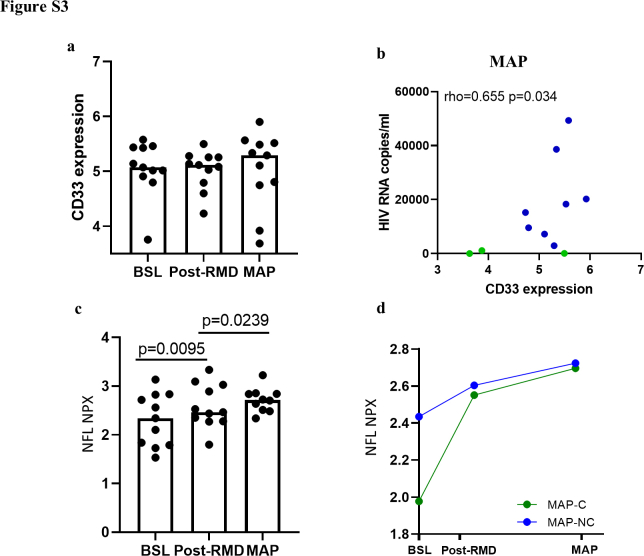
***CD33 and NEFL* gene expression during the BCN02 clinical trial.** a) *CD33* gene expression levels at baseline (BSL), post-RMD infusions (post-RMD) and at MAP phase (MAP). The upper limit of the bar is the median value of CD33 plasma levels. Longitudinal changes over time were assessed using Wilcoxon signed rank test. b) Correlation between *CD33* gene expression levels and plasma viral load in the MAP phase. MAP-C individuals are labeled in green and MAP-NC individuals in blue, respectively. The Spearman's rank test was used for correlation analyses. c) Relative NEFL plasma levels (NPX) at baseline (BSL), post-RMD infusions (post-RMD) and at MAP phase (MAP). The upper limit of the bar is the median value of relative NEFL plasma levels. Longitudinal changes over time were assessed using Wilcoxon signed rank test. d) Plot representing relative NEFL plasma levels in MAP-C (green line) and MAP-NC (blue line) individuals over the clinical trial. Weeks of treatment are shown on the x-axis, and relative NEFL plasma levels shown on the y-axis. Values are shown as median and standard deviation. Differences between MAP-C and MAP-NC groups per timepoint were assessed using the Mann–Whitney *U* test. Statistical significance was set at p < 0.05.

When analyzing the proteome profiles discriminating MAP-C and MAP-NC, we observed that CD33/Siglec-3 was the unique protein consistently differentially detected between the two groups across all three timepoints tested (Fig. 3a). Moreover, the plasma levels of CD33 were significantly changed during the intervention (Fig. 3b, BSL vs post-RMD p = 0.001 and BSL vs MAP p = 0.002, Wilcoxon test) and, despite the small number of participants in the trial, were significantly elevated in MAP-NC compared with MAP-C in all three timepoints (Fig. 3c, BSL: p = 0.024, post-RMD: p = 0.049 and MAP: p = 0.033, Mann–Whitney test). In addition, CD33 protein plasma levels positively correlated with proviral copy numbers at baseline and after RMD administration (Fig. 3d and e, BSL rho = 0.646 p = 0.037, post-RMD rho = 0.647 p = 0.035, Spearman rank test). A trend towards a positive association with viral reservoir was also observed at MAP timepoint (Fig. 3f, MAP rho = 0.9 but with p = 0.083 (Spearman rank test) not reaching significance, likely since proviral assessments were missing for more than half of the participants at the MAP timepoint. Still, MAP samples showed also a positive correlation between CD33 protein levels and pVL (Fig. 3g, rho = 0.782 p = 0.011, Spearman rank test). To investigate whether cells in the peripheral blood are the major source of CD33 proteins detected in the plasma, we assessed CD33 gene expression levels in PBMCs. The positive correlation between CD33 expression and plasma viral load levels (Supplementary Figure S3b, Spearman rank test), is indeed suggestive of PBMCs be a major source of the CD33 protein in plasma (Supplementary Figure S3a, Wilcoxon rank test).

To gain further inside into the mechanisms by which CD33 could influence viral control, we performed a correlation analysis between CD33 and other proteins that were included in the inflammatory, neurology or neuro-exploratory cytokine panels and which significantly changed during the intervention. After RMD administration, CD33 protein levels were strongly associated with 17 proteins, most pronouncedly with ADAM15, GGT5 and NEFL (Fig. 3h, ADAM15 rho = 0.806 p = 0.0027, GGT5 rho = 0.7413 p = 0.009 and NEFL rho = 0.7064 p = 0.0151, Spearman rank test). These associations were not only maintained but statistically even more pronounced at the MAP timepoint (Fig. 3i, ADAM15 rho = 0.9696 p < 0.0001, GGT5 rho = 0.9195 p = 0.0002 and NEFL rho = 0.9223 p = 0.0001, Spearman rank test). Interestingly, the NEFL protein, is a well-established marker for neurological damage in several diseases,^19^^,^^20^ and tend to increase the levels during MAP phase. These changes did not reach statistical significant difference between MAP-C and MAP-NC groups (Supplementary Figure S3d and S3e, Wilcoxon rank test and Mann–Whitney test, respectively).

Overall, these data indicate that CD33 protein levels differ between MAP-C and MAP-NC, even before the BCN02 intervention and that this difference is further pronounced after RMD treatment and showed strong positive correlations with viral parameters and markers of neurological damage.

### CD33/Siglec-3 validation in chronic untreated HIV-1 infected cohort

**Fig. 4:**
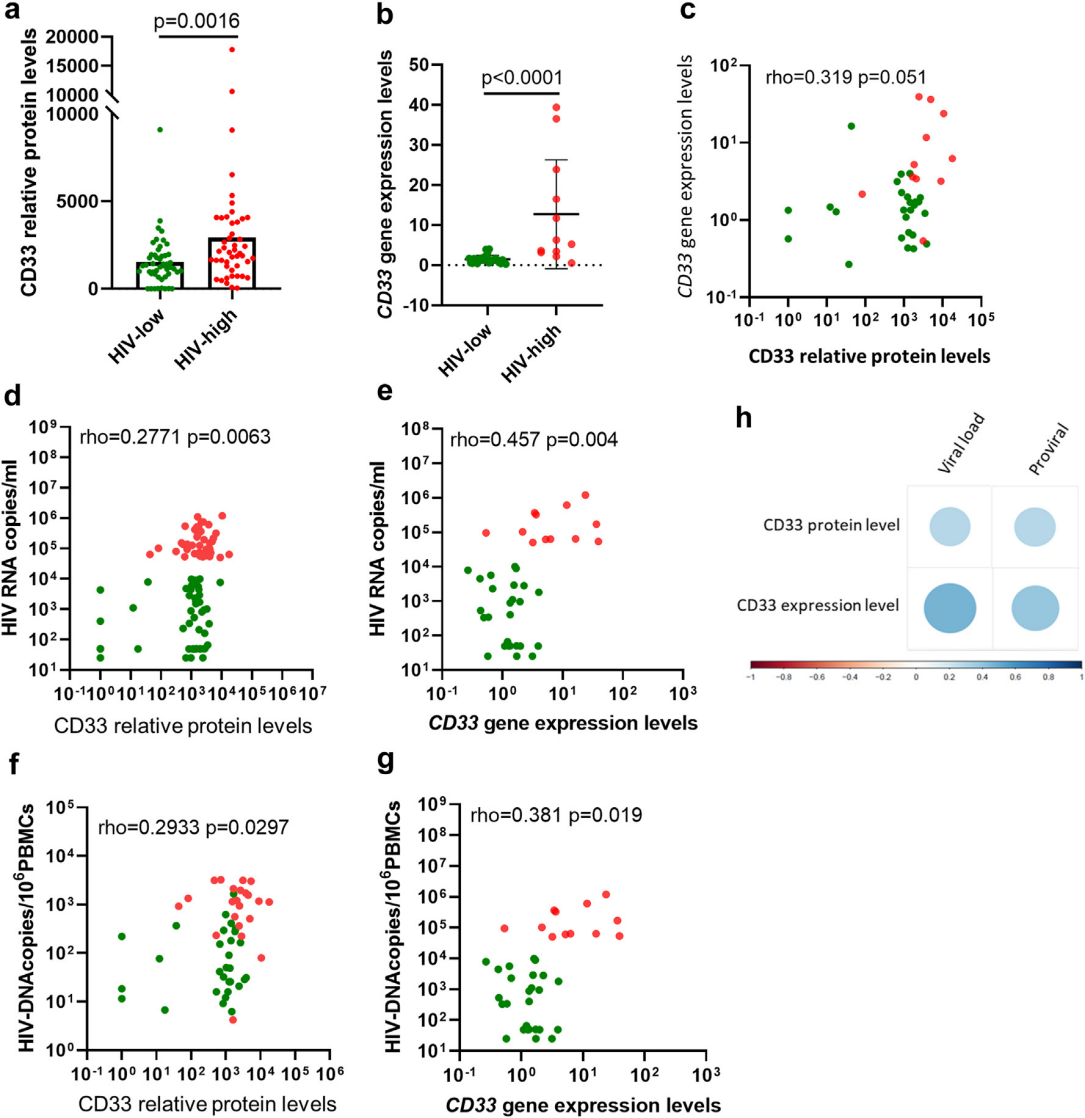
**CD33 levels in natural untreated HIV infection.** Proteomic array using plasma samples from ART-naïve, chronically HIV-1 infected individuals with different degrees of HIV control (n = 96), categorized as HIV-high and HIV-low according to their viral load. a) Scatter plot showing the plasma CD33 protein levels (y-axis) in both groups (x-axis); HIV-low (n = 49, green dots) and HIV-high (n = 47, red dots). Mann–Whitney *U* test was applied between HIV-high and HIV-low. b) *CD33* gene expression (y-axis, relative *CD33* gene expression corrected for CD4 counts) measured in PBMCs of HIV-high (n = 12, red dots) and HIV-low (n = 25 green dots) (x-axis) individuals. Mann–Whitney *U* test was applied between HIV-high and HIV-low. c) Correlation analysis between *CD33* gene expression (corrected for CD4 counts, y-axis) and CD33 protein levels in plasma considering all individuals. The Spearman's rank test was used for correlation analyses. d and e) Correlation analysis between (d) CD33 plasma protein levels and (e) *CD33* gene expression levels (x-axis) and viral load levels (HIV RNA copies/ml, y-axis). HIV-high, n = 47, red dots and HIV-low, n = 49, green dots. The Spearman's rank test was used for correlation analyses. f and g) Correlation analysis between (f) CD33 plasma protein levels and (g) *CD33* gene expression levels (x-axis) and HIV proviral DNA levels in total PBMC (HIV DNA copies/10^6^ PBMCs, y-axis). Considering all the individuals (HIV-high, n = 47, red dots and HIV-low, n = 49, green dots. The Spearman's rank test was used for correlation analyses. h) Correlogram plot between CD33 levels in plasma and PBMC expression and viral parameters. CD33 differences between groups were analyzed using the Mann–Whitney test. The Spearman's rank test was used for correlation analyses. Statistical significance was set at p < 0.05.

**Supplementary Figure S4:**
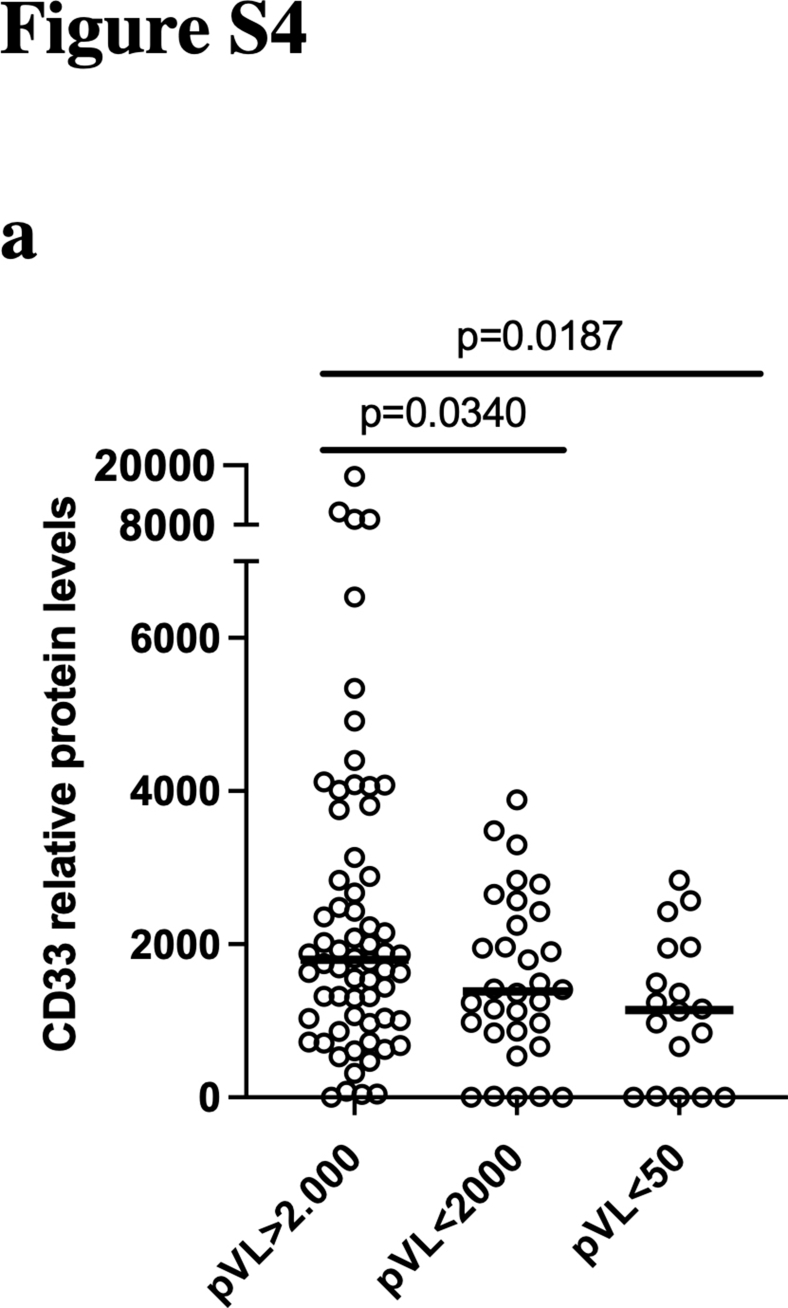
**CD33 levels in natural untreated HIV infection.** Scatter plot showing the plasma CD33 protein levels (y-axis) in individuals stratified by viral load either >2000 HIV-RNA copies/ml) or <2000 HIV-RNA copies/ml) and in the Elite Controllers group (<50 HIV-RNA copies/ml) (x-axis). ANOVA test corrected for multiple comparisons Original FDR method of Benjamini and Hochberg was used to test for differences between the three groups.

To validate the relationship between CD33 plasma levels and virus control, we tested samples from a cohort of untreated HIV-1+ individuals with different levels of virus control by determining plasma protein and gene expression levels of CD33. This ART-naïve, chronically HIV-1 infection cohort included HIV-1+ individuals with high (defined as “HIV-high”, n = 47, pVL >50,000 HIV-1 RNA copies/ml) or low (defined as “HIV-low”, n = 49, pVL <10,000 HIV-1 RNA copies/ml) plasma viral loads. Significantly higher CD33 plasma protein levels were detected in HIV-high compared to HIV-low individuals (Fig. 4a, p = 0.0016, Mann–Whitney test). Moreover, applying the BCN02 trial criteria for ART restart (2000 HIV RNA copies/ml) to this comparison showed that untreated HIV-infected individuals with <2000 HIV RNA copies/ml and <50 HIV RNA copies/ml to have significantly reduced levels of CD33 compared to individuals with >2000 HIV RNA copies/ml (p = 0.0340 and p = 0.0187, respectively, ANOVA test corrected for multiple comparisons) (Supplementary Figure S4). As in the BCN02 participants, CD33 gene expression in PBMCs from this cohort, showed higher *CD33* gene expression levels in HIV-high individuals compared with HIV-low (Fig. 4b, p < 0.0001, Mann–Whitney test). CD33 proteomic and gene expression levels at PBMCs were not significant correlated (Fig. 4c, rho = 0.319 p = 0.051, Spearman rank test). As observed in the MAP phase of the BCN02 study, CD33 plasma levels and gene expression levels also correlated positively with plasma viral loads (Fig. 4d, e and h, CD33 protein levels vs pVL rho = 0.2771 p = 0.0063, and CD33 gene expression levels vs pVL rho = 0.457 p = 0.004, Spearman rank test) and HIV-1 proviral copy numbers (Fig. 4f–h, CD33 protein levels vs proviral rho = 0.2933 p = 0.0297, and *CD33* gene expression levels vs proviral rho = 0.381 and p = 0.019, Spearman rank test). These data further indicate that CD33/Siglec-3 is closely related with in vivo viral replication and may serve as a predictor of the outcome after treatment interruption and/or therapeutic interventions.

### *In vitro* CD33 targeting reduces HIV-1 replication and virus reactivation

**Fig. 5:**
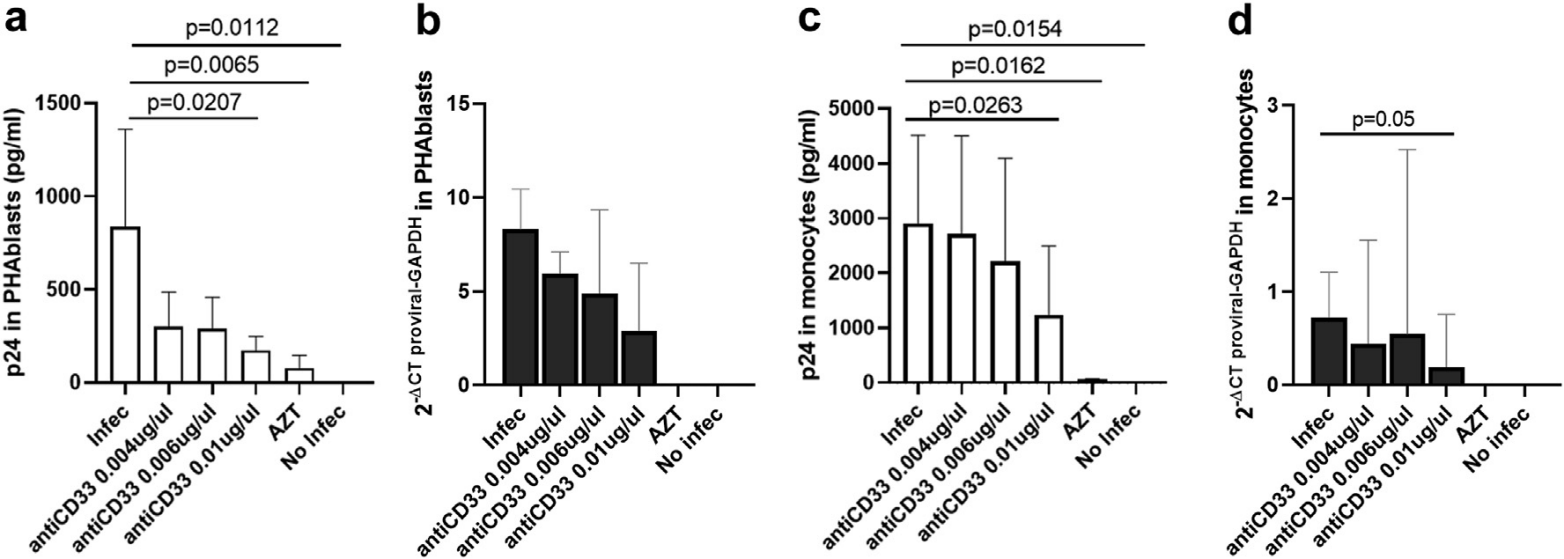
**Targeting CD33 reduces HIV replication and provirus levels.** a and c) Inhibition of HIV replication in the presence of anti-CD33 mAb, tested in (a) PHA-stimulated T cells infected with the HIV_NL4-3_ strain; (c) HIV infected monocyte-derived macrophages infected with the HIV_BaL_ strain. Experimental conditions are shown on the x-axis and quantification of absolute p24 supernatant (pg/ml) is shown on the y-axis. b and d) Total HIV-1 DNA quantification in cells from the same T cell and MDMs experiments as in (a) and (c). Experimental conditions are shown on the x-axis and quantification of total HIV-1 DNA is shown on the y-axis. All the plots show the median and standard deviation of six (PHA-stimulated T cells) and five (monocyte-derived macrophages) independent experiments in duplicates for each condition. For HIV infected T cells and MDMs, One-Way ANOVA test corrected for multiple comparisons. Original FDR method of Benjamini and Hochberg was used to analyze differences between conditions. For all comparisons, p < 0.05 was considered statistically significant.

**Supplementary Figure S5:**
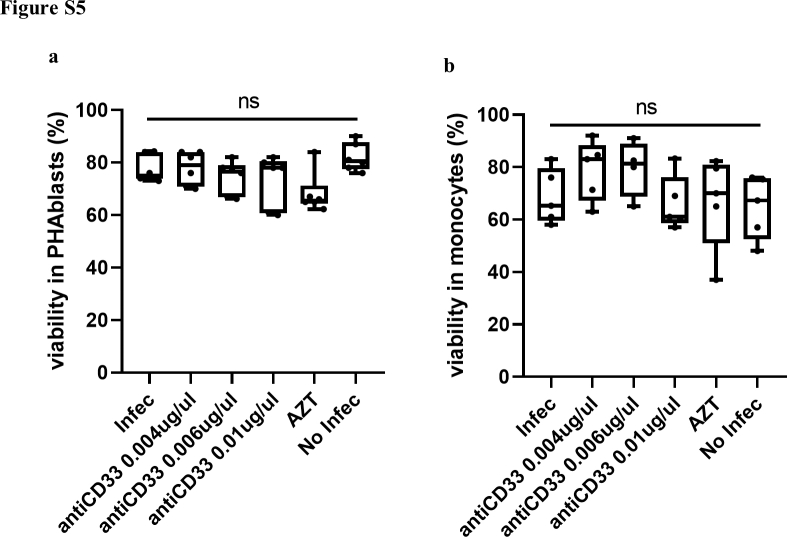
***In vitro* cell viability in cultures with anti-CD33 mAb.** Cell viability in the presence of increasing concentration of the anti-CD33 mAb, tested in (a) HIV infected PHA-stimulated T cells (six independent experiments) with HIV_NL4-3_ strain; and in (b) monocyte-derived macrophages (five independent experiments) infected with HIV_BAL_ strain. Experimental conditions are shown on the x-axis; quantification of viability (% live cells) is shown on the y-axis. For HIV infected T cells and MDMs, One-Way ANOVA test corrected for multiple comparisons Original FDR method of Benjamini and Hochberg was used to analyze differences between conditions. For all comparisons, p < 0.05 was considered statistically significant.

To investigate more directly the potential involvement of CD33 in HIV-1 infection and viral reservoir, we tested whether targeting CD33 *in vitro* on T cells and monocyte-derived macrophages (MDM) would impact viral infection and replication. Indeed, adding an anti-CD33 mAb to HIV-1_NL4-3_–infected PHA-stimulated T cell cells reduced virus production in a dose-dependent manner (Fig. 5a One-Way ANOVA test) without affecting the cell viability (Supplementary Figure S5a, One-Way ANOVA test). In parallel, the amount total HIV-1 DNA was also decreased in the presence of an anti-CD33 mAb (Fig. 5b, One-Way ANOVA test). The same was observed when HIV-1_BaL_-infected MDMs were cultured in presence of anti-CD33, with HIV-1 replication being reduced in a dose-dependent manner (Fig. 5c p = 0.0263 and Supplementary Figure S5b, One-Way ANOVA test). The reduction in proviral copy numbers in monocytes reached statistical significance as well (Fig. 5d, One-Way ANOVA test). Together, these data suggest that targeting CD33 can reduce HIV-1 replication and/or infection, indicating that CD33 at some point of the HIV-1 viral life cycle is required for effective viral propagation.

## Discussion

The BCN02 clinical trial was a proof of concept of a “kick-and-kill” strategy employing the RMD and HIVconsv vaccination.^3^^,^^4^ A series of previously published data of BCN02 trial results have demonstrated that this intervention caused marked effects on the organism at immunological,^4^ microbiome,^7^ epigenetic^8^ and neurological levels.^12^ Here, we explored the impact of the administration of RMD and therapeutic vaccination on the peripheral blood proteome to identify soluble biomarkers that can predict the outcome of this intervention. Our longitudinal proteome analysis revealed that the administration of RMD increased the abundance of several plasma proteins involved in the inflammatory immune response and affecting neurology processes, and that most of these changes were carried into the ART interruption phase of the study. As a histone deacetylation inhibitor, RMD causes relaxation of the host cell chromatin and enhances gene expression, of both, the integrated virus as well as host genes.^8^ The latter can translate into higher secretion of proteins into peripheral blood plasma, and can at least partly be reflected by changes in different organs, including the CNS. In addition, “kick-and-kill” interventions could also generate increased cell death by induction of apoptotic proteins including CASP-8 or TNF. In line with these data, previous analyses in the BCN02 trial showed that activated T cells were sensitive to RMD exposure as increased levels of apoptosis in CD4^+^ and CD8^+^ were detected.^4^ This was observed especially during the MAP phase of the BCN02 trial, suggesting that part of the soluble plasma proteome detected may stem from cell death caused by RMD toxicity, reactivated viral replication and/or cell lysis due to boosted cytolytic immune mechanisms.^4^ These different effects may also help explain the results of our correlation analyses, where the majority of the differentially expressed proteins after RMD administration showed positive correlations with proviral levels, while during MAP, most positive associations were observed for plasma viral loads.^21^

As RMD has been reported to have beneficial effects in some neurological diseases^2^ and one of the anatomical compartments where the latent HIV-1 reservoir is established is the CNS,^22^ the participants in the BCN02 trial also underwent various cognitive evaluations.^12^ No harmful effects on cognitive status, functional outcomes or brain imaging parameters were observed after RMD. In addition, CNS safety has also been confirmed previously after completion of the MAP.^12^ Despite RMD apparently being well-tolerated, the plasma proteomics revealed the levels of two neuroprotective proteins (MANF and GDNF^23^^,^^24^) to be increased by RMD, and two neurological damage markers (NEFL and GPNMB^25^^,^^26^) being increased during the MAP phase. These observations suggest that RMD did indeed affect the CNS, either directly or through the induction of pro-inflammatory responses and/or the reactivation of the viral reservoir.

Aside from longitudinally evaluating the effects of RMD and MAP on the plasma proteomes and markers of neurodamage, we also tested whether these plasma signature could inform on eventual virus control during MAP. The existence of biomarkers predictive of post-treatment virus control is supported by a recent study describing specific plasma metabolic signatures associated with virus control after HIV-1 treatment interruption^27^ and by reports demonstrating increased expression of some restriction factors genes such as APOBEC3G and SLFN11^28^ or immunological features such as PD-1, Tim-3 and Lag-3 prior to ART initiation that strongly predicted time to viral rebound.^29^ Furthermore, our recent studies have shown that BCN02 MAP-C have a host-gene epigenetic landscapes^8^ as well as microbiome compositions^30^ prior to the start of the trial that were predictive of viral control during MAP. However, plasma biomarkers associated with control of viremia in a context of a “kick-and-kill” strategy have not been identified. The present data show that prior to the BCN02 intervention, baseline plasma proteomes were different between MAP-C and MAP-NC, with proteins related to adaptive and innate immunity being elevated in MAP-NC. Of note, the prostaglandin-related gene ontology was the most represented one for the discrimination between MAP-C and MAP-NC. Particularly, MAP-C showed higher levels of plasma PLA2G10 protein than MAP-NC. It is tempting to speculate that MAP-C have higher antiviral capabilities due to the higher levels of PLA2G10, as it has been previously shown that PLA2G10 has the ability to neutralize HIV-1 through the degradation of the viral membrane and to inhibit HIV-1 replication in CD4 T cells.^31^ It will be important to explore how the markers involved in prostaglandin-related ontology, particularly PLA2G10, determine the response to cure strategies and how they could can be leveraged to achieve improved control rates in future trials.

Interestingly, CD33/Siglec-3 protein was the unique plasma marker, whose levels were increased upon RMD administration and maintained when ART was stopped. In addition, CD33 plasma levels allowed for the discrimination of MAP-C and MAP-NC individuals, already at the baseline timepoint. Intriguingly, CD33/Siglec-3 can modulate immune responses by inhibiting monocytes,^32^ but can be also expressed on dendritic cells and in a lesser extent on activated T cells and natural killer cells. The binding with its ligands; C1q and sialylated glycoproteins (the latter also highly expressed in brain microglia), leads to the recruitment of inhibitory proteins initiating signaling cascades that inhibit cell activation and functions associated with cytokine release or virus phagocytosis.^32^ Previous reports have shown that homeostatic basal levels of plasma CD33 are low but are strongly increased under pathogenic conditions, including viral infections.^32^ As cell surface CD33 is an interferon inducible factor,^33^ higher viral burden could induce its production, and in more extreme situation, shedding of CD33 into the plasma, as has been described for another member of the Siglec family (Siglec-7) in uncontrolled HIV-1 infection. Of note, pre-incubation of HIV-1 particles with soluble Siglec-7 increased the infection rate of CD4 T cells, which do not constitutively express Siglec-7 at the surface.^33^ Moreover, PLWH showed higher plasma levels of Siglec-7 compared to seronegative individuals and these plasma levels correlated positively with HIV-1 viral loads.^33^ It is thus plausible to think that increased levels of CD33 detected in MAP-NC and HIV-high participants could accelerate HIV propagation. This would be in line with our findings of higher CD33 protein plasma levels in MAP-NC than MAP-C, even if total PBMC transcriptomics analysis in this small set of BCN02 participants did not reach in all cases statistical significance. Also, similarly to Siglec-7 study, CD33 plasma levels were positively associated with pVL and proviral levels in the BCN02 trial, especially during MAP when levels of circulating virus are elevated.

In addition to its effects in viral infections, CD33 has been implicated in neuroinflammation, where its activation reduces phagocytic activity of microglia cells, causing an accumulation of pathogenic Aβ plaques.^32^^,^^34^ In our present study, we indeed observed very strong associations between CD33 plasma levels and the gamma-glutamyltransferase 5 (GGT5), ADAM metallopeptidase domain 15 (ADAM15) and neurofilament light protein (NEFL). In particular, NEFL has been reported to be a biomarker of Alzheimer disease,^35^^,^^36^ where CD33 may drive impaired phagocytic capacity of microglia and pose a risk for Alzheimer's disease.^32^ Furthermore, our previous BCN02 epigenetic analyses revealed differential DNA methylation positions (DMPs) in the *CD33* gene comparing MAP-C and MAP-NC. As one of these DMPs mapped to a Single Nucleotide Polymorphism (SNP) associated with AD risk,^37^^,^^38^ it will be interesting to assess to what degree CD33 influences neurological disease progression in HIV-1+ individuals suffering from HAND. Of note nowadays, therapeutic antibodies targeting the V-set domain or sialic acid-binding domain of CD33 have been approved to treat acute myeloid leukemia.^39^ Also, in the context of hepatitis B virus (HBV) infection, the use of anti-CD33 was able to reverse hHBV-mediated immunosuppression in pre-clinical *in vitro* studies.^40^ Finally, a recent study in Alzheimer’s Disease has ranked lintuzumab (SGN-33) as one of the top repurposed drug candidates for treating AD.^41^ Given this evidence, it is tempting to propose that anti-CD33 mAb could be employed in HIV-1 infection, with a potential dual beneficial effects of reducing HIV-1 replication and improving HIV-1-associated neurocognitive deficits.

To validate our observations in the small number of BCN02 participants, a large cohort of ART naïve, chronically HIV-1+ individuals with a wide range of viral loads was analyzed. These results confirmed that HIV-1 infected individuals with poor virus control (HIV-high) had elevated plasma levels of CD33, which were also positively correlated with both, viral load and proviral DNA copy numbers. This is in line with a report by Rempel et al., showing that several other members of the Siglec family can facilitate infection and viral replication (Siglec-5, -9, and as shown above, Siglec-7).^42^^,^^43^ It has also been well established that the direct interaction between sialylactose-containing gangliosides in the HIV viral membrane and the cellular lectin Siglec-1 is critical for HIV-1 capture and storage by mature dendritic cells and for trans-infection between infected dendritic cells and T cells.^43^^,^^44^ In addition, the HIV-1 gp120 surface protein has been shown to serve as viral ligand for Siglec-3,^34^ which is thought to facilitate infection of macrophages and T cells.^33^^,^^45^

Finally, based on our observation that plasma CD33 predicts post-treatment control in BCN02 and the described physical associations between CD33/Siglec-3 and HIV-1 particles, we explored the possibility to inhibit HIV-1 replication by targeting CD33 in *in vitro* cultures using an anti-CD33 mAb. Indeed, in T cells and MDMs viral replication was strongly suppressed in a dose-dependent manner. While the effects of such treatment on acute infection of CNS cells needs to be further explored, these therapeutic CD33 targeting antibodies have been used for Hepatitis B virus, AML treatment and Alzheimer Disease,^39^, ^40^, ^41^ moving them closer to be tested in a clinical setting in HIV infection and with more extensive CNS assessment than what was possible in the BCN02 trial.

The present study has a number of limitations, aside from the small size of the BCN02 clinical trial. In particular, larger studies that include placebo controls will be needed to confirm the ability of CD33 levels to predict virus control in a treatment interruption phase either assessed prior to treatment initiation or at ART cessation. Of note, such studies should also include female participants, as the present analyses were based largely on male study participants. Sample availability for the current BCN02 sub-study was limited, and it did not include tissue samples such as lymph nodes, MALT and/or CSF, preventing us from conducting further tissue-based analyses that could have helped to especially further validating the CNS findings. Despite these limitations though, our analyses document that the “kick and kill” strategy used in BCN02 left specific marks on inflammatory and neurological plasma proteomes and allowed us to identify soluble CD33 as a novel biomarker of poor virus control (both, during the ART interruption phase of BCN02 and in natural HIV infection). Our studies fall short of defining the precise mechanism of how soluble and/or cellular CD33 may influence viral loads, although similarities to the mechanism of action of other Siglec family members (i.e., Siglec-7) lend importance to the soluble form of CD33. The fact that the CD33 plasma levels were elevated already in pre-intervention samples in line with this and suggest that the CD33 protein is critically involved in viral replication in vivo and that it could be therapeutically targeted in future cure approaches.

## Contributors

MRR and CB designed the experimental plan. BM, TH, and JM contributed to the generation of clinical trial samples, participant data and sample management. AP and JAMM performed neuropsychological testing. BOT, CDC, and ALL performed the sample processing. JMP and CG determined viral parameters. CG, JS contributed in the recruitment of untreated HIV-1+ individuals in Lima, Peru. CDC and MRR analyzed plasma proteomics data and integrated them with clinical and virological parameters. CDC performed RT-PCR and conducted the *in vitro* experiments. CDC and BOT run statistical, bioinformatics and integrative analyses using R program. EB and EGV helped with monocyte-derived macrophages (MDMs) infection and proviral PCR quantification procedures. CDC, CB, and MRR drafted and edited the manuscript. CB and BC procured resources and funding for the execution of the study. All authors reviewed and approved the final version of the manuscript.

## Data sharing statement

Additional information can be found in the Supplementary Material of this article.

## Declaration of interests

BM is a consultant of AELIX THERAPEUTICS, SL outside the submitted work. CB is co-founder, chief science officer and shareholder of AELIX THERAPEUTICS. TH is a coinventor of the HIVconsv immunogen. All other authors declare that they have no competing interests.

## Appendix A Supplementary data

Supplementary Tables S1–S4

Protocol BCN02-Romi v4
