# Supplementary material for: Plasma proteomic profiling identifies CD33 as a marker of HIV control in natural infection and after therapeutic vaccination
Source: eBioMedicine. 2023 Jul 26;95:104732. doi: 10.1016/j.ebiom.2023.104732 (PMC10410179; doi:10.1016/j.ebiom.2023.104732)
Supplement: Protocol BCN02-Romi v4 [file mmc2.pdf]

AN OPEN LABEL PHASE I TRIAL TO EVALUATE THE SAFETY AND EFFECT OF HIVconsv VACCINES IN COMBINATION WITH HISTONE DEACETYLASE INHIBITOR ROMIDEPSIN ON THE VIRAL REBOUND KINETIC AFTER TREATMENT INTERRUPTION IN EARLY TREATED HIV-1 INFECTED INDIVIDUALS.

Code: BCN02-ROMI
RM-CL-OTHER-PI-005414

Version 4, 30th May 2016
(Amendment 1 and 2 included)

EudraCT: 2015-002300-84

ANNEX 3, OP.02.01.FLS-RS.02. Pre-estudi 14/10/2014

Sponsor:

IrsiCaixa AIDS Research Institute
Hospital Universitari Germans Trias i Pujol
Carretera de Canyet s/n
08916 Badalona (Barcelona)
Spain

Coordinating Investigator: Beatriz Mothe Pujadas, MD, PhD

Co-Investigator: José Moltó Marhuenda, MD, PhD

The information contained in this document is confidential and must not be revealed to third persons without prior authorization as contemplated by Law.

STUDY PHYSICIANS	Unitat Polivalent d'Investigació Clínica (UPIC)
**HGTIP, Badalona**	Dra. Ana María Barriocanal Barriocanal<br>T: +34 934 978 865 (ext 3710)<br>abarrio.germanstrias@gencat.cat
Dra. Beatriz Mothe Pujadas<br>T: +34 934 656 374 (Ext 165)<br>bmothe@irsicaixa.es	Dr. Magí Farré<br>T: +34 934 978 865 (ext 3710)<br>mfarre.germanstrias@gencat.cat
Dr. Jose Moltó Marhuenda<br>T: +34 934 978 887<br>jmoltó@flsida.org	**Projecte dels NOMS-HISPANOSIDA, BCN Checkpoint**
Dr. Pep Coll Verd<br>T: +34 934 656 374<br>pcoll@irsicaixa.es	Michael Meulbroek<br>T: +34 933 182 056<br>mmeulbroek@hispanosida.com
Dr. Bonaventura Clotet i Sala<br>T: +34 934 656 374<br>bclotet@irsicaixa.es	Dr. Pep Coll<br>T: +34 934 656 374<br>pcoll@irsicaixa.es
**HClínic, Barcelona**	**STUDY NURSES/DATA MANAGEMENT**
Dr. Christian Manzardo<br>T: +34 932 275 400<br>cmanzard@clinic.ub.es	Patricia Cobarsi/Cristina Miranda<br>Fundació Lluita contra la Sida, HGTIP, Badalona<br>T: +34 934 978 887<br>pcobarsi@flsida.org/cmiranda@flsida.org
Dr. Josep Maria Miró Meda<br>T: +34 932 275 400<br>jmmiro@clinic.ub.es	Carmen Ligero/Irene Ruiz Zamora<br>Infectious Diseases, Hospital Clínic, Barcelona<br>T: +34 932 275 400<br>irruiz@clinic.ub.es
Dr. David Nicolas<br>T: +34 932 275 400<br>dnicolas@clinic.ub.es	
Dr. Juan Ambrosioni<br>T: +34 932 275 400<br>ambrosioni@clinic.ub.es	
Dr. Josep Maria Gatell<br>T: +34 932 275 400<br>jmgatell@clinic.ub.es	

ANNEX 3, OP.02.01.FLS-RS.02. Pre-estudi 14/10/2014

ANNEX 3, OP.02.01.FLS-RS.02. Pre-estudi 14/10/2014

CELLULAR IMMUNITY	STATISTICS
Dra. Marta Marszalek/Miriam Rosas<br>Host Genetics and Cellular Immunity<br>IrsiCaixa AIDS Research Institute, HGTIP, Badalona<br>T: +34 934 656 374<br>mmarszalek@irsicaixa.es/mrosas@irsicaixa.es<br><br>Dr. Christian Brander<br>Host Genetics and Cellular Immunity<br>IrsiCaixa AIDS Research Institute, HGTIP, Badalona<br>T: +34 934 656 374 (Ext 166)<br>cbrander@irsicaixa.es<br><br>**VIRAL RESERVOIR AND REACTIVATION**<br><br>Sara Morón/Dra. M.Carmen Puertas<br>Retrovirology and Clinical Studies<br>IrsiCaixa AIDS Research Institute, HGTIP, Badalona<br>T: +34 934 656 374<br>smoron@irsicaixa.es / mpuertas@irsicaixa.es<br><br>Dr. Javier Martinez-Picado<br>Retrovirology and Clínical Studies<br>IrsiCaixa AIDS Research Institute, HGTIP, Badalona<br>T: +34 934 656 374 (Ext 150)<br>jmpicado@irsicaixa.es<br><br>**PHARMACY, PHARMACOKINETICS AND PHARMACODYNAMICS**<br><br>Cristina Perez<br>Pharmacy Service, HGTIP, Badalona<br>T: +34 934 978 874<br>cperez@igtp.cat<br><br>Dr. Jose Moltó Marhuenda<br>HIV Unit, HGTIP, Badalona<br>T: +34 934 978 887<br>jmolto@flsida.org<br><br>Dra. Marta Valle<br>Pharmacokinetic/pharmacodynamic modeling and simulation, Institut de Recerca HSCSP, Barcelona<br>T: +34 935 537 857<br>mvallec@santpau.cat	Dra. Núria Pérez Alvarez<br>Fundació Lluita contra la Sida, HGTIP, Badalona<br>Statistics and Operations Research Department<br>Technical University of Catalonia<br>T: +34 934 978 887<br>nperez@flsida.org<br><br>Dra. M. Luz Calle Rosingana<br>Bioinformatics and Medical Statistics.<br>Universitat de Vic - Universitat Central de Catalunya<br>T: +34 938 816 179<br>malu.calle@uvic.cat<br><br>**COLLABORATING INVESTIGATORS (OXFORD UNIVERSITY)**<br><br>Dr. Lucy Dorrell<br>Nuffield Dept. Medicine (NDM)<br>University of Oxford<br>Old Road Campus Research Building<br>Roosevelt Drive Oxford OX3 7DQ, UK<br>lucy.dorrell@ndm.ox.ac.uk<br><br>Dr. Tomáš Hanke<br>The Jenner Institute<br>University of Oxford<br>Old Road Campus Research Building<br>Roosevelt Drive<br>Oxford OX3 7DQ, UK<br>tomas.hanke@ndm.ox.ac.uk<br><br>**STUDY MEDICATION PRODUCTION**<br><br>MVA.HIVconsv: IDT Biologika GmbH, Germany.<br>Romidepsin: Celgene Ltd, Switzerland

ACCONTING	MONITORING
Glòria Bigorra<br>Management<br>Fundació Institut d'Investigació en Ciències de la Salut Germans Trias i Pujol (IGTP), Badalona<br>T: +34 934 978 839<br>gbigorra@igtp.cat<br><br>Mireia Manent<br>Management<br>IrsiCaixa AIDS Research Institute, HGTIP, Badalona<br>T: +34 934 656 374 (Ext 113)<br>mmanent@irsicaixa.es<br><br>Albert Tuldrà<br>Management<br>Fundació Lluita contra la Sida, HGTIP, Badalona<br>T: +34 934 978 887 (Ext 3427)<br>atuldra@flsida.org	FLS-Research Support<br>Hospital Universitari Germans Trias i Pujol<br>T. +34 93 497 84 14<br><br>Roser Escrig<br>T. +34 629 77 36 20<br>rescrig@fls-rs.com<br><br>Sílvia Gel<br>T. +34 606 976 623<br>sgel@fls-rs.com<br><br>Jéssica Toro<br>T. +34 618 80 44 87<br>jtoro@fls-rs.com

ANNEX 3, OP.02.01.FLS-RS.02. Pre-estudi 14/10/2014

SIGNATURES

The coordinating investigator and the sponsor of the study:

AN OPEN LABEL PHASE I TRIAL TO EVALUATE THE SAFETY AND EFFECT OF HIVconsv VACCINES IN COMBINATION WITH HISTONE DEACETYLASE INHIBITOR ROMIDEPSIN ON THE VIRAL REBOUND KINETIC AFTER TREATMENT INTERRUPTION IN EARLY TREATED HIV-1 INFECTED INDIVIDUALS.

Declare that this study will be conducted in compliance with the protocol, Good Clinical Practices (GCP) and the applicable regulatory requirements.

Modifications to this protocol must be submitted prior agreement of the coordinator investigator and sponsor.

Coordinating Investigator: Beatriz Mothe Pujadas, MD, PhD

Signature and Date: 30/5/2016

Sponsor: Bonaventura Clotet Sala, PhD, MD
IrsiCaixa AIDS Research Institute

Signature and Date: 30/05/2016

ANNEX 3, OP.02.01.FLS-RS.02. Pre-estudi 14/10/2014

ANNEX 3, OP.02.01.FLS-RS.02. Pre-estudi 14/10/2014

1 GENERAL INFORMATION

1.1 TITLE

AN OPEN LABEL PHASE I TRIAL TO EVALUATE THE SAFETY AND EFFECT OF HIVconsv VACCINES IN COMBINATION WITH HISTONE DEACETYLASE INHIBITOR ROMIDEPSIN ON THE VIRAL REBOUND KINETIC AFTER TREATMENT INTERRUPTION IN EARLY TREATED HIV-1 INFECTED INDIVIDUALS.

1.2 CODE

BCN02-ROMI

1.3 PROTOCOL VERSION AND DATE

Version 4, 30th May 2016
Any modification of the protocol must also bear the amendment number and date.

1.4 SPONSOR

IrsiCaixa AIDS Research Institute
Hospital Universitari Germans Trias i Pujol
Carretera de Canyet s/n
08916 Badalona (Barcelona)
T. +34 93 465 63 74

Person authorized by the sponsor to sign the protocol and amendments:
Bonaventura Clotet Sala, MD, PhD

1.5 MONITOR

Roser Escrig Sarreta / Sílvia Gel Moretó / Jéssica Toro
FLS-Research Support
Carretera de Canyet, s/n
08916 – Badalona (Barcelona) SPAIN
Telephone: + 34 93 497 84 14
Fax: + 34 93 465 76 02
rescrig@fls-rs.com, M. +34 629 77 36 20
sgel@fls-rs.com, M. +34 606 97 66 23
jtoro@fls-rs.com, M. +34 618 804 487

1.6 COORDINATING INVESTIGATOR

Beatriz Mothe Pujadas, MD, PhD

1.7 SITES AND INVESTIGATORS

Study will be performed in:

- Hospital Universitari Germans Trias i Pujol, Badalona. Investigators will be:

Principal investigator: Beatriz Mothe Pujadas, MD, PhD
IrsiCaixa AIDS Research Institute
Hospital Universitari Germans Trias i Pujol
Carretera de Canyet s/n
08916 Badalona (Barcelona)
T. +34 93 465 63 74
E-mail: bmothe@irsicaixa.es

- Hospital Clínic de Barcelona

Principal investigator: Josep Maria Miró Meda, MD, PhD
Infectious Diseases Service Hospital Clínic Barcelona
Villarroel, 170, 08036 Barcelona
T: 93 227 54 00
E-mail: jmmiro@clinic.ub.es

1.8 TECHNICAL SERVICES INVOLVED

Biochemistry, hematology, quantitative HIV-1 RNA levels and CD4 counts will be all performed in General Lab (Labco) (Contact details, Dr. Javier Galindo, Departament de Suport, c/ Londres, 28, 08029 Barcelona, Tel. +34 93 363 60 00), except for:

- Pre-RMD_1 timepoint, biochemistry and hematology performed in Hospital Germans Trias i Pujol central laboratories.
- Screening and Phase 3 biochemistry, hematology, quantitative HIV-1 RNA levels and CD4 counts will be performed in the central laboratories of each participating hospital.

Qualitative HIV-1 RNA determinations (X-pert HIV-1 Qual) will be performed at BCN-Checkpoint community center (contact details Dr. Pep Coll and Michael Meulbroek, pcoll@irsicaixa.es, T. +34 934 656 374 and mmeulbroek@hispanosida.com, T. +34 933 182 056).

Romidepsin plasma levels will be performed at Echevarne laboratory (contact details, Maria Rovira Guerin, mail, mrovira@echevarne.com, T. +34 93 504 01 18).

All other procedures including immunomonitoring and viral reservoir analyses will be performed in the IrsiCaixa AIDS Research Institute (responsible of sample distribution Dr. Beatriz Mothe, bmothe@irsicaixa.es, T. +34 93 465 63 74).

Vaccinations and Romidepsin infusion from all patients will be performed at Unitat Polivalent d'Investigació Clínica (UPIC), Phase I unit located at Hospital Universitari Germans Trias i Pujol. Responsible person: Dr. Magí Farré; Contact person: Dr. Ana María Barriocanal, abarrio.germanstrias@gencat.cat, T. +34 93 497 84 88.

ANNEX 3, OP.02.01.FLS-RS.02. Pre-estudi 14/10/2014

In vaccination and administration of romidepsin visits, patients will be offered a free shuttle service to UPIC through the company Bonotaxi (contact person: María Sánchez, T. +34 630 036 654, maria.sanchez@bonotaxi.com).

Electronic Capture Data Form (eCRF) will be developed by Fundació Institut Català de Farmacologia, Pg. Vall d'Hebron, 119 – 129, 08035, Barcelona (contact person: Xavier Barroso, T. +34 93 428 30 29 / +34 93 428 31 76, informatica@icf.uab.cat).

ANNEX 3, OP.02.01.FLS-RS.02. Pre-estudi 14/10/2014

ANNEX 3, OP.02.01.FLS-RS.02. Pre-estudi 14/10/2014

CONTENTS

SIGNATURES **5**
1 GENERAL INFORMATION **6**
1.1 TITLE *6*
1.2 CODE *6*
1.3 PROTOCOL VERSION AND DATE *6*
1.4 SPONSOR *6*
1.5 MONITOR *6*
1.6 COORDINATING INVESTIGATOR *6*
1.7 SITES AND INVESTIGATORS *6*
1.8 TECHNICAL SERVICES INVOLVED *7*
CONTENTS **9**
2 ABBREVIATIONS **13**
3 BACKGROUND INFORMATION **15**
3.1 HYPOTHESIS *20*
3.2 REFERENCES *21*
4 TRIAL OBJECTIVE AND PURPOSE **24**
4.1 PRIMARY OBJECTIVE *24*
4.2 SECONDARY OBJECTIVES *24*
5 TRIAL DESIGN **24**
5.1 TYPE OF TRIAL *24*
5.2 DESCRIPTION OF THE DESIGN *24*
5.3 ENDPOINTS *25*
5.3.1 Primary endpoint(s) 25
5.3.2 Secondary endpoints 25
5.4 MEASURES TO AVOID BIAS *27*
5.4.1 Randomization 27
5.4.2 Stratification 27
5.4.3 Blinding 27
5.5 FORESEEN CALENDAR *27*
5.6 END OF TRIAL *27*
5.6.1 Early study suspension 27
5.7 SOURCE DATA *28*
6 TRIAL INVESTIGATIONAL PRODUCT(S) **29**
6.1 EXPERIMENTAL TREATMENT *29*
6.2 SUPPLY, PACKAGING, LABELING AND STORAGE *29*
6.2.1 Romidepsin (RMD, Istodax®) 29
6.2.2 MVA.HIVconsv 29
6.3 DOSE, INTERVAL, ROUTE AND METHOD OF ADMINISTRATION *30*
6.3.1 Romidepsin (RMD, Istodax®) 30
6.3.2 MVA.HIVconsv 30
6.4 EXPERIMENTAL DRUG ACCOUNTABILITY *31*
6.5 ARM DESCRIPTION *31*
6.6 CONCOMITANT TREATMENTS *31*

ANNEX 3, OP.02.01.FLS-RS.02. Pre-estudi 14/10/2014

6.6.1 Ondansetron 31
6.6.2 cART 32
6.6.3 Other 32
6.6.4 MODIFICATION OF THE ANTIRETROVIRAL TREATMENT REGIMEN 32
6.7 COMPLIANCE 32

7 SELECTION AND WITHDRAWAL OF SUBJECTS 33
7.1 INCLUSION CRITERIA 33
7.2 EXCLUSION CRITERIA 33
7.3 SUBJECT WITHDRAWAL CRITERIA 34
7.3.1 Related to MVA.HIVconsv vaccination 34
7.3.2 Related to romidepsin administration 35
7.3.3 Related to cART interruption 37
7.3.4 Early subject withdrawal 37
7.3.5 Medical approach to withdrawal 38
7.3.6 Follow-up after early withdrawal 38
7.3.7 Replacement of patients 38
7.4 PRE-RANDOMIZATION / PRE-BASELINE LOSSES 38

8 TRIAL CONDUCTION AND RESPONSE EVALUATION 39
8.1 TRIAL DEVELOPMENT 39
8.1.1 SCR – Screening (week -1) 39
8.1.2 MVA1 – First MVA vaccination (week 0) 39
8.1.3 MVA1+1w – One week post- MVA1 vaccination (week 1) 40
8.1.4 RMD1 – First romidepsin infusion (week 3) 40
8.1.5 RMD1 + 3d – 3 days post RMD1 infusion (week 3+ 72h) 41
8.1.6 RMD2 – Second romidepsin infusion and MVA1+4w – Four weeks post MVA1 vaccination (week 4) 41
8.1.7 RMD2 + 3d – 3 days post RMD2 infusion (week 4+72h) 42
8.1.8 RMD3 – Third romidepsin infusion (week 5) 42
8.1.9 RMD3 + 3d – 3 days post RMD3 infusion (week 5 +72h) 43
8.1.10 RMD3 + 1w – One week post RMD3 infusion (week 6) 43
8.1.11 MVA2 – Second MVA vaccination and MVA1+9w – Nine weeks post MVA1 vaccination (week 9) 44
8.1.12 MVA2+1w – One week post- MVA2 vaccination (week 10) 44
8.1.13 MVA2+4w – Four weeks post- MVA2 vaccination (13 weeks) 44
8.1.14 MAP – Monitored antiretroviral pause and MVA2+8w – Eight weeks post MVA2 vaccination (17 weeks) 45
8.1.15 MAP phase monitoring 46
8.1.16 cART resumption visit (7-10 days after first Qual pVL positive) 48
8.2 CART+4W, CART+12W – CART FOLLOW-UP VISITS (4 AND 12 WEEKS AFTER CART RESUMPTION) 49
8.2.1 cART+24w – end of trial visit (24 weeks after cART resumption) 50
8.3 LONG-TERM FOLLOW-UP 50
8.4 PROCEDURES FOR EVALUATION OF RESPONSE 50
8.4.1 Clinical record and physical exam 50
8.4.2 Laboratory tests 51
8.4.3 Electrocardiogram 52
8.4.4 Vaccine patient diary 52
8.4.5 Romidepsin patient diary 53

9 ADVERSE EVENTS 55
9.1 DEFINITION 55
9.2 MONITORING, RECORDING AND REPORTING OF ADVERSE EVENTS 55
9.3 DOCUMENTATION RELATED TO AE AND SAE 56
9.4 EVALUATION OF ADVERSE EVENTS 56
9.4.1 Seriousness 56
9.4.2 Severity / Intensity 58
9.4.3 Causality 58
9.4.4 Duration 59
9.4.5 Action Taken 59
9.4.6 Outcome 59
9.5 ABNORMAL LABORATORY VALUES 60
9.6 FEMALES OF CHILDBEARING POTENTIAL 60
9.6.1 Male Subjects 61
9.7 SCIENTIFIC COMMITTEE 61
9.8 EXPEDITED REPORTING OF ADVERSE EVENTS 61
9.8.1 Reporting to Regulatory Authorities and the Ethics Committee 61
9.8.2 Immediate reporting by Investigator to Sponsor 62
9.8.3 Reporting by Sponsor to Celgene 62
9.8.4 Reporting by Sponsor to Coordinating investigator 62

10 STATISTICS 63
10.1 RESPONSE EVALUATION (EFFICACY) 63
10.2 SECURITY ANALYSIS 64
10.3 SAMPLE SIZE DESCRIPTION 65
10.4 DEVIATION OF STATISTICAL PLAN 65

11 DIRECT ACCESS TO SOURCE DATA/DOCUMENTS 66

12 QUALITY CONTROL AND QUALITY ASSURANCE 67
12.1 STUDY MONITORING 67
12.2 AUDITS AND INSPECTIONS 67
12.3 CASE REPORT FORM 68

13 ETHICS 69
13.1 GENERAL CONSIDERATIONS 69
13.2 PATIENT INFORMATION SHEET AND INFORMED CONSENT 69
13.3 PAYMENT TO RESEARCH SUBJECTS 69

14 DATA HANDLING AND RECORD KEEPING 70
14.1 DATA HANDLING 70
14.2 RECORD KEEPING 70
14.2.1 Investigator file and document retention 70
14.2.2 Source documents and basic data 70

15 FINANCING AND INSURANCE 72
15.1 SOURCE OF FINANCING 72
15.2 INSURANCE POLICY 72

16 PUBLICATION POLICY 73

ATTACHMENT A: STUDY DIAGRAM 74

ATTACHMENT B: ROMIDEPSIN ADMINISTRATION FORM 76

ANNEX 3, OP.02.01.FLS-RS.02. Pre-estudi 14/10/2014

ATTACHMENT C: MVA.HIVCONSV ADMINISTRATION FORM 76
ATTACHMENT D: ADAPTED SERAD QUESTIONAIRE 76
ATTACHMENT E: MAP PHASE MONITORING FORM 77
ATTACHMENT F: MVA DIARY CARD 77
ATTACHMENT G: ROMIDEPSIN DIARY CARD 77
ATTACHMENT H: SOP FOR ROMIDEPSIN HANDLING AND ADMINISTRATION 77
ATTACHMENT I: SOP FOR MVA.HIVCONSV HANDLING AND ADMINISTRATION 77
ATTACHMENT J: SCHEDULE OF PROCEDURES 77
ATTACHMENT K: SCHEME FOR PBMC/PLASMA STORAGE 77
ATTACHMENT L: MVA.HIVCONSV PRESCRIPTION FORM 77
ATTACHMENT M: MAP DECISIONS CHART 77
ATTACHMENT N: SAE/PREGNANCY NOTIFICATION INSTRUCTIONS AND FORM 77
ATTACHMENT O: DIVISION OF AIDS TABLE FOR GRADING THE SEVERITY OF ADULT AND PEDIATRIC ADVERSE EVENTS 78
ATTACHMENT P: SCIENTIFIC COMMITTEE 78
ATTACHMENT Q: CRF CONTENT 78
ATTACHMENT R: PATIENT INFORMATION AND WRITTEN INFORMED CONSENT 78
ATTACHMENT S: INSURANCE 78
ATTACHMENT T: ROMIDEPSIN INVESTIGATOR BROCHURE 78
ATTACHMENT U: MVA.HIVCONSV INVESTIGATOR BROCHURE 78
ATTACHMENT V: STUDY STAFF 78
ATTACHMENT W: PK FORM 78
ATTACHMENT X: 'INDICATION FOR MAP PHASE' FORM 78
ATTACHMENT Y: ONDANSETRON SUMMARY OF PRODUCT CHARACTERISTICS 79
ATTACHMENT Z: SUBSTUDY BCN02-MICROBIOME PROTOCOL 79
ATTACHMENT AA: SUBSTUDY BCN02-NEURO PROTOCOL 79
ATTACHMENT AB: cART INTERRUPTION DECISION CHART 79

ANNEX 3, OP.02.01.FLS-RS.02. Pre-estudi 14/10/2014

ANNEX 3, OP.02.01.FLS-RS.02. Pre-estudi 14/10/2014

2 ABBREVIATIONS

AE	Adverse event
AEMPS	Agencia Española de Medicamentos y Productos Sanitarios
AIDS	Acquired immune deficiency syndrome
ALT	Alanine aminotransferase
AR	Adverse reaction
BL2	Biological Level 2
BP	blood pressure
CAC	Community Advisory Committee
cART	Combination Antiretroviral Therapy
ChAd	Attenuated chimpanzee adenovirus
CRF	Case Report Form
CTA	Clinical Trial Agreement
CTL	Cytotoxic T Lymphocyte
DSMB	Data Safety Monitoring Board
EC	Ethics Committee
eCRF	Electronic CRF
ELISPOT	Enzyme-linked immunospot
GCP	Good Clinical Practice
GP	General Practitioner
HAART	Highly active antiretroviral therapy
HBsAg	Hepatitis B virus surface antigen
HBV	Hepatitis B virus
HC	Clínic Hospital
HCV	Hepatitis C virus
HGTIP	Germans Trias i Pujol Hospital
HIV	Human immunodeficiency virus
HLA	Human Leukocyte Antigen
IB	Investigator's Brochure
ICF	Informed Consent Form

ICH	International Conference of Harmonisation
IFN-γ	Interferon gamma
IMP	Investigational Medicinal Product
IRB/IEC	Institutional Review Board/Independent Ethic Comitte
iv	Intravenous
LC-MS	Liquid chromatography–mass spectrometry
LRA	Latency Reversal Agent
MS	Mass spectrometry
MVA	Modified vaccinia virus Ankara
NRTI	Nucleoside Reverse Transcriptase Inhibitor
PBMCs	Peripheral blood mononuclear cell
PBS	Phosphate buffered saline
PCR	Polymerase chain reaction
PI	Principal Investigator
PIS	Participant/ Patient Information Sheet
pVL	Plasma viral load
RMD	Romidepsin
SAE	Serious Adverse Event
SAR	Serious Adverse Reaction
SUSAR	Suspected Unexpected Serious Adverse Reaction
TMF	Trial Master File
TPHA	Treponema Pallidum Haemagglutination
UNAIDS	Joint United Nations Programme on HIV/AIDS

ANNEX 3, OP.02.01.FLS-RS.02. Pre-estudi 14/10/2014

ANNEX 3, OP.02.01.FLS-RS.02. Pre-estudi 14/10/2014

3 BACKGROUND INFORMATION

The generalization of highly active combination antiretroviral therapy (cART) has resulted in a dramatic decrease in morbidity and mortality associated to infection by the human immunodeficiency virus (HIV). However, despite having new families of antiretroviral drugs, the current cART is not able to eradicate HIV from the body. Consequently, cART cessation in patients maintaining undetectable viral load is followed by a fast rebound in viremia[1,2]. This reflects the inability of the standard cART in eliminating a viral reservoir formed by latently infected cells in which the integrated provirus remains quiescent and very stable since very early stages of infection, and the inability of the immune response to effectively contain viral rebound after treatment interruption.

Multiple strategies have been evaluated to try to achieve an optimal control of HIV infection in the absence of cART. These have included early treatment initiation within the first 6 months after HIV acquisition, cART intensification, immunotherapies including interleukin administrations (IL-2, IL-7, IL-10, IL-12, IL15 ...), treatment with cyclosporine, mycophenolate, hydroxyurea, thalidomide, passive administration of antibodies, etc. and a wide range of therapeutic vaccines designed to expand the response mediated by cytotoxic T lymphocytes[3-6]. The largest clinical effect demonstrated to date by a vaccination strategy was obtained with an autologous dendritic-cell vaccination approach, which was able to demonstrate 1 log reduction in the viral setpoint after discontinuation of treatment[7] and which is certainly suboptimal.

To date, the most relevant cause of therapeutic vaccines' failure probably lies in the composition of the antigen insert (immunogen) expressed. In particular, the inclusion of whole HIV proteins as antigens limits the immunogenic effect of the vaccine towards a unspecific CTL expansion; a CTL response pattern which, in natural HIV infection, has been proved ineffective in controlling viral replication in most individuals[8,9]. In this regard, there is a growing consensus in the need to improve the immunogen design by selecting viral sequences able to induce T cell responses which are more beneficial to the host. Our group has contributed significantly to the identification of those viral targets and participated, along with other groups, in the design and testing of new concepts of immunogens, in which the HIVconsv vaccines are in the most advanced stage of development[10-12].

Another important limitation of therapeutic vaccines has been its likely no / little impact of the induced cytotoxic response on the viral reservoir. This is important because the size of the reservoir is one of the few factors that has been associated with the time to viral rebound after treatment interruption[13,14] (Frater communications in CROI 2015 and Rosas, Keystone 2015). However, recent clinical examples suggest that undetectable viral reservoir (assessed using the most sensitive techniques available) does not predict absence of viral rebound and immune control following treatment interruption[15,16]; therefore, better immune correlates of control are urgently needed

Various strategies have been proposed to reduce the size of the reservoir by disrupting the viral latency and thus induce virus expression and cell death[17]. This has resulted in an increasing interest in a family of drugs, histone deacetylation inhibitors (HDACi) among others. In the context of HIV infection, the highest degree of histones' acetylation

results in a greater degree of gene transcription which, in patients with HIV infection, may result in reactivation of the virus and viral protein expression by latently infected cells[18-20]. Such in-vitro observations have led to the implementation, in a very short period of time, of several clinical trials using drugs such as vorinostat (SAHA) and panobinostat[21] and other non-HDACi agents such as disulfiram[22] and lithium[23] . In these trials, short and variable degrees of viral reactivation have been demonstrated but without significant reductions of the reservoir size [24].

In an in-vitro model of latency, R. Siliciano's group later suggested that reducing the viral reservoir could also require a stimulated cytotoxic T cell response capable of eliminating cells in which the virus was reactivated[25]. 'Kick and kill' strategies were then postulated necessary for a functional cure, where latency reversal agents (LRA) should be administered in combination with therapeutic T cell vaccines. The main player in such boosted immunity is believed to be the HIV-1-specific CD8+ T cell response. Such virus-specific CD8 T cells have been shown in acute infection to efficiently suppress viral replication, whereas CD8+ T cell responses present in the chronic phase of infection appear often functionally impaired. In addition to functional characteristics, the fine specificity of CD8 T cell responses have been shown to have a dominant effect on viral control in vivo; an observation that is essential for therapeutic HIV vaccine design.

To date, only 3 clinical trials worldwide addressing this hypothesis have been developed or are planned. The RISVAC03 trial (in which our group participated), therapeutic vaccine MVA-B was administered in combination with disulfiram [26] . The strategy did not show any effect in the control of viral rebound after cessation of treatment, probably because the vaccine was poorly immunogenic and disulfiram had a limited capacity on viral reactivation at the given doses[22]. The REDUC trial (NCT02092116), which is still ongoing, will assess the administration of romidepsin in combination with the therapeutic vaccine Vacc-4x (results are expected in late 2015) in chronic HIV infected individuals. In this trial, romidepsin has already been shown to be safe and with a detectable effect on transcription of viral RNA in 6 individuals that did not receive vaccination (safety arm)[27]. However, and based on the immunogenicity reported in previous studies with Vacc-4x[28], we hypothesized that the combined strategy will be also insufficient to contain viral rebound after treatment interruption. Lastly, the RIVER study (NCT02336074), which recruitment is expected to start in September 2015 is assessing the impact on the viral reservoir of HIVconsv vaccines in combination with SAHA in individuals early treated with intensified cART (4 drugs). The main limitation of this study potentially lies in the lower effect on the reactivation caused by SAHA compared with that of romidepsin[20] and no contemplation of an interruption phase of treatment to assess the effect of the combined strategy on viral rebound containment.

The BCN02-Romi study aims to evaluate a combined "kick and kill" strategy using the most immunogenic candidate vaccine available so far (HIVconsv) with the strongest LRA available at present time (romidepsin) in a cohort of early-treated HIV positive individuals.

HIVconsv vaccines

Our group has participated in significant studies on improvement of T cell immunogen designs aiming to redirect HIV-specific CTL response to some of the most conserved

ANNEX 3, OP.02.01.FLS-RS.02. Pre-estudi 14/10/2014

regions of the virus that have a role in the effective control of *in vivo* viral replication[12,29] and that could also limit the ability of the virus to escape[10,11,30]. ChAdV63.HIVcons vaccine candidates and MVA.HIVconsv[10] have been designed at University of Oxford, particularly to fight viral diversity of HIV-1. Importantly, ChAdV63.HIVconsv delivers the immunogen through a viral vector derived from chimpanzee adenovirus 63, for which humans do not have pre-existing antibodies that could hamper vaccine responsiveness nor have a deleterious effect as it has been suggested for other preventive vaccines using the Ad5[31,32]. Relevantly, at present Chimpanzee adenoviruses are being explored also as vaccine vectors for ebola[33]. MVA.HIVconsv uses the modified vaccinia virus Ankara as a non-replicating expression vector. Its immunogenicity profile, particularly as an enhancer in induction strategies and heterologous booster (prime-boost) has been extensively characterized. Both vaccines were tested in combination in HIV-1 negative individuals (HIV-CORE002 trial, NCT01151319) in UK, demonstrating safety and a frequency of specific T cells induced to conserved areas of the virus up to 10 times[34, 35] than the frequency observed by other vaccine candidates tested in large studies such as STEP[36] or RV144[37] trials. In early-treated individuals, data from BCN01 trial (NCT01712425) confirms the safety profile of HIVconsv vaccines and shows comparable immunogenicity to that observed in HIV negative vonlunteers[33]. Importantly, BCN01 trial demonstrates the ability of HIVconsv vaccines to redirect the dominance of specifically cytotoxic response towards conserved regions of the virus that are subdominantly targeted in natural infection (Mothe et al, IAS 2015, #MOPEA036). Viral reservoir determinations 1 year after treatment initiation were detectable in all individuals and were comparable to early-treated individuals who did not receive any vaccination.

ANNEX 3, OP.02.01.FLS-RS.02. Pre-estudi 14/10/2014

Romidepsin (RMD, Istodax®) is a potent HDACi approved in the US for the treatment of cutaneous T-cell lymphoma (CTCL) in patients who have received at least one prior systemic therapy and peripheral T-cell lymphoma (PTCL) in patients who have received at least one prior systemic theraphy whose marketing-authorization holder in Europe is Celgene Ltd. In Spain, RMD has only been used in clinical trials on hematology/oncology field under the investigation new drug application (PEI 07047). In the HIV field, RMD has been proposed as a potent HIV LRA. The first *in-vitro* results showed that RMD was able to induce HIV expression in latently infected CD4 cells from chronically suppressed HIV positive individuals[19,20]. More recently, Sogaard et al (IAS 2014 TUAA0106LB) reported the first *in-vivo* results of detectable HIV reactivation in 6 individuals receiving a cycle of 3 doses of RMD (5mg/m^2 iv, one third of the dose used in hematology and equivalent to that intended to be administered in the present trial) without safety concerns. Jones et al. published in 2014 the results of a complementary *in-vitro* study showing that HDACi could have a negative impact on the functionality of the cytotoxic T lymphocytes[38]. However, the clinical translation of this effect *in vivo* is still unknown; it may be only transiently relevant in activated cell populations or, on the contrary, be limited to a reversible temporary reduction of nonspecific cytotoxic capacity. In addition, knowledge on the possible link between RMD exposure and its reactivation capacity in this vaccination scenario is elusive.

Clinical context:

The BCN01 trial (NCT01612425) held in Barcelona in 2014 evaluated the safety and immunogenicity of ChAd.HIVconsv and MVA.HIVconsv vaccines in a cohort of 24

ANNEX 3, OP.02.01.FLS-RS.02. Pre-estudi 14/10/2014

individuals with documented acute / recent HIV-1 infection (confirmed to be <6 months from HIV transmission) 6 months after cART initiation with Truvada®/Isentress®. Early treatment of these patients was hypothesized to lead to: a) reduced progression to immune escape of viral sequences stored in the viral reservoir[29], b) limitation on the size of the viral reservoir[39] and c) lower immune depletion and immunoactivation which could result in an increased immune responsiveness to vaccination strategies[40] as shown by immunogenicity preliminary data.

Vaccinations were all safe and well tolerated. BCN01 participants are currently in an extension phase of the trial (BCN01-RO) keeping high retention rates and without cART discontinuations/suboptimal adherence difficulties being recorded. All these features make these individuals the ideal study population to evaluate the effectiveness of a strategy of "kick and kill" through a monitored treatment interruption. First, because CTL response could be boosted by a single second MVA.HIVconsv without experiencing new adverse effects unforeseen for the participants (e.g. injection site reactions,…). Second, as a plateau phase of reservoir decay is expected after 3 years of maintained viral suppression under cART, the impact of RMD on viral reservoir will be assessable a more conclusive longitudinally system, pre and post-RMD administration, after cART initation-mediated decay phase.

Population PK/PD analysis

To date, many aspects that can intervene on the effectiveness of a "kick and kill" strategy are still unknown. This project aims to incorporate a population pharmacokinetic/pharmacodynamic (PK/PD) analysis during RMD administration. The objective is to include: i) the development of a population pharmacokinetic model describing RMD pharmacokinetics in a population of early-cART suppressed HIV-infected individuals; ii) an *in-vivo* analysis of the magnitude and temporal effect of RMD either in HIV reactivation and CTL functional activity, an; iii) an evaluation of the relationship between RMD exposure and each of its effects.

Population PK/PD analysis may be a valuable methodology to achieve the above objectives. This strategy has proven to be a very useful tool in achieving the primary goal of therapy: predict individually, for each patient, the dose needed to achieve drug concentrations that maximize the therapeutic effects concerning its adverse reactions. With the PK/PD analysis it is related, on one hand, the administered dose with the concentrations of the drug, and on the other, the exposure to the drug with the effect that it is triggering, may it be therapeutical or unwanted. So, finally it is obtained the relationship between the dose of the drug and its effect (either therapeutic or unwanted)[41,42].

The fact of applying a population PK modeling approach makes possible to analyze all the drug concentration data simultaneously but keeping their individual origin, regardless of the dose received by each patient, their characteristics, and even if there is one or more concentration measurements in each patient. Thus, population PK/PD analysis provides information of both the typical trends (population mean) and the individual profiles of the drug concentration, and the time course of the effect[43,44]. In addition, this approach allows us to differentiate and quantify the variability between

different individuals (interindividual variability), the variability within the same individual when taking the drug on more than one occasion (intraindividual variability) and the so-called residual variability or not explained by the model, both in kinetic (drug concentrations) and dynamic processes (pharmacological effect). Finally, the population PK/PD analysis provides the ability to identify and quantify the effect that different individual characteristics (genetic, demographic, clinics ...) may have on each of the parameters describing these processes[45-47], permitting us to individualize the dose of the drug according to individual characteristics.

In this study, the information obtained will be of great clinical utility. Knowing those variables that may influence RMD pharmacokinetics and pharmacodynamics, will help to better define a therapeutic range of RMD to trigger HIV latency reactivation to be combined with therapeutic vaccines, and thus, identify more precisely the RMD dose that each patient should receive in order to maintain drug exposure within the desired range.

ANNEX 3, OP.02.01.FLS-RS.02. Pre-estudi 14/10/2014

Role of Treatment Interruption in the current HIV cure research agenda:
At the moment no reliable method has been validated to assess the predictive value of any in vitro test or parameter, one that could be benchmarked against a clinically relevant outcome for functional cure. Once a research strategy has proven safe and encouraging, allowing participants to interrupt their antiretroviral treatment remain the most reliable measure of viral persistence and will always be the final endpoint of strategies aimed at the cure of HIV infection. In 2014, at the HIV Cure IAS Symposium, Analytical Treatment Interruptions (ATI) which allowed a viral set point to be achieved after a usually fixed period of 16 weeks were recommended to be substituted for Monitored Antiretroviral Pause (MAP) strategies[48]. MAP strategies are considered safer to study participants. During a MAP cART is restarted when viral rebound is detected which could hamper potential fast viral reservoir replenishment. In such cases, the efficacy of the strategy is measured as the time to viral rebound in the context of interventions aimed at reducing the HIV reservoir.

To adapt the trial design according to our patient characteristics and thanks to excellent collaboration with BCN-checkpoint community center in Barcelona with dedicated clinical staff and novel laboratory technology already in place, weekly close monitoring during the MAP phase will be performed to allow rapid detection of viral rebound and ensure fast treatment resumption in case of needed.

Lastly, and to minimize risks of the intervention, an early interim and futility analyses is planned for each individual before the MAP phase. Treatment interruption would be discouraged individually if boosting MVA.HIVconsv was shown to be no longer immunogenic after romidepsin administration ((no net increase in induced response compared to pre-vaccination levels with MVA.HIVconsv at BCN02-Romi baseline visit, due to RMD toxicity on CTL functional activity for instance) as an improved viral control would not be foreseen. In such situation, trial results would be focused on the viral reservoir and immunogenicity endpoints only until better correlates of control would be available to predict clinically relevant outcomes on which one could recommend treatment interruptions in a safer manner.

ANNEX 3, OP.02.01.FLS-RS.02. Pre-estudi 14/10/2014

Correlates of Control:

As mentioned above, a better definition of immune correlates of viral control is urgently needed to selectively conduct future treatment interruptions in subpopulation of participants with highest chance of success after cessation of treatment and to minimize risks for participants.

If efficacy clinical outcomes during the MAP phase are achieved during the BCN02-Romi trial, study of correlates of control will be carefully performed to identify best predictors using multivariate regression and omic data integration techniques.

In summary, BCN02-Romi study is the first vaccine/eradication proof-of-concept clinical trial aiming to evaluate a combined "kick and kill" strategy using the most immunogenic candidate vaccines available so far (HIVconsv) with the strongest LRA available at present time (RMD). In addition, early treated HIV positive individuals poses an optimal target population to prove the effect of such a strategy in reducing the viral reservoir and containing viral rebound after a very well planned treatment interruption phase using a MAP strategy. Here, and through the development of a population PK/PD analysis, we will analyse the *in vivo* effects of RMD in the induction of HIV expression in resting cells, deeply investigate any unintended effect on the CTL function as well as predict the relationship between RMD exposure and such effects. Finally, our results will allow us to optimize RMD dosing, to evaluate the clinical efficacy of this strategy after the cART interruption and to identify better correlates of control of rebound viremia after cessation of treatment.

3.1 HYPOTHESIS

1. Administration of RMD (5 mg/m^2) to early-suppressed HIV infected individuals and whose CTL response has been refocused to more conserved regions of HIV by HIVconsv therapeutic vaccines will be safe and will reduce the viral reservoir.

2. Both HIV reactivation as well as, in case it is confirmed, eventual toxicity on CTL functionality by RMD, will be related to the exposure to RMD, allowing the definition of a RMD therapeutic range in this specific scenario.

3. After RMD administration, efficient re-stimulation of the cytotoxic response will be reached by MVA.HIVconsv, and the immunogenic response will be associated to the level of immune control after a monitored antiretroviral pause.

ANNEX 3, OP.02.01.FLS-RS.02. Pre-estudi 14/10/2014

3.2 REFERENCES

1. Moltó, J. et al. Influence of prior structured treatment interruptions on the length of time without antiretroviral treatment in chronically HIV-infected subjects. AIDS Res. Hum. Retroviruses 20, 1283–8 (2004).

2. El-Sadr, W. M. et al. CD4+ count-guided interruption of antiretroviral treatment. N. Engl. J. Med. 355, 2283–96 (2006).

3. Buzón, M. J. et al. HIV-1 replication and immune dynamics are affected by raltegravir intensification of HAART-suppressed subjects. Nat. Med. 16, 460–5 (2010).

4. Autran, B. et al. Greater viral rebound and reduced time to resume antiretroviral therapy after therapeutic immunization with the ALVAC-HIV vaccine (vCP1452). AIDS 22, 1313–22 (2008).

5. Schooley, R. T. et al. AIDS clinical trials group 5197: a placebo-controlled trial of immunization of HIV-1-infected persons with a replication-deficient adenovirus type 5 vaccine expressing the HIV-1 core protein. J. Infect. Dis. 202, 705–16 (2010).

6. Harrer, T. et al. Safety and immunogenicity of an adjuvanted protein therapeutic HIV-1 vaccine in subjects with HIV-1 infection: a randomised placebo-controlled study. Vaccine 32, 2657–65 (2014).

7. García, F. et al. A dendritic cell-based vaccine elicits T cell responses associated with control of HIV-1 replication. Sci. Transl. Med. 5, 166ra2 (2013).

8. Mothe, B. et al. Definition of the viral targets of protective HIV-1-specific T cell responses. J. Transl. Med. 9, 208 (2011).

9. Pereyra, F. et al. HIV Control Is Mediated in Part by CD8+ T-Cell Targeting of Specific Epitopes. J. Virol. 88, 12937–12948 (2014).

10. Létourneau, S. et al. Design and pre-clinical evaluation of a universal HIV-1 vaccine. PLoS One 2, e984 (2007).

11. Rolland, M., Nickle, D. C. & Mullins, J. I. HIV-1 group M conserved elements vaccine. PLoS Pathog. 3, e157 (2007).

12. Mothe, B. et al. A human immune data-informed vaccine concept elicits strong and broad T-cell specificities associated with HIV-1 control in mice and macaques. J. Transl. Med. 13, 60 (2015).

13. Gianella, S. et al. Effect of early antiretroviral therapy during primary HIV-1 infection on cell-associated HIV-1 DNA and plasma HIV-1 RNA. Antivir. Ther. 16, 535–45 (2011).

14. Sáez-Cirión, A. et al. Post-treatment HIV-1 controllers with a long-term virological remission after the interruption of early initiated antiretroviral therapy ANRS VISCONTI Study. PLoS Pathog. 9, e1003211 (2013).

15. Chun, T.-W. et al. Rebound of plasma viremia following cessation of antiretroviral therapy despite profoundly low levels of HIV reservoir: implications for eradication. AIDS 24, 2803–8 (2010).

16. Henrich, T. J. et al. Antiretroviral-free HIV-1 remission and viral rebound after allogeneic stem cell transplantation: report of 2 cases. Ann. Intern. Med. 161, 319–27 (2014).

17. Katlama, C. et al. Barriers to a cure for HIV: new ways to target and eradicate HIV-1 reservoirs. Lancet 381, 2109–17 (2013).

18. Archin, N. M. et al. Administration of vorinostat disrupts HIV-1 latency in patients on antiretroviral therapy. Nature 487, 482–5 (2012).

19. Wei, D. G. et al. Histone deacetylase inhibitor romidepsin induces HIV expression in CD4 T cells from patients on suppressive antiretroviral therapy at concentrations achieved by clinical dosing. PLoS Pathog. 10, e1004071 (2014).

ANNEX 3, OP.02.01.FLS-RS.02. Pre-estudi 14/10/2014

20. Rasmussen, T. A. et al. Comparison of HDAC inhibitors in clinical development: effect on HIV production in latently infected cells and T-cell activation. Hum. Vaccin. Immunother. 9, 993–1001 (2013).

21. Rasmussen, T. A. et al. Panobinostat, a histone deacetylase inhibitor, for latentvirus reactivation in HIV-infected patients on suppressive antiretroviral therapy: a phase 1/2, single group, clinical trial. Lancet HIV 1, e13–21 (2014).

22. Spivak, A. M. et al. A Pilot Study Assessing the Safety and Latency-Reversing Activity of Disulfiram in HIV-1-Infected Adults on Antiretroviral Therapy. Clin. Infect. Dis. 58, 883–90 (2014).

23. Puertas, M. C. et al. Effect of lithium on HIV-1 expression and proviral reservoir size in the CD4+ T cells of antiretroviral therapy suppressed patients. AIDS 28, 2157–9 (2014).

24. Bullen, C. K., Laird, G. M., Durand, C. M., Siliciano, J. D. & Siliciano, R. F. New ex vivo approaches distinguish effective and ineffective single agents for reversing HIV-1 latency in vivo. Nat. Med. 20, 425–9 (2014).

25. Shan, L. et al. Stimulation of HIV-1-specific cytolytic T lymphocytes facilitates elimination of latent viral reservoir after virus reactivation. Immunity 36, 491–501 (2012).

26. Mothe, B. et al. Safety and immunogenicity of a modified vaccinia Ankara-based HIV-1 vaccine (MVA-B) in HIV-1-infected patients alone or in combination with a drug to reactivate latent HIV-1. J. Antimicrob. Chemother. (2015). doi:10.1093/jac/dkv046

27. Søgaard OS1, et al. The Depsipeptide Romidepsin Reverses HIV-1 Latency In Vivo. PLoS Pathog. 2015 Sep 17;11(9):e1005142. doi: 10.1371/journal.ppat.1005142.

28. Pollard, R. B. et al. Safety and efficacy of the peptide-based therapeutic vaccine for HIV-1, Vacc-4x: a phase 2 randomised, double-blind, placebo-controlled trial. Lancet Infect. Dis. 14, 291–300 (2014).

29. Hancock, G. et al. Identification of effective subdominant anti-HIV-1 CD8+ T cells within entire post-infection and post-vaccination immune responses. PLoS Pathog. in press, (2015).

30. Deng, K. et al. Broad CTL response is required to clear latent HIV-1 due to dominance of escape mutations. Nature 517, 381–5 (2015).

31. Buchbinder, S. P. et al. Efficacy assessment of a cell-mediated immunity HIV-1 vaccine (the Step Study): a double-blind, randomised, placebo-controlled, test-of-concept trial. Lancet 372, 1881–93 (2008).

32. Gray, G., Buchbinder, S. & Duerr, A. Overview of STEP and Phambili trial Results: two phase IIb test of concept studies investigating the efficacy of MRK ad5 gag/pol/nef sub-type B HIV vaccine. Curr. Opin. HIV AIDS 5, 357–361 (2010).

33. Ledgerwood, J. E. et al. Chimpanzee Adenovirus Vector Ebola Vaccine — Preliminary Report. N. Engl. J. Med. 141126135947008 (2014). doi:10.1056/NEJMoa1410863

34. Borthwick, N. et al. Vaccine-elicited human T cells recognizing conserved protein regions inhibit HIV-1. Mol. Ther. 22, 464–75 (2014).

35. Hayton, E.-J. et al. Safety and tolerability of conserved region vaccines vectored by plasmid DNA, simian adenovirus and modified vaccinia virus ankara administered to human immunodeficiency virus type 1-uninfected adults in a randomized, single-blind phase I trial. PLoS One 9, e101591 (2014).

36. McElrath, M. J. et al. HIV-1 vaccine-induced immunity in the test-of-concept Step Study: a case-cohort analysis. Lancet 372, 1894–905 (2008).

37. Rerks-Ngarm, S. et al. Vaccination with ALVAC and AIDSVAX to prevent HIV-1 infection in Thailand. N. Engl. J. Med. 361, 2209–20 (2009).

38. Jones, R. B. et al. Histone deacetylase inhibitors impair the elimination of HIV-infected cells by cytotoxic T-lymphocytes. PLoS Pathog. 10, e1004287 (2014).

39. Hey-Cunningham, W. J. et al. Early antiretroviral therapy with raltegravir generates sustained reductions in HIV reservoirs but not lower T-cell activation levels. AIDS (2015). doi:10.1097/QAD.0000000000000625

40. Schuetz, A. et al. Initiation of ART during early acute HIV infection preserves mucosal Th17 function and reverses HIV-related immune activation. PLoS Pathog. 10, e1004543 (2014).

41. Whiting, B., Kelman, A. W. & Grevel, J. Population Pharmacokinetics. Clin. Pharmacokinet. 11, 387–401 (1986).

42. Meibohm, B. & Derendorf, H. Basic concepts of pharmacokinetic/pharmacodynamic (PK/PD) modelling. Int. J. Clin. Pharmacol. Ther. 35, 401–13 (1997).

43. Sheiner, L. B., Rosenberg, B. & Marathe, V. V. Estimation of population characteristics of pharmacokinetic parameters from routine clinical data. J. Pharmacokinet. Biopharm. 5, 445–79 (1977).

44. White, D. B., Walawander, C. A., Tung, Y. & Grasela, T. H. An evaluation of point and interval estimates in population pharmacokinetics using NONMEM analysis. J. Pharmacokinet. Biopharm. 19, 87–112 (1991).

45. Moltó, J. et al. Simultaneous population pharmacokinetic model for lopinavir and ritonavir in HIV-infected adults. Clin. Pharmacokinet. 47, 681–92 (2008).

46. Moltó, J. et al. Once- or twice-daily dosing of nevirapine in HIV-infected adults: a population pharmacokinetics approach. J. Antimicrob. Chemother. 62, 784–92 (2008).

47. Moltó, J. et al. Simultaneous pharmacogenetics-based population pharmacokinetic analysis of darunavir and ritonavir in HIV-infected patients. Clin. Pharmacokinet. 52, 543–53 (2013).

48. Anderson JL et al, Progress towards an HIV cure: update from the 2014 International AIDS Society Symposium. AIDS Res Hum Retroviruses. 2015 Jan;31(1):36-44. doi: 10.1089/AID.2014.0236.

ANNEX 3, OP.02.01.FLS-RS.02. Pre-estudi 14/10/2014

4 TRIAL OBJECTIVE AND PURPOSE

4.1 PRIMARY OBJECTIVE

To evaluate the safety and efficacy of RMD given in combination with a booster vaccination with MVA.HIVconsv for reduction of the viral reservoir in a cohort of early suppressed HIV-positive individuals who previously received an heterologous vaccination regimen with ChAd/MVA.HIVconsv in the BCN01 trial.

4.2 SECONDARY OBJECTIVES

1. To evaluate HIV reactivation capacity and effect of RMD on CTL functionality through an *ex vivo* model and to assess its *in vivo* translation
2. To perform a population PK/PD analysis to assess the association between HIV expression and CTL functionality effect *in vivo* with RMD exposure
3. To evaluate the immunogenicity of booster vaccinations with MVA.HIVconsv before and after RMD administration and the effect on viral rebound control after discontinuation of cART.

5 TRIAL DESIGN

5.1 TYPE OF TRIAL

Multicenter, open-label, single arm clinical trial.

5.2 DESCRIPTION OF THE DESIGN

See attachment A for study diagram.

All 24 early-suppressed HIV positive individuals enrolled in the extension phase of BCN01 trial and who meet all eligibility criteria will be invited to participate.

The trial will be divided in two phases with different primary endpoints. In the first part of the trial (**Part A: intervention phase**) RMD will be administered after a first booster vaccination with MVA.HIVconsv. The primary endpoint will be safety and efficacy of the combined intervention in the reduction of viral reservoir. Secondary endpoints will include the establishment of an *ex vivo* model to assess HIV reactivation and potential CTL functional impairment in the context of RMD and the development of a population PK/PD analysis to relate RMD effects with its exposure (see below endpoints 1 and 2 for details).

ANNEX 3, OP.02.01.FLS-RS.02. Pre-estudi 14/10/2014

In the second part of the trial (**Part B: MVA.HIVconsv plus MAP**) a second booster vaccination with MVA.HIVconsv will be given 4 weeks after last RMD infusion -to cover any potential impairment of previously induced CTL response due to RMD administration. After an interim futility analysis (w13), a MAP will be offered to all participants at week 17. Here, secondary endpoints will include all viral rebound efficacy clinical outcomes as well as the study of correlates of control (see below description of endpoints 3 for details)

5.3 ENDPOINTS

5.3.1 Primary endpoint(s)

1. Safety and tolerability evaluation as measured by ≥3 grade adverse events (AE) and serious adverse events (SAE).

2. Reduction of the viral reservoir measured in CD4+ T cells by digital droplet PCR (ddPCR) at baseline and 1 week after 3rd dose of RMD (total HIV-1 DNA copies per 10^6 CD4+ T cells)

5.3.2 Secondary endpoints

1. To evaluate *ex vivo* capacity of RMD to induce HIV expression in resting CD4+ T cells and to influence CTL functionality. Stored biological samples from the BCN01-RO phase will be used. Culture incubations with RMD will simulate Cmax concentrations of the clinical dose (5mg/m^2). Results of the *ex vivo* system will be associated with the frequency of detected HIVconsv-specific induced T cells and compared to the observations during the intervention phase to assess its *in vivo* translation. *Ex vivo* measurements will include:

   1.1. Cell-associated RNA (CA-RNA)
   1.2. Viability, necrosis and apoptosis levels by flow cytometry and using Annexin, 7AAD, CD3, CD4 and CD8 markers
   1.3. Activation, exhaustion and proliferation capacity
   1.4. IFNg production assessed by ELISPOT and after PBMC stimulation with HIVconsv-specific peptides, as well as other non-related pathogens, such as EBV and CMV.
   1.5. Evaluation of the in-vitro viral inhibition capacity in the presence of RMD to a panel of 3 viruses (CCR5 and CxCR4)

2. To perform a population PK/PD analysis to assess the relationship between HIV expression and CTL functionality effect *in vivo* with RMD exposure. Specific endpoints will include:

   2.1. RMD plasma concentrations at the end and 0.5, 1, 2, 4, 8, 10 and 20 hours after the 1st RMD infusion. RMD plasma concentrations at the end and 8

ANNEX 3, OP.02.01.FLS-RS.02. Pre-estudi 14/10/2014

hours after the 2nd and 3rd infusions. Plasma concentrations will be measured by LC-MS/MS, and will be used to develop a population PK model. Parameters will include: volumes of distribution, total and intercompartimental plasmatic clearance, maximum and minimum concentrations and area under the curve.

2.2. *In vivo* capacity of RMD to induce HIV expression in resting CD4+ T cells measured by CA-RNA and ultrasensitive plasma viral load (SCA) before RMD infusion and after 24 and 72h (RMD_1), 12 and 72h (RMD_2) and 12, 72 and 7 days (RMD_3)

2.3. *In vivo* levels of Histone H3 acetylation in lymphocytes before RMD infusion and after 24 and 72h (RMD_1), 12 and 72h (RMD_2) and 12, 72 and 7 days (RMD_3)

2.4. *In vivo* IFNg production assessed by ELISPOT and after PBMC stimulation with HIVconsv-specific peptides before RMD infusion and after 24 and 72h (RMD_1), 12 and 72h (RMD_2) and 12, 72 and 7 days (RMD_3)

2.5. *In vivo* CTL toxicity assessment based on the most relevant marker from Endpoint 1 (viability, activation, ex) before RMD infusion and after 24 and 72h (RMD_1), 12 and 72h (RMD_2) and 12, 72 and 7 days (RMD_3)

2.6. Development of a population PK/PD analysis to integrate pharmacokinetics of RMD and *in vivo* observed effects of RMD

3. To evaluate the immunogenicity of booster vaccinations with MVA.HIVconsv before and after RMD administration and the effect on viral rebound control after discontinuation of cART.

3.1. To evaluate immunogenicity of booster vaccinations, HIVconsv-specific T cell responses will be measured before, 1, 4 and 8 weeks after both booster vaccinations (MVA_1 and MVA_2) by IFNg ELISPOT using peptide pools covering different HIV proteins and HIVcons sequences. Total HIVconsv magnitude as well as HIVconsv focus over the rest of HIV-1 specific responses will be compared over time. These results will enable the futility analyses (see section 5.6)

3.2. Evaluation of the effect MVA.HIVconsv boost vaccinations on viral suppressive capacity of CD8+ T cells in vitro, using a flow cytometric assay 4 weeks after MVA_2.

3.3. Determine the proportion of individuals who initiate a MAP following the futility analysis

3.4. Determine the proportion of individuals who maintain sustained pVL <2,000 cpies/ml during the first 12weeks after treatment cessation.

3.5. Determine the proportion of individuals in whom cART is reinitiated due to viral rebound (2 consecutive pVL >2,000 copies/ml 3-5 days apart), >50% drop in CD4 cell counts, <450 cells/mm^3 and/or symptomatic acute retroviral syndrome.

3.6. Evaluate the emergence of viral resistance during MAP phase

3.7. Evaluate the viral suppression rates 6 months after treatment resumption.

ANNEX 3, OP.02.01.FLS-RS.02. Pre-estudi 14/10/2014

5.4 MEASURES TO AVOID BIAS

All patients will receive the same treatment in this study.

5.4.1 Randomization

Not applicable, since it is a single arm clinical trial.

5.4.2 Stratification

Not applicable, since it is a single arm clinical trial.

5.4.3 Blinding

Not applicable since it is an open clinical trial.

5.5 FORESEEN CALENDAR

- First patient first visit: February 2016
- Inclusion period: 6 months
- Follow-up period: At last 54 weeks (14 months)
- Last patient last visit: October 2017 (End of study)
- Final report submission: April 2018

5.6 END OF TRIAL

Stopping rules will be based on the observation of an unexpected high proportion of individuals experiencing SAE or any grade ≥3 local or systemic events judged definitely or probably related to any of the vaccinations and/or romidepsin administration.

If two or more study participants develop a clinically similar SAE or grade 3 local or systemic event, judged definitely, or probably related to the investigational medicinal products (IMP)s, a review by the Scientific Committee will be requested. The study will be suspended pending review of all safety data. Following this review, the Scientific Committee will make a recommendation to the Principal Investigator and Sponsor regarding continuation of the study (see section 9.3 for details)

In addition, if more than 50% of participants withdraw from the Scientific Committee will discuss a premature stop of the trial.

The date of the end of the trial will be to the last visit of the last patient.

5.6.1 Early study suspension

If the study must be interrupted prematurely, all non-used materials should be returned to the sponsor. The principal investigator will keep the investigator file and the completed CRF.

ANNEX 3, OP.02.01.FLS-RS.02. Pre-estudi 14/10/2014

In case there were no patients included in the study, the sponsor will take care of all materials.

5.7 SOURCE DATA

Source documents are the patient's medical records including routine laboratory results obtained from blood tests. Also specific forms such as RMD and MVA.HIVconsv administration forms (attachments B and C), SERAD questionnaire (attachment D), PK form (attachment W) and MAP Phase Monitoring Form (attachment E) will be considered source documents. A scanned copy of all source documents will be included in the patient’s medical chart, when possible; in all cases, these source documents will be included in the investigator site file.

Study data will be collected through an electronic Case Report Form (eCRF).

All investigational results from analysis performed from stored samples (virological, immunological results as well as PK determinations) will be recorded in electronic databases. Once the trial is finished, all databases will be unified.

Information regarding safety and tolerability of MVA.HIVconsv vaccinations and RMD administration will be collected using a patient diary (attachments F and G) which will be reviewed by the attending physician for grading and assessment of causality/imputability.

ANNEX 3, OP.02.01.FLS-RS.02. Pre-estudi 14/10/2014

6 TRIAL INVESTIGATIONAL PRODUCT(S)

6.1 EXPERIMENTAL TREATMENT

Two investigational medicinal products (IMP)s will be tested in this trial:

- Vaccine MVA.HIVconsv $2x10^8$ pfu, an investigational immunological product (vaccine) from a biological / biotechnological origin that contain genetically modified organisms.
- Romidepsin (Istodax®) 5mg/m^2, administered after antiemetic ondansetron 8mg intravenous (iv) infusion.

6.2 SUPPLY, PACKAGING, LABELING AND STORAGE

All investigational products will be stored at the Pharmacy Service of Hospital Universitari Germans Trias i Pujol, where reception, units, batch number and expiration date will be confirmed. No conditioning nor labelling will be required in the Pharmacy Service, except in case expiry date were to be extended for MVA.HIVconsv.

6.2.1 Romidepsin (RMD, Istodax®)

RMD is a potent HDACi approved in the US for the treatment of cutaneous T-cell lymphoma (CTCL) in patients who have received at least one prior systemic therapy and peripheral T-cell lymphoma (PTCL) in patients who have received at least one prior systemic theraphy whose marketing-authorization holder in Europe is Celgene Ltd. In Spain, RMD has only been used in clinical trials on hematology/oncology field under the investigation new drug application (PEI 07047). In the HIV field, RMD has been proposed as a potent HIV LRA.

RMD will be supplied by Celgene Ltd under a CTA in the form distributed by the technical director responsible for the development and control of the investigational product. RMD will be sent to the Parmacy Service of Hospital Universitari Germans Trias i Pujol, labelled for its use (only for BCN02-Romi trial).

Storage is to be performed in a safe place at 20°C to 25°C, excursions permitted between 15°C to 30°C.

Preparation will be performed at the Pharmacy Service by the study pharmacist and according to manufacturer indications.

6.2.2 MVA.HIVconsv

The MVA.HIVconsv is a non-marketed vaccine, and will be supplied by the University of Oxford under a CTA in the form distributed by the technical director responsible for the

ANNEX 3, OP.02.01.FLS-RS.02. Pre-estudi 14/10/2014

development and control of the investigational product. MVA.HIVconsv has been manufactured by IDT Biologika GmbH in Germany.
Storage is to be performed in a safe place at -80ºC, excursions permitted between -60ºC and -90ºC.

6.3 DOSE, INTERVAL, ROUTE AND METHOD OF ADMINISTRATION

Administration of MVA.HIVconsv and romidepsin will be performed in the Unitat Polivalent d'Investigació Clínica (UPIC) from Hospital Universitari Germans Trias i Pujol.

6.3.1 Romidepsin (RMD, Istodax®)

Dose: 5mg/m^2 over 4hours
Interval: weeks 3, 4 and 5

<u>Method of administration:</u>
Romidepsin is a cytotoxic drug and will be used with appropriate handling procedures. Study staff will follow specific Standard Operating Procedure for the handling, preparation, administration and disposal of romidepsin (attachment H) for the BCN02-Romi trial.

Briefly, RMD will first be reconstituted with the supplied diluent and further diluted in 500ml 0.9% Sodium Chloride Injection, using proper aseptic technique at the Pharmacy Service to deliver the desired dose.

Before administration at the UPIC, the drug will be inspected visually for particulate matter and discoloration. Romidepsin will be infused over 4 hours intravenously using an IV infusion pump.

According to investigator's brochure, patients will also receive antiemetic treatment before (ondansetron 8mg ev) and for 3 days after the infusion (8mg every 12hours, oral, starting on day of RMD administration).

Patients will be admitted on the night before the first RMD (RMD$_1$) infusion for a clinical and laboratory blood work and will be observed for the following 24hours. On RMD$_2$ and RMD$_3$, patients will be admitted at the UPIC on the morning of RMD infusion and will be observed for 12hours.

Data will be logged in a specific form (attachment B).

6.3.2 MVA.HIVconsv

Dose: 2x10^8 pfu

Interval: weeks 0 and 9.

ANNEX 3, OP.02.01.FLS-RS.02. Pre-estudi 14/10/2014

Method of administration: Study staff will follow specific Standard Operating Procedure for the handling, preparation, administration and disposal of vaccines (attachment I) for the BCN02-Romi trial.

Briefly, MVA.HIVconsv will be thawed until completely liquid in a biological level 2 (BL2) safety hood. The vial will not be shaken and the required volume will be drawn into the appropriate syringe. MVA.HIVconv will be administered within 1 hour of thawing. MVA.HIVconsv will be administered by intramuscular injection. MVA.HIVconsv will be administered into deltoid regions of both arms. The injection site will be observed for 30 minutes.

Data will be logged in a specific form (attachment C).

6.4 EXPERIMENTAL DRUG ACCOUNTABILITY

During the study, an accountability log, dispensing log and log of used vials will be kept for all IMPs. These logs will be monitored according to Good Clinical Practice (GPC) guidelines.

6.5 ARM DESCRIPTION

All patients will receive same experimental treatment, consisting of: MVA.HIVconsv vaccine administered 3 weeks before RMD_1 infusion and 4 weeks after RMD_3 infusion. Romidepsin infusions will be at weeks 3, 4 and 5 after first MVA.HIVconsv vaccine administration.

6.6 CONCOMITANT TREATMENTS

6.6.1 Ondansetron

Ondansetron is a marketed drug stated “generic” in Spain which is administered on a routine bases before the start of a potentially emetogenic chemotherapy. It will be supplied by the sponsor in the marketed form for iv (ondansetron 2mg/mL injectable solution) and oral (ondansetron 8mg tablet) administrations.

Ondansetron does not have particular temperature conservation, but vials should be in the exterior package to protect them from light. Ondansentron will be administered 30 minutes before RMD infusion (8mg iv) and during 3 days after the infusion (8mg tablet every 12 hours orally, starting on the day of RMD administration).

Preparation will be performed at the UPIC by the study nurse according to manufacturer indications. Once the product is diluted, infusion is to be performed in the next 24 hours and vial conserved between 2ºC and 8ºC.

ANNEX 3, OP.02.01.FLS-RS.02. Pre-estudi 14/10/2014

ANNEX 3, OP.02.01.FLS-RS.02. Pre-estudi 14/10/2014

6.6.2 cART

All individuals included will be under cART. To avoid drug interactions with romidepsin, all individuals will be on a regimen based in a non-boosted integrase inhibitor (raltegravir or dolutegravir based regimen). It is not expected any modification in the cART regimen all over the trial. In addition, a closely monitored antiretroviral pause is planned to offer cART resumption as soon as viral rebound is detected. A genotypic resistance test will be performed once viral rebound is confirmed to be >2,000 copies/ml. cART resumption regimen is expected to be still based on raltegravir or dolutegravir because neither raltegravir nor dolutegravir have an extended half-life that could favor the emergence of drug resistance short after treatment interruption, but new regimen decision will be to physician discretion.

6.6.3 Other

All other treatments administered during the study period will be considered concomitant treatments and should be documented in the CRF.

All participants will be discouraged to initiate any concomitant treatment without the knowledge and permission of the investigator. RMD is metabolized by CYP3A4. Patients will be discouraged of taking strong CYP3A4 inhibitors (ritonavir, cobicistat, atazanavir, ketoconazole, itraconazole, voriconazole, clarithromycin, telithromycin) or CYP3A4 inducers (nevirapie, efavirenz, etravirine, rifampicin, rifabutin, carbamazepine, phenytoin, phenobarbitalk, St. John’s Worth) from 2 weeks before RMD_1 until 2 weeks after RMD_3.

All participants will be discouraged to receive any vaccination (including Hepatitis B, Pneumococcal and Flu vaccinations among others) within 2 weeks of study entry and along the duration of the trial.

6.6.4 MODIFICATION OF THE ANTIRETROVIRAL TREATMENT REGIMEN

6.7 COMPLIANCE

MVA.HIVconsv and RMD compliance is guaranteed because study medication is to be administered by designated study nurse.

Antiretroviral treatment adherence will be self-reported by the patient by answering the adapted SERAD questionnaire (attachment D). No pill count will be performed to assess compliance. Ondansetron intake will be recorded in the diary card after each RMD administration (attachment G).

During the MAP period were cART is discontinued, plasma samples will be stored for future drug levels analyses for cases were antiretroviral consumption shall be suspected.

ANNEX 3, OP.02.01.FLS-RS.02. Pre-estudi 14/10/2014

7 SELECTION AND WITHDRAWAL OF SUBJECTS

7.1 INCLUSION CRITERIA

1. Subject included in ChAd-MVA.HIVconsv_BCN01 study with complete follow-up and included in BCN01-RO extension study.
2. Willing and able to give written informed consent for participation in the study
3. Willing and able to keep a good adherence to their cART regimen for the duration of the intervention phase.
4. Optimal virological suppression for at least 3 years defined as maintained pVL below limit of detection (based on current available assays, 20 or 40 copies/ml) allowing for isolated blips (<200 cop/ml, non-consecutive, >10% total determinations).
5. Being on a non-boosted integrase-inhibitor based regimen (raltegravir or dolutegravir) for at least 4 weeks at screening visit.
6. No new AIDS-defining diagnosis or progression of HIV-related disease.
7. Haematological and biochemical laboratory parameters as follows:
    a. Haemoglobin > 10g/dl
    b. Platelets > 100.000/dl
    c. ALT ≤ 2.5 x ULN
    d. Creatinine ≤ 1.3 x ULN
8. Serology: negative for hepatitis B surface antigen; negative for hepatitis C antibodies OR confirmed clearance of HCV infection (spontaneous or following treatment).
9. Available for follow up for duration of study (baseline + at the most 54 weeks) and willing to comply with the protocol requirements
10. Age ≥18 years
11. CD4 T cell count ≥500 cells/mm3

7.2 EXCLUSION CRITERIA

1. Confirmed HIV-2 seropositive
2. Positive pregnancy test. *
3. Presence of resistance drug mutations in the screening genotype **
4. Participation in another clinical trial within 12 weeks of study entry
5. History of autoimmune disease other than HIV-related auto-immune disease.
6. History or clinical manifestations of any physical or psychiatric disorder which could impair the subject’s ability to complete the study.
7. History of anaphylaxis or severe adverse reaction to vaccines.
8. Previous immunisation with any experimental immunogens (except for ChAd.HIVconsv and MVA.HIVconsv)
9. Receipt of blood products within 6 months of study entry
10. Treatment for cancer or lymphoproliferative disease within 1 year of study entry
11. Receipt of vaccines within 2 weeks of study entry and along the duration of the trial
12. Any other prior therapy which, in the opinion of the investigators, would make the individual unsuitable for the study or influence the results of the study

13. Current or recent use (within last 3 months) of interferon or systemic corticosteroids or other immunosuppressive agents

* Female subjects of childbearing potential must not be pregnant, not be planning a pregnancy or breast-feeding. Sexually active women must be willing to use two approved methods of contraception (including condoms, diaphragm, spermicides, hormonal methods and/or intrauterine devices) from screening until 28 days after last immunization. Sexually active men in heterosexual relationships must be willing to use two approved method of contraception with their partners from screening until 28 days after last immunization.

** Pre-cART genotype (performed at BCN01 screening visit)

7.3 SUBJECT WITHDRAWAL CRITERIA

7.3.1 Related to MVA.HIVconsv vaccination

First MVA.HIVconsv vaccination will only be administered if plasma viral load (pVL) is below the limit of detection in 2 consecutive determinations and CD4 cell count > 500 cells/ml at baseline. After RMD administration, low-level viral load detections (pVL<2,000 copies/ml) will be allowed only if suboptimal cART adherence is not suspected.

Any individual for who is being considered for discontinuation or postponement of vaccinations will be discussed with the trial team.

Participants might be discontinued or postponed -if clinical condition or laboratory abnormality is resolved within a maximum window of 10 days- from further vaccination for any of the following reasons:

- Ineligibility (either arising during the study or retrospective having been overlooked at screening).
- A disease, condition or an adverse event (including clinically significant abnormal laboratory values; see below) that develops, regardless of relationship to the study products, if, in the opinion of the principal investigator or designee, further vaccinations would jeopardize the safety of the patient.
- Hematology
    - Hemoglobin < 10.0 g/dl
    - Absolute Neutrophil Count (ANC) ≤ 1000 /mm^3 (≤ 1 x 10^9 /l)
    - Absolute Lymphocyte Count (ALC) ≤ 600 /mm^3 (≤ 1 x 10^9 /l)
    - Platelets ≤ 50.000 /mm^3, ≥ 550,000 /mm^3 (≤ 50 /L, ≥ 550 /l)
- Biochemistry

ANNEX 3, OP.02.01.FLS-RS.02. Pre-estudi 14/10/2014

- Creatinine > 1.3 x ULN
- AST > 2.5 x ULN
- ALT > 2.5 x ULN

- Confirmed HIV-1 or HIV-2 re-infection.
- Pregnancy.
- Significant non-compliance with cART regimen or study requirements.
- Receipt of blood transfusion or blood products during this study.
- Participating in another clinical trial of an IMP.
- A serious adverse event judged to be possibly, probably or definitely related to vaccination.
- An adverse event which requires discontinuation of the study product or results in inability to continue to comply with study procedures.
- Loss to follow up.
- The safety of the volunteer would be jeopardized in the opinion of the investigator or sponsor.
- Investigator discretion.
- Significant protocol deviation.
- Disease progression, including virological failure (defined as a rebound in at least two consecutive determinations of pVL > 2,000 copies/ml after suppression), which requires discontinuation or modification of the study medication or results in inability to continue to comply with study procedures. Low-level pVL determinations just after vaccinations and/or during RMD administration will be allowed only if suboptimal cART adherence is not suspected.
- Consent withdrawn.

7.3.2 Related to romidepsin administration

Any individual for who is being considered for discontinuation or postponement of RMD administrations will be discussed with the trial team.

ANNEX 3, OP.02.01.FLS-RS.02. Pre-estudi 14/10/2014

Participants might be discontinued or postponed -if clinical condition or laboratory abnormality is resolved within a maximum window of 7 days- from further romidepsin administration for any of the following reasons:

- Ineligibility (either arising during the study or retrospective having been overlooked at screening).
- A disease, condition or an adverse event (including clinically significant abnormal laboratory values; see below) that develops, regardless of relationship to the study products, if, in the opinion of the principal investigator or designee, further vaccinations would jeopardize the safety of the patient.
- Hematology
  - Hemoglobin < 10.0 g/dl
  - Absolute Neutrophil Count (ANC) ≤ 1000 /mm^3 (≤ 1 x 10^9 /l)
  - Absolute Lymphocyte Count (ALC) ≤ 600 /mm^3 (≤ 1 x 10^9 /l)
  - Platelets ≤ 50.000 /mm^3, ≥ 550,000 /mm^3 (≤ 50 /L, ≥ 550 /l)
- Biochemistry
  - Creatinine > 1.3 x ULN
  - AST > 2.5 x ULN
  - ALT > 2.5 x ULN
- Confirmed HIV-1 or HIV-2 re-infection.
- Pregnancy.
- Significant non-compliance with cART regimen or study requirements.
- Receipt of blood transfusion or blood products during this study.
- Participating in another clinical trial of an IMP.
- A serious adverse event judged to be possibly, probably or definitely related to romidepsin administration.
- An adverse event which requires discontinuation of the study product or results in inability to continue to comply with study procedures.
- Loss to follow up.
- The safety of the volunteer would be jeopardized in the opinion of the investigator or sponsor.
- Investigator discretion.

ANNEX 3, OP.02.01.FLS-RS.02. Pre-estudi 14/10/2014

- Significant protocol deviation.

- Disease progression, including virological failure (defined as a rebound in at least two consecutive determinations of pVL > 2,000 copies/ml after suppression), which requires discontinuation or modification of the study medication or results in inability to continue to comply with study procedures. Low-level pVL determinations just after vaccinations and/or during RMD administration will be allowed only if suboptimal cART adherence is not suspected.

- Consent withdrawn.

7.3.3 Related to cART interruption

As persistent detectable viral replication may induce immune activation that could favor a faster viral rebound after cART cessation, cART interruption will only be indicated if plasma viral load (pVL) is below the limit of detection in 2 consecutive determinations, spaced at least 1 week, at MAP visit (MVA2+8w). In case that MVA.HIVconsv second booster immunization may induce repetitive detectable plasma HIV-1 RNA determination still present at MAP visit , cART interruption will be postponed until 2 consecutive determinations are achieved

Any individual for who is being considered for postponement of cART interruption will be discussed with the trial team.

7.3.4 Early subject withdrawal

The patients will complete the clinical study before the stipulated time in the following circumstances:

- Concurrent process or illness which in the opinion of the investigator requires the withdrawal of the patient.

- Protocol deviation which in the opinion of the sponsor requires the withdrawal of the patient.

- The patient does not wish to continue in the study.

- Other

On the other side and in order to minimize risks of the treatment interruption intervention, an early interim and futility analyses is planned before the MAP phase for each individual. Treatment interruption would be discouraged if boosting MVA.HIVconsv is shown to be no longer immunogenic after romidepsin administration –defined as no net increase of HIVconsv-specific IFNg response compared to pre-vaccination levels with MVA.HIVconsv at BCN02-Romi baseline visit, due to RMD toxicity on CTL functional

ANNEX 3, OP.02.01.FLS-RS.02. Pre-estudi 14/10/2014

ANNEX 3, OP.02.01.FLS-RS.02. Pre-estudi 14/10/2014

activity for instance, as an improved viral control would not be foreseen. These results are considered valid for up to 12 weeks from the second vaccination. As vaccine induced T-cells may decrease in the following weeks and available data on expected kinetics of immune response is limited, in the event that the cART interruption is postponed more than MVA2 + 12w, the futility analyses should be repeated and results will be discussed among the investigators
In such situation, trial results would be focused on the viral reservoir and immunogenicity endpoints only until better correlates of control would be available to predict clinically relevant outcomes on which one could recommend treatment interruptions in a safer manner.

7.3.5 Medical approach to withdrawal

In all cases, 'end of study form' is to be filled. Detailed information will be given about the date and reasons of the discontinuation to the sponsor. The investigator will facilitate the necessary medical support to the patient.

7.3.6 Follow-up after early withdrawal

That is, as a general rule, all patients who discontinue treatment prematurely will undergo a clinical examination and all tests specified in the visit.

In case early withdrawal happens while patient is receiving IMP, a follow-up of 28 days after the last IMP dose will be performed for AEs collection.

7.3.7 Replacement of patients

Replacement of patients will not be possible, since the number of candidates is limited to the participants of the BCN01-RO

7.4 PRE-RANDOMIZATION / PRE-BASELINE LOSSES

Data from patients that do not meet the selection criteria after completing the screening visit will not be considered for the study, but the reason for not meeting selection criteria will be recorded in the screening log form in an anonimised manner.

ANNEX 3, OP.02.01.FLS-RS.02. Pre-estudi 14/10/2014

8 TRIAL CONDUCTION AND RESPONSE EVALUATION

8.1 TRIAL DEVELOPMENT

Participants included in the extension phase of the BCN01 trial (BCN01-RO) will be contacted by principal investigator or collaborators and participation into the BCN02-Romi will be offered. A meeting with the investigator will be scheduled, if they wish. Participants will be provided with information on the MVA.HIVconsv vaccine and romidepsin and the study protocol and will be offered the Participant Information Sheet. Patients will have the opportunity to ask questions and to arrange an appointment for a screening visit if they wish. In the scheduling of screening visits it will be ensured that potential participants have adequate time to decide whether to participate.

Screening visit will be scheduled.

Detailed Schedule of Procedures in every visit is found in attachment J.

8.1.1 SCR – Screening (week -1)

SCR visit will take place in the HIV units of both HGTIP and HC.
Site personnel will obtain informed consent. After patient acceptance, inclusion and exclusion criteria will be reviewed and following procedures will be performed:

- Data collection: demographics, baseline HIV drug resistance genotype information, last hepatitis C and B, and syphilis serology, last laboratory tests, history of cART regimens, history of past and foreseen vaccinations.
- cART adherence assessment (SERAD questionnaire)
- Blood extraction (if no lab tests available within 3 months or Hepatitis B/C or syphilis RPR within 6 months prior to screening visit), including pregnancy test.
- Plasma and PBMC storage following scheme detailed in attachment K
- Provide a calendar with all the planned visits.
- MVA_1 and MVA_1+1w visits scheduling

8.1.2 MVA1 – First MVA vaccination (week 0)

Before MVA_1 visit, study physician will review all inclusion and exclusion criteria and if all vaccination criteria are met, will prescribe the vaccination using the **MVA.HIVconsv prescription form** (attachment L).

MVA_1 visit will take place in UPIC (HGTIP) and by FLS study nurse and FLS physician.

In MVA_1 visit, procedures to be performed will be:

- Measurement of vital constants (blood pressure, temperature and cardiac rate)
- cART adherence assessment (SERAD questionnaire)
- Clinical visit including physical exam by the investigator physician pre and post vaccination
- Collection of MVA.HIVconsv vaccine from the Pharmacy Service

ANNEX 3, OP.02.01.FLS-RS.02. Pre-estudi 14/10/2014

- Preparation of MVA.HIVconsv vaccine in the designated BL2 sterile hood located at IrsiCaixa following handling, thawing and preparation instructions detailed at the SOP (attachment I)
- Intramuscular administration of MVA.HIVconsv vaccine in both deltoid muscles
- Measurement of vital constants 10 and 30 minutes after MVA injection.
- Fill in the **MVA.HIVconsv administration form** (attachment C)
- Provide the patient with the **MVA diary card** (attachment F).
- Give instructions to fill diary card.

8.1.3 MVA1+1w – One week post- MVA1 vaccination (week 1)

The following procedures will be performed at both HIV units (HGTIP and HC):

- Blood extraction including:
    - Viral load, biochemistry (including Sodium, Potassium, Phosphate, Magnesium and Calcium) and hematological parameters.
    - Plasma and PBMC storage following scheme detailed in attachment K
- cART adherence assessment (SERAD questionnaire)
- Revision of MVA diary card and record of adverse events in clinical records
- Clinical visit, adverse event register
- RMD_1 visit explanation and scheduling
- CRF data collection

8.1.4 RMD1 – First romidepsin infusion (week 3)

RMD_1 visit will take place in UPIC (HGTIP). Patients will be admitted on the day before the administration (no later than 8pm).

During the evening before RMD administration, the following procedures will be performed by the UPIC study nurse:

- Take vital constants (blood pressure, temperature and cardiac rate)
- Blood extraction including:
    - Biochemistry and hematological parameters via Hospital emergency laboratory.
- Pregnancy test
- Electrocardiogram (ECG)
- cART adherence assessment (SERAD questionnaire)

In the morning of RMD administration, the UPIC study physician will perform a clinical visit, review ECG and lab test results and will fill in the **romidepsin/ondansetron prescription form**. The study nurse will perform:

- Blood extraction including:
    - Viral load, biochemistry (including Sodium, Potassium, Phosphate, Magnesium and Calcium) and hematological parameters and CD4 count.
    - Plasma and PBMC storage following scheme detailed in attachment K (pre-RMD infusion, -4 hour time point of PK_1)
- Review romidepsin and ondansetron prescription
- Administrate ondansetron iv.

- Collect romidepsin and ondansetron tablets from Pharmacy Service (already prepared by pharmacy personnel)
- Take vital constants (blood pressure, temperature and cardiac rate)
- Administrate romidepsin (4-hour infusion)
- Collect PK_1 samples (see attachment K)
- Measurement of vital constants (blood pressure, temperature and cardiac rate) in the first 30 minutes after RMD infusion and within 30 minutes after RMD finalization
- Perform ECG within 30 minutes after RMD infusion, to be reviewed by study physician.
- Fill in **RMD_1 administration form** (attachment B)
- Provide the patient with the **RMD diary card** (attachment G)
- Give instructions to fill in the diary card / fill in together while admitted.
- Give instructions to take ondansetron tablets. Provide patient with 6 ondansetron tablets. Take first ondansetron tablet while admitted.
- Both Study nurse and Study physician will monitor for appearance of clinical adverse events

In the morning of day after RMD administration, following procedures will be performed:

- Clinical visit by the investigator physician
- Measurement of vital constants (blood pressure, temperature and cardiac rate)
- Blood extraction including:
  - Plasma and PBMC storage following scheme detailed in attachment K (post-RMD infusion, 20 hour time point of PK_1).

8.1.5 RMD1 + 3d – 3 days post RMD1 infusion (week 3+ 72h)

Following procedures will be performed at both HIV units (HGTIP and HC):

- Blood extraction including:
  - Viral load, biochemistry (including Sodium, Potassium, Phosphate, Magnesium and Calcium) and hematological parameters and CD4 count
  - Plasma and PBMC storage following scheme detailed in attachment K
- Revision of RMD diary card, lab tests and record of adverse events in clinical records
- Clinical visit, adverse event register
- RMD_2 visit explanation and scheduling
- CRF data collection

8.1.6 RMD2 – Second romidepsin infusion and MVA1+4w – Four weeks post MVA1 vaccination (week 4)

The day before of RMD_2 visit, study physician will review lab test results from previous visit and will fill in the **romidepsin/ondansetron prescription form**.

RMD_2 visit will take place in UPIC (HGTIP). Patient will be admitted in the morning of administration (7:30am). Following procedures will be performed:

ANNEX 3, OP.02.01.FLS-RS.02. Pre-estudi 14/10/2014

- Blood extraction including:
  - o Viral load, biochemistry (including Sodium, Potassium, Phosphate, Magnesium and Calcium), hematological parameters and CD4 count
  - o Plasma and PBMC storage following scheme detailed in attachment K (pre-RMD infusion, -4 hour time point of PK_2)
- Urine pregnancy test
- Revision of romidepsin and ondansetron prescription
- Administration of ondansetron ev.
- Collection of romidepsin and ondansetron tablets from Pharmacy Service (already prepared by pharmacy personnel)
- Measurement of vital constants (blood pressure, temperature and cardiac rate)
- Clinical visit by the investigator physician pre and post romidepsin infusion and revision of RMD diary card.
- Administration of romidepsin (4-hour infusion)
- Collection of PK_2 samples (see attachment K)
- Measurement of vital constants (blood pressure, temperature and cardiac rate) in the first 30 minutes after RMD infusion and within 30 minutes after RMD finalization
- Perform ECG within 30 minutes after RMD infusion to be reviewed by study physician.
- Fill in **RMD_2 administration form** (attachment B)
- Provide the patient with the **RMD diary card** (attachment G)
- Give instructions to fill in the diary card / fill in together while admitted.
- cART adherence assessment (SERAD questionnaire)
- Both Study nurse and Study physician will monitor for appearance of clinical adverse events
- Give instructions to take ondansetron tablets. Provide patient with 6 ondansetron tablets. Take first ondansetron tablet while admitted.
- RMD_2+3d visit scheduling

ANNEX 3, OP.02.01.FLS-RS.02. Pre-estudi 14/10/2014

8.1.7 RMD2 + 3d – 3 days post RMD2 infusion (week 4+72h)

Following procedures will be performed at both clinical sites (HGTIP and HC):

- Blood extraction including:
  - o Viral load, biochemistry (including Sodium, Potassium, Phosphate, Magnesium and Calcium) and hematological parameters and CD4 count
  - o Plasma and PBMC storage following scheme detailed in attachment K
- Revision of RMD diary card, lab tests and record of adverse events in clinical records
- Clinical visit, adverse event collection
- RMD_3 visit explanation and scheduling
- CRF data collection

8.1.8 RMD3 – Third romidepsin infusion (week 5)

Before RMD_3 visit, study physician will review lab test results from previous visit and will fill in the **romidepsin/ondansetron prescription**.

RMD_3 visit will take place in UPIC (HTIP). Patients will be admitted in the morning (07:30am). Following procedures will be performed:

- Blood extraction including:
    - o Viral load, biochemistry (including Sodium, Potassium, Phosphate, Magnesium and Calcium), hematological parameters and CD4 count
    - o Plasma and PBMC storage following scheme detailed in attachment K (pre-RMD infusion, -4 hour time point of PK_3)
- Urine pregnancy test
- Revision of romidepsin and ondansetron prescription
- Administration of ondansetron ev.
- Collection of romidepsin and ondansetron tablets from Pharmacy Service (already prepared by pharmacy personnel)
- Measurement of vital constants (pressure, temperature and cardiac frequency)
- Clinical visit by the investigator physician pre and post romidepsin infusion and revision of RMD diary card.
- Administration of romidepsin (4-hour infusion)
- Collection of PK_3 samples (attachment K)
- Measurement of vital constants (blood pressure, temperature and cardiac rate) in the first 30 minutes after RMD infusion and within 30 minutes after RMD finalization
- Perform ECG within 30 minutes after RMD infusion to be reviewed by study physician.
- Fill in **RMD_3 administration form** (attachment B).
- Provide the patient with the **RMD diary card** (attachment G).
- Give instructions to fill in the diary card / fill in together while admitted.
- cART adherence assessment (SERAD questionnaire)
- Both Study nurse and Study physician will monitor for appearance of clinical adverse events
- Give instructions to take ondansetron tablets. Provide patient with 6 ondansetron tablets. Take first ondansetron tablet while admitted.
- RMD_3+3d visit scheduling

ANNEX 3, OP.02.01.FLS-RS.02. Pre-estudi 14/10/2014

8.1.9 RMD3 + 3d – 3 days post RMD3 infusion (week 5 +72h)

Following procedures will be performed at both HIV units (HGTIP and HC):

- Blood extraction including:
    - o Viral load, biochemistry (including Sodium, Potassium, Phosphate, Magnesium and Calcium) and hematological parameters and CD4 count
    - o Plasma and PBMC storage following scheme detailed in attachment K
- Revision of RMD diary card, , lab tests and record of adverse events in clinical records
- Clinical visit, adverse event collection
- RMD_3 + 1w visit explanation and scheduling
- CRF data collection

8.1.10 RMD3 + 1w – One week post RMD3 infusion (week 6)

Following procedures will be performed at both HIV units (HGTIP and HC):

- Blood extraction including:
    - o Viral load, biochemistry and hematological parameters and CD4 count

  - o Plasma and PBMC storage following scheme detailed in attachment K
- Revision of RMD diary card, lab tests and record of adverse events in clinical records
- cART adherence assessment (SERAD questionnaire)
- Clinical visit, adverse event collection
- MVA_2 visit explanation and scheduling
- CRF data collection

8.1.11 MVA2 – Second MVA vaccination and MVA1+9w – Nine weeks post MVA1 vaccination (week 9)

Before MVA_2 visit, study physician will review all vaccination criteria are met and will fill the **vaccination prescription form**.

MVA_2 visit will take place in UPIC (HGTIP).

In MVA_2 visit, as soon as patient arrives to UPIC, following procedures will be performed:

- Blood extraction including:
  - o Plasma and PBMC storage following scheme detailed in attachment K
- Measurement of vital constants (blood pressure, temperature and cardiac rate)
- cART adherence assessment (SERAD questionnaire)
- Clinical visit including physical exam by the investigator physician pre and post vaccination
- Collection of MVA.HIVconsv vaccine from the Pharmacy Service
- Preparation of MVA.HIVconsv vaccine in the designated BL2 sterile hood located at IrsiCaixa following handling, thawing and preparation instructions detailed at the SOP (attachment I)
- Intramuscular administration of MVA.HIVconsv vaccine in both deltoid muscles
- Measurement of vital constants 10 and 30 minutes after MVA injection.
- Fill in the **MVA.HIVconsv administration form** (attachment C)
- Provide the patient with the **MVA diary card** (attachment F).
- Give instructions to fill in the diary card.
- MVA_2+1w visit scheduling

8.1.12 MVA2+1w – One week post- MVA2 vaccination (week 10)

Following procedures will be performed at both HIV units (HGTIP and HC):
- Blood extraction including:
  - o Viral load, biochemistry and hematological parameters.
  - o Plasma and PBMC storage following scheme detailed in attachment K
- cART adherence assessment (SERAD questionnaire)
- Revision of MVA diary card and record of adverse events in clinical records
- Clinical visit, adverse event register
- MVA_2 +4w visit explanation and scheduling
- CRF data collection

8.1.13 MVA2+4w – Four weeks post- MVA2 vaccination (13 weeks)

Following procedures will be performed at both clinical sites (HGTIP and HC):
- Blood extraction including:
  - o Viral load, biochemistry and hematological parameters and CD4 count.

ANNEX 3, OP.02.01.FLS-RS.02. Pre-estudi 14/10/2014

ANNEX 3, OP.02.01.FLS-RS.02. Pre-estudi 14/10/2014

    - Plasma and PBMC storage following scheme detailed in attachment K
- cART adherence assessment (SERAD questionnaire)
- Clinical visit, adverse event register
- MVA_2 +8w visit explanation and scheduling
- CRF data collection

In cases where pVL is detected >20 copies/mL at MVA2 + 4w, an extra visit will be scheduled at MVA2 + 6w including the following procedures:

- Blood extraction including:
    - Viral load, biochemistry,hematological parameters and CD4 count.
    - Plasma and PBMC storage.
- cART adherence assessment (SERAD questionnaire)
- Clinical visit, adverse event register
- MVA_2 +8w visit explanation and scheduling
- CRF data collection

8.1.14 MAP – Monitored antiretroviral pause and MVA2+8w – Eight weeks post MVA2 vaccination (17 weeks)

Following procedures will be performed at both clinical sites (HGTIP and HC):

- Blood extraction including:
    - Viral load, biochemistry and hematological parameters.
    - Plasma and PBMC storage following scheme detailed in attachment K
- cART adherence assessment (SERAD questionnaire)
- Clinical visit, adverse event register
- Review acceptability for cART interruption after interim analyses (which will be performed at week 13 and be provided by investigational team in advance and recorded in 'Indication for MAP phase' form, attachment X)
- Review acceptability for cART interruption regarding plasma viral load suppression (pVL below the limit of detection in 2 consecutive determinations, spaced at least 1 week, and recorded in 'Indication for MAP phase' form, attachment X)
- Phase 2 study explanation including: cART interruption, site, weekly clinical visit, qualitative plasma viral load monitoring, criteria for cART resumption.
- CRF data collection

Once analytical results are available, cART interruption will be confirmed telephonically by study physician and first visit in BCN-Checkpoint will be scheduled.

In cases where pVL is not undetectable for 2 consecutive determinations, MAP will be postponed and extra visits will be scheduled (every week if pVL is 20-500 copies/mL, every two weeks if pVL is >500 copies/mL) including the following procedures until acceptability criteria for cART interruption are met.

- Blood extraction including:
    - Viral load, biochemistry and hematological parameters.
    - Plasma and PBMC storage.
- cART adherence assessment (SERAD questionnaire)
- Clinical visit, adverse event register

ANNEX 3, OP.02.01.FLS-RS.02. Pre-estudi 14/10/2014

- CRF data collection

  In cases where MAP is postponed after MVA2+12w, the ELISPOT immunomonitoring assay will be repeated to assess if the vaccine induced T cell responses has decreased significantly. The research team will consider both the immunogenicity analysis and dynamics of plasma viremia to indicate or discourage treatment cessation.

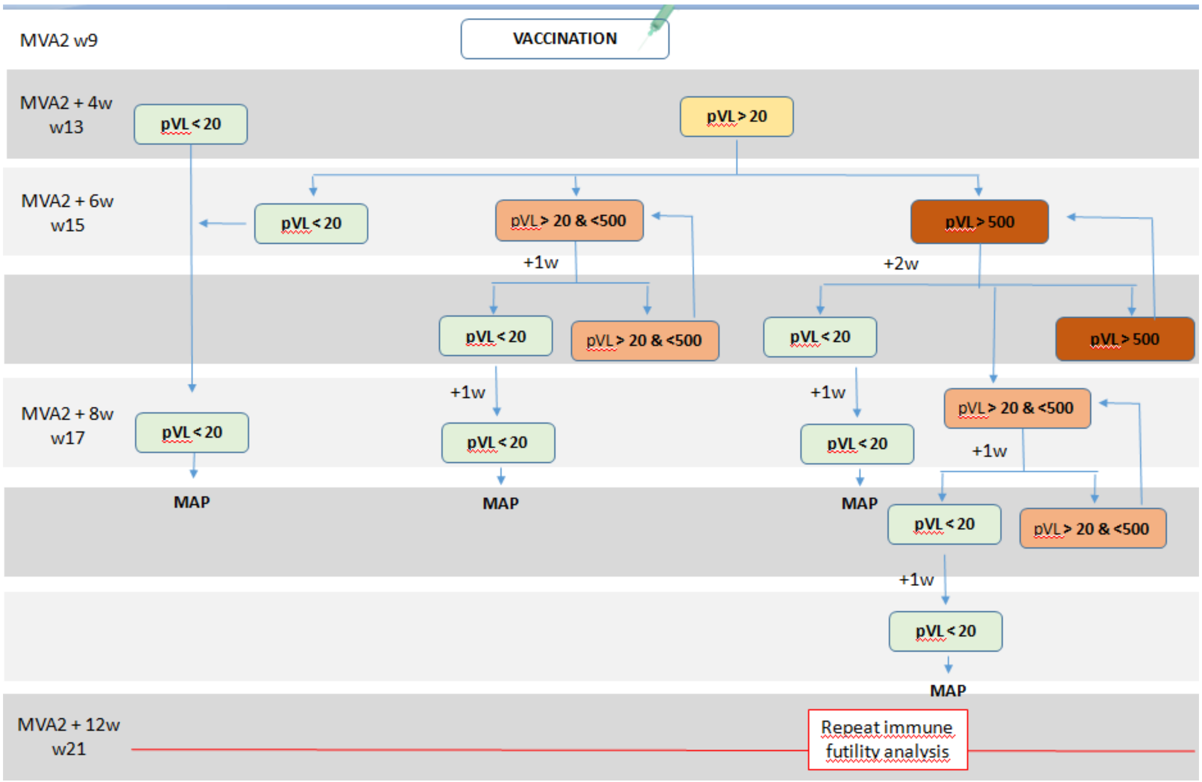

8.1.15 MAP phase monitoring

Monitoring visits during the MAP phase will be performed at BCN-Checkpoint community center (see flow chart below). A specific MAP Phase Monitoring Form (attachment E) will be used to collect data.

During first 12 weeks post-MAP (from week 18 to week 29):

- Weekly visits will be performed, including:
  - Clinical visit and adverse events register
  - Reporting of symptoms suggestive of acute retroviral syndrome (including its severity grade):

☐ Fever of unkown origin	☐ Headache	☐ Pharyngitis
☐ Lymphadenopathy	☐ Transaminase elevation	☐ Lymphopenia
☐ Oral candidiasis	☐ Myalgia/arthralgia	☐ Night sweats

☐ Fatigue	☐ Nausea, vomiting	☐ Hepato/splenomegaly
☐ Rash	☐ Diarrhea	☐ Others
☐ Trombochytopenia		

- 5ml blood extraction for :
  - Qualitative viral load test (Xpert HIV-1 Qual de Cepheid)
- Discussion on treatment resumption: recommendation on cART resumption will be assessed based on clinical visit, willingness of patients to resume treatment and detection of viral load in the qualitative test.
- Schedule of next appointment.

- Visits at 2, 4, 8, and 12 weeks after treatment interruptions (weeks 19, 21, 25 and 29) will also include
  - Biochemistry and hematological parameters, viral load and CD4 count (performed at an external Lab)
  - Plasma and PBMC storage following scheme detailed in attachment K
  - Discussion on treatment resumption once results from Biochemistry, Hematological parameters and CD4 cell count are available (72h): recommendation on cART resumption will be assessed based on clinical visit, willingness of patients to resume treatment, detection of viral load in the qualitative test and/or drop in CD4 cell counts.

After the first 12 weeks post-MAP (from W30):

- Fortnightly visits will be performed, including:
  - Qualitative viral load test (Xpert HIV-1 Qual de Cepheid)
  - Clinical visit, adverse event register
  - Reporting of symptoms suggestive of acute retroviral syndrome (including its severity grade)
  - Discussion on treatment resumption: recommendation on cART resumption will be assessed based on clinical visit, willingness of patients to resume treatment and detection of viral load in the qualitative test.

- Monthly:
  - Biochemistry and hematological parameters, viral load and CD4 count (performed at an external Lab)
  - Plasma and PBMC storage following scheme detailed in attachment K
  - Discussion on treatment resumption once results from Biochemistry, Hematological parameters and CD4 cell count are available (72h): recommendation on cART resumption will be assessed based on clinical visit, willingness of patients to resume treatment, detection of viral load in the qualitative test and/or drop in CD4 cell counts.

ANNEX 3, OP.02.01.FLS-RS.02. Pre-estudi 14/10/2014

ANNEX 3, OP.02.01.FLS-RS.02. Pre-estudi 14/10/2014

	Phase 2 (to be performed at BCN-Checkpoint)																																	
	1 to 12 weeks of MAP												After the first 12 weeks of MAP *(in case of no-rebound)*																					
**Weeks (MAP)**	**1**	**2**	**3**	**4**	**5**	**6**	**7**	**8**	**10**	**11**	**12**	**13**	**14**	**15**	**16**	**17**	**18**	**19**	**20**	**21**	**22**	**23**	**24**	**25**	**26**	**27**	**28**	**29**	**30**	**31**	**32**	**33**	**34**
Window (+/-)	2d	2d	2d	2d	2d	2d	2d	2d	2d	2d	2d	2d	2d	2d	2d	2d	2d	2d	2d	2d	2d	2d	2d	2d	2d	2d	2d	2d	2d	2d	2d	2d	2d
Qualitative pVL (HIV-1 Xpert Qual)	x	x	x	x	x	x	x	x	x	x	x		x		x		x		x		x		x		x		x		x		x		x
Clinical visit	x	x	x	x	x	x	x	x	x	x	x		x		x		x		x		x		x		x		x		x		x		x
Safety (AE and SAE)	x	x	x	x	x	x	x	x	x	x	x		x		x		x		x		x		x		x		x		x		x		x
Concomitant treatment	x	x	x	x	x	x	x	x	x	x	x		x		x		x		x		x		x		x		x		x		x		x
Biochemistry¬		x		x				x			x				x				x				x				x				x		
Haematology¬		x		x				x			x				x				x				x				x				x		
CD4 cell count¬		x		x				x			x				x				x				x				x				x		
Quantitative HIV-1 pVL¬		x		x				x			x				x				x				x				x				x		
Plasma and PBMC storage#		x		x				x			x				x				x				x				x				x		
Efficacy Endpoint											x																						
# to be shipped to HGTIP for processing in the same day (for virological and immunological studies)																																	
¬ to be shipped to General Lab : results available at Checkpoint (Report by phone to the patients at 72h)																																	

If qualitative pVL test is found positive (indicative of pVL >200 copies/ml), following procedures will be performed:

- Blood extraction including:
  - Viral load, hematological parameters and CD4 count
  - Plasma and PBMC storage following scheme detailed in attachment K
  - pVL-pos+3d visit scheduling

pVL-pos+3d visit will be performed at BCN-Checkpoint and will include:
- Clinical visit, adverse event collection
- Reporting of symptoms suggestive of acute retroviral syndrome (including its severity grade)
- Blood extraction including:
  - Viral load and CD4 count
  - Genotype resistance test
  - Plasma and PBMC storage following scheme detailed in attachment K
- cART resumption visit scheduling (pVL-pos + 7 days) at BCN-Checkpoint

8.1.16 cART resumption visit (7-10 days after first Qual pVL positive)

pVL-pos+7d visit will be performed at BCN-Checkpoint.

Following procedures will be performed:

- Reporting of symptoms suggestive of acute retroviral syndrome (including its severity grade)
- Discussion on treatment resumption based on following considerations (attachment M):
  - If viral load is >2,000 copies/mL in visits pVL-pos and pVL-pos+3d, and/or clinical symptoms, laboratory abnormalities or significant drops in CD4 T cell counts (>50% &/or <500) have been detected, patients will be recommended to re-start cART immediately.
  - If viral load is <2,000 copies/mL in visits pVL-pos and >2,000 copies/mL in visit pVL-pos+3d, and/or clinical symptoms, laboratory abnormalities or

significant drops in CD4 T cell counts (>50% &/or <500) have been detected, patients will be recommended to re-start cART immediately.
- o If viral load is > or <2,000 copies/mL in visits pVL-pos and <2,000 copies/mL in visit pVL-pos+3d, and NO clinical symptoms, laboratory abnormalities or significant drops in CD4 T cell counts have been detected cART will not be resumed.

- cART prescription* at the Pharmacy Unit
- Follow-up explanation and cART+4w visit scheduling (or a follow-up MAP visit at BCN-Checkpoint if cART is not resumed)
- CRF data collection

*cART regimen will at the discretion of the study physician, according to clinical criteria (no changes on patient's previous cART regimen are envisioned as no emergence of drug resistances is expected)

Overall, time since 1st positive HIV-1 Qualitative detection to cART resumption is aimed to be <10 days to avoid fast viral reservoir replenishment.

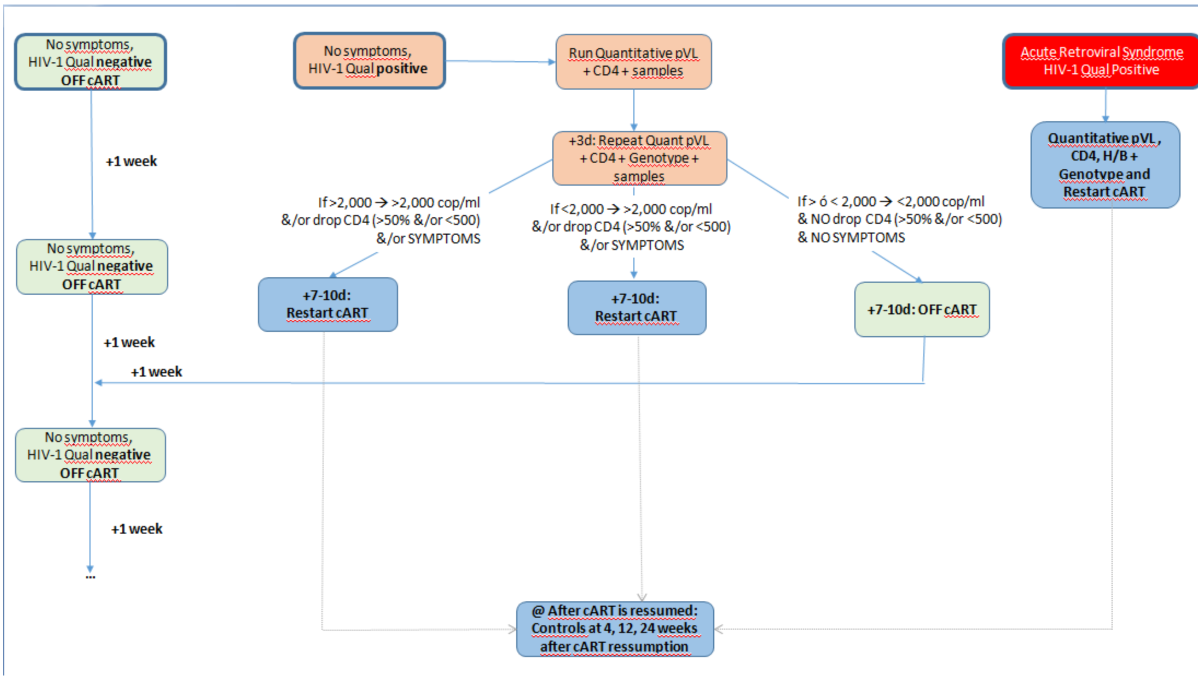

8.2 CART+4W, CART+12W – CART FOLLOW-UP VISITS (4 AND 12 WEEKS AFTER CART RESUMPTION)

As when naïve HIV positive individuals start 1st line treatment, all individuals will be followed to monitor clinical, virological and immune outcomes after cART resumption. Following procedures will be performed:
- Blood extraction including:
  - o Viral load, biochemistry and hematological parameters and CD4 count
  - o Plasma and PBMC storage following scheme detailed in attachment K

ANNEX 3, OP.02.01.FLS-RS.02. Pre-estudi 14/10/2014

- Clinical visit, adverse event register
- cART adherence assessment (SERAD questionnaire)
- Next visit scheduling
- CRF data collection

8.2.1 cART+24w – end of trial visit (24 weeks after cART resumption)

Following procedures will be performed:

- Blood extraction including:
  - Viral load, biochemistry and haematological parameters and CD4 count
  - Plasma and PBMC storage following scheme detailed in attachment K
- Clinical visit, adverse event register
- cART adherence assessment (SERAD questionnaire)
- Explain to the patient that the study is finished. Schedule visits with regular physician according to routine clinical practice.
- CRF data collection

8.3 LONG-TERM FOLLOW-UP

After end-of-study, participants will continue with cART and return to standard clinical care in consultation with their doctor, in HIV units from HGTIP and HC. All participants will be followed-up annually for 5 years in regards to:

- Safety
- HIV related information
- Gene transcription studies

8.4 PROCEDURES FOR EVALUATION OF RESPONSE

8.4.1 Clinical record and physical exam

Demographic and HIV infection-related data will be collected in order to characterize the study population (sex, age, time since HIV transmission to treatment initiation, Fiebig stage at HIV diagnosis, risk factor and history of opportunistic infections or tumors).

Appearance of clinical adverse events will be recorded all over the study. Appearance of acute retroviral symptoms (Fever of unkown origin, Headache, Pharyngitis, Lymphadenopathy, Myalgia/arthralgia, Night sweats, Fatigue, Nausea, vomiting, Hepato/splenomegaly, Rash, Diarrhea, Others) will be specifically recorded during the MAP phase.

A complete physical examination will be performed at the baseline visit, including weight and height. In the follow-up visits, a physical exam will be performed following schedule described in attachment J (Schedule of procedures).

ANNEX 3, OP.02.01.FLS-RS.02. Pre-estudi 14/10/2014

ANNEX 3, OP.02.01.FLS-RS.02. Pre-estudi 14/10/2014

8.4.2 Laboratory tests

Patients will fast for at least 8 hours prior to assessment, in the points specified in the schedule of procedures of the study (attachment J), except for RMD_1 assessment that will be performed in the evening previous RMD_1 administration. The following parameters will be quantified, as needed:

- **Hematology:**
  - Hematocrit
  - Red blood cell count
  - Hemoglobin
  - Leucocytes
  - Lymphocyte
  - Platelet count

- **Blood biochemistry:**
  - Urea
  - Creatinine
  - Ionogramme: sodium, potassium
  - Total Bilirubin
  - Total protein
  - Albumin
  - Liver enzymes: aspartate aminotransferase (AST), alanine aminotransferase (ALT), gamma-GT, alkaline phosphatase
  - Estimated glomerular filtration rate (CKD-EPI)
  - Creatinin Kinase (CK)

  Extra ions: Magnesium, phosphate and calcium

- **Pregnancy test in women (test strip)**

- **Immunology:**
  - CD4 lymphocytes count
  - CD4 lymphocytes percentage
  - CD8 lymphocytes count
  - CD8 lymphocytes percentage

Note: Before the beginning of the study, all labs will facilitate to the sponsor and to the investigator a list of the reference normal values of the parameters assessed.

- **Specific Lab Procedures**

To be assessed along the study, as detailed in the endpoint section 5.3:

- Romidepsin concentrations in plasma will be determined by liquid chromatography–mass spectrometry (LC-MS) / mass spectrometry (MS)
- HIV-1 DNA in CD4 T cells by ddPCR
- Cell-associated RNA (CA-RNA)

- Viability, necrosis and apoptosis levels by flow cytometry
- Activation, exhaustion and proliferation capacity by flow cytometry
- IFNg production assessed by ELISPOT
- In-vitro viral inhibition capacity
- Ultrasenstive plasma viral load (SCA)
- Histone H3 acetylation in lymphocytes

8.4.3 Electrocardiogram

Electrocardiogram changes have been described in trials using romidepsin at oncologic doses. EKG leads are attached to the body while the patient lies flat on a bed. Leads are attached to each extremity (four total) and to six pre-defined positions on the front of the chest. A small amount of gel is applied to the skin, which allows the electrical impulses of the heart to be more easily transmitted to the EKG leads. The leads are attached by small suction cups, Velcro straps, or by small adhesive patches attached loosely to the skin. The test takes about five minutes and is painless. During $RMD_{1,2,3}$ infusions, ECG will be performed to estimate heart rate and detect potential QT prolongation, repolarization abnormalities and/or T wave inversion.

8.4.4 Vaccine patient diary

Data on local and systemic events will be solicited with specific diary cards (attachement F) for a minimum of 7 days following each immunization. Following parameters will be collected:

Local Reactogenicity, including:

- Redness (mm)
- Induration (mm)
- Pain (0-4)

General Symptoms, including:

- Temperature (ºC)
- Headache (0-4)
- Fatigue (0-4)
- Nausa (0-4)
- Vomiting (0-4)
- Diarrhea (0-4)
- Abdominal pain (0-4)
- Sudoration (0-4)
- Muscular pain (0-4)
- Lack of appetite (0-4)
- Other, describe

0-4 grades intensity, according to the following definitions described at DAIDS:

- 0: No symptoms
- 1: Symptoms causing no or minimal interference with usual social & functional activities.

ANNEX 3, OP.02.01.FLS-RS.02. Pre-estudi 14/10/2014

- 2: Symptoms causing greater than minimal interference with usual social & functional activities
- 3: Symptoms causing inability to perform usual social & functional activities
- 4: Symptoms causing inability to perform basic self-care functions OR Medical or operative intervention indicated to prevent permanent impairment, persistent disability, or death

The investigator will review the patient diary and ensure that proper record has been performed. The severity of each AE will be graded by the investigator. Criteria for grading clinical and laboratory events were based on DAIDS grading table. The investigator will state the relationship of each AE to the vaccination by using prespecified terms including unrelated, unlikely to be, possibly, probably and definitely related to vaccination. In those cases were grading of AE do not coincide with patient's decription, a discussion will be registered in the medical record.

ANNEX 3, OP.02.01.FLS-RS.02. Pre-estudi 14/10/2014

8.4.5 Romidepsin patient diary

Data on local and systemic events will be solicited with specific diary cards (attachment G) for a minimum of 7 days following each romidepsin administration. Following parameters will be collected:

General Symptoms, including:

- Temperature (ºC)
- Headache (0-4)
- Fatigue (0-4)
- Nausa (0-4)
- Vomiting (0-4)
- Diarrhea (0-4)
- Abdominal pain (0-4)
- Sudoration (0-4)
- Muscular pain (0-4)
- Lack of appetite (0-4)
- Heart palpitations (0-4)
- Rash (0-4)
- Bloating (0-3)
- Metallic Taste (0-4)
- Other, describe

0-4 grades intensity, according to the following definitions:

- 0: No symptoms
- 1: Symptoms causing no or minimal interference with usual social & functional activities
- 2: Symptoms causing greater than minimal interference with usual social & functional activities
- 3: Symptoms causing inability to perform usual social & functional activities
- 4: Symptoms causing inability to perform basic self-care functions OR Medical or operative intervention indicated to prevent permanent impairment, persistent disability, or death

The investigator will review the patient diary and ensure that proper record has been performed. The severity of each AE will be graded by the investigator. Criteria for grading clinical and laboratory events were based on DAIDS scale. The investigator will state the relationship of each AE to the vaccination by using prespecified terms including unrelated, unlikely to be, possibly, probably and definitely related to vaccination. In those cases were grading of AE do not coincide with patient's decription, a discussion will be registered in the medical record.

ANNEX 3, OP.02.01.FLS-RS.02. Pre-estudi 14/10/2014

ANNEX 3, OP.02.01.FLS-RS.02. Pre-estudi 14/10/2014

9 ADVERSE EVENTS

9.1 DEFINITION

Adverse event: (AE) Medical event presented by a patient or clinical research subject administered a pharmaceutical product, and which does not necessarily have a causal relation to the treatment.

Serious adverse event: (SAE) Medical event classified as such and which, regardless of the dose involved:

- causes patient death,
- produces a life-threatening situation for the patient,
- requires or prolongs in hospital admission,
- produces important or persistent incapacitation/handicap, or constitutes a congenital defect or anomaly,
- needs action to prevent any of above situations.
- is considered medically significant

Examples of such events are intensive care in an Emergency Service or in the home in a patient with allergic bronchospasm; blood dyscrasias or seizures not giving rise to hospital admission, or the development of drug dependency or abuse.

Unexpected adverse event: (UAE) AE related to the product in investigation the nature or intensity of which does not coincide with the information available on the product administered (IB or SmPC).

Serious unexpected adverse reaction: (SUSAR) SAE related to the product in investigation the nature or intensity of which does not coincide with the information available on the product administered (IB or SmPC).

9.2 MONITORING, RECORDING AND REPORTING OF ADVERSE EVENTS

An AE is any noxious, unintended, or untoward medical occurrence that may appear or worsen in a subject during the course of a study. It may be a new intercurrent illness, a worsening concomitant illness, an injury, or any concomitant impairment of the subject’s health, including laboratory test values (as specified by the criteria below), regardless of etiology. Any worsening (i.e., any clinically significant adverse change in the frequency or intensity of a pre-existing condition) should be considered an AE. A diagnosis or syndrome should be recorded on the AE page of the CRF rather than the individual signs or symptoms of the diagnosis or syndrome.

An overdose, accidental or intentional, whether or not it is associated with an AE, or abuse, withdrawal, sensitivity or toxicity to an investigational product should be reported as an AE. If an overdose is associated with an AE, the overdose and AE should be reported as separate terms.

ANNEX 3, OP.02.01.FLS-RS.02. Pre-estudi 14/10/2014

All subjects will be monitored for AEs during the study. Assessments may include monitoring of any or all of the following parameters: the subject's clinical symptoms, laboratory, pathological, radiological or surgical findings, physical examination findings, or other appropriate tests and procedures.

All AEs will be recorded by the Investigator from the time the subject signs informed consent until the last study visit or to 28 days after the last dose of IMP in case of early withdrawal while patient is receiving IMP. AEs and SAEs will be recorded on the AE page of the CRF and in the subject's source documents. All SAEs must be reported to the Sponsor (see contact details at the end of the paragraph) within 24 hours of the Investigator's knowledge of the event by facsimile, or other appropriate method, using the SAE Report Form (attachment N, can be found in the Investigator's File), or approved equivalent form.

9.3 DOCUMENTATION RELATED TO AE AND SAE

Each AE and SAE to take place during the study should be documented in the medical records of the patient in accordance with standard clinical practice of the researcher, and in the CRF. For each SAE, an independent set of SAE form will be used independently. Only if there are multiple SAE at the time of the initial report and these are temporary and / or clinically interrelated can be registered on the same set of SAE form.

The researcher should try to make a diagnosis of the event based on the signs, symptoms and / or other clinical information. An AE diagnosis has to be recorded per line or a sign/symptom if the diagnosis is not available. If a diagnosis subsequently becomes available, this then should be entered and the sign/symptom crossed out, initialed and dated by the investigator.

SAE pages found in the investigator's file shall be completed as precisely as possible, printed and shall be signed by the investigator before being sent to the sponsor. It is very important that the initial page SAE researcher provide its opinion in regard to the relationship of the event to the study drug.

9.4 EVALUATION OF ADVERSE EVENTS

A qualified Investigator will evaluate all AEs as to:

9.4.1 Seriousness

A SAE is any AE occurring at any dose that:

- Results in death;
- Is life-threatening (i.e., in the opinion of the Investigator, the subject is at immediate risk of death from the AE);
- Requires inpatient hospitalization or prolongation of existing hospitalization (hospitalization is defined as an inpatient admission, regardless of length of stay);

- Results in persistent or significant disability/incapacity (a substantial disruption of the subject's ability to conduct normal life functions);
- Is a congenital anomaly/birth defect;
- Constitutes an important medical event.

Important medical events are defined as those occurrences that may not be immediately life threatening or result in death, hospitalization, or disability, but may jeopardize the subject or require medical or surgical intervention to prevent one of the other outcomes listed above. Medical and scientific judgment should be exercised in deciding whether such an AE should be considered serious.

Events **not considered** to be SAEs are hospitalizations for:

- A standard procedure for protocol therapy administration. However, hospitalization or prolonged hospitalization for a complication of therapy administration will be reported as an SAE.
- Routine treatment or monitoring of the studied indication not associated with any deterioration in condition.
- A procedure for protocol/disease-related investigations (e.g., surgery, scans, endoscopy, sampling for laboratory tests, bone marrow sampling). However, hospitalization or prolonged hospitalization for a complication of such procedures remains a reportable SAE.
- Hospitalization or prolongation of hospitalization for technical, practical, or social reasons, in absence of an AE.
- A procedure that is planned (i.e., planned prior to starting of treatment on study); must be documented in the source document and the CRF. Hospitalization or prolonged hospitalization for a complication remains a reportable SAE.
- An elective treatment of a pre-existing condition unrelated to the studied indication.
- Emergency outpatient treatment or observation that does not result in admission, unless fulfilling other seriousness criteria above.

If an AE is considered serious, both the AE page/screen of the CRF and the SAE Report Form must be completed.

For each SAE, the Investigator will provide information on severity, start and stop dates, relationship to IP, action taken regarding IP, and outcome.

ANNEX 3, OP.02.01.FLS-RS.02. Pre-estudi 14/10/2014

ANNEX 3, OP.02.01.FLS-RS.02. Pre-estudi 14/10/2014

9.4.2 Severity / Intensity

For both AEs and SAEs, the Investigator must assess the severity / intensity of the event.

Intensity will be assessed by the investigator using the following terms according to the Division of AIDS table for grading the severity of adult and pediatric adverse events (Attachment O):

- Grade 1 = Mild
- Grade 2 = Moderate
- Grade 3 = Severe
- Grade 4 = Life threatening
- Grade 5 = Death

The term "severe" is often used to describe the intensity of a specific event (as in mild, moderate or severe myocardial infarction); the event itself, however, may be of relatively minor medical significance (such as severe headache). This criterion is *not* the same as "serious" which is based on subject/event *outcome* or *action* criteria associated with events that pose a threat to a subject's life or functioning.

Seriousness, not severity, serves as a guide for defining regulatory obligations.

9.4.3 Causality

The causal relation will be established according to the algorithm of the Spanish Pharmacovigilance System, which contemplates the following categories:

Definitive:

- A plausible time sequence exists in relation to administration of the drug or its plasma or tissue concentrations.
- The observed manifestation coincides with the known adverse reactions profile of the implicated drug.
- The event cannot be explained by the concurrent disease or by other drugs or chemical substances.
- Response to withdrawal must be clinically plausible, i.e., the condition improves on discontinuing administration of the drug.
- A positive response to repeat exposure is observed.

Probable:

- A reasonable time sequence exists in relation to administration of the drug.
- The observed manifestation coincides with the known adverse reactions profile of the implicated drug.
- The event is unlikely to be explained by the concurrent disease or by other drugs or chemical substances.

- Response to withdrawal is clinically plausible, i.e., the condition improves on discontinuing administration of the drug.
- No repeat exposure is required to complete this definition.

Possible:
- A reasonable time sequence exists in relation to administration of the drug.
- The observed manifestation coincides with the known adverse reactions profile of the implicated drug.
- The event might be attributable to the clinical condition of the patient or to other concomitantly administered drugs or chemical substances.
- Information concerning drug withdrawal may be unavailable or confusing.

Improbable:
- A clinical event, including anomalous laboratory test findings, with a time relation to administration of the drug which makes a causal association unlikely, and where other drugs, chemical substances or intercurrent disease afford plausible explanations for the observed event.

Unrelated:
- None of the above criteria are met.

9.4.4 Duration

For both AEs and SAEs, the Investigator will provide a record of the start and stop dates of the event.

9.4.5 Action Taken

The Investigator will report the action taken with IMP as a result of an AE or SAE, as applicable (e.g., discontinuation or reduction of IMP, as appropriate) and report if concomitant and/or additional treatments were given for the event.

9.4.6 Outcome

SAE will be followed preferably until:
- Resolution of the event;
- Stabilization of the event; or
- Resetting the baseline situation of the event, in case baseline situation is available.

Otherwise, they will continue until:
- The event can be attributed to products other than the study medication or factors unrelated to the study; or
- It is unlikely to obtain further information.

ANNEX 3, OP.02.01.FLS-RS.02. Pre-estudi 14/10/2014

ANNEX 3, OP.02.01.FLS-RS.02. Pre-estudi 14/10/2014

9.5 ABNORMAL LABORATORY VALUES

An abnormal laboratory value is considered to be an AE if the abnormality:

- results in discontinuation from the study;
- requires treatment, modification/ interruption of IP dose, or any other therapeutic intervention; or
- is judged to be of significant clinical importance.

Regardless of severity grade, only laboratory abnormalities that fulfill a seriousness criterion need to be documented as a serious adverse event.

If a laboratory abnormality is one component of a diagnosis or syndrome, then only the diagnosis or syndrome should be recorded on the AE page/screen of the CRF. If the abnormality was not a part of a diagnosis or syndrome, then the laboratory abnormality should be recorded as the AE.

9.6 FEMALES OF CHILDBEARING POTENTIAL

Pregnancies and suspected pregnancies (including a positive pregnancy test regardless of age or disease state) of a female subject occurring while the subject is on romidepsin or MVA.HIVconsv, or within 28 days of the subject's last dose of romidepsin or MVA.HIVconsv, are considered immediately reportable events. Romidepsin or MVA.HIVconsv is to be discontinued immediately and the subject instructed to return any unused portion of romidepsin or MVA.HIVconsv to the Investigator. The pregnancy, suspected pregnancy, or positive pregnancy test must be reported to the Sponsor who, in case patient was exposed to romidepsin, will inform Celgene immediately using the Pregnancy Reporting Form provided by Celgene or an approved equivalent form.

Romidepsin may have reproductive toxicity if taken during pregnancy. In case the female subject was exposed to romidepsin, she should be referred to an obstetrician-gynecologist, preferably one experienced in reproductive toxicity for further evaluation and counseling.

The Investigator will follow the female subject until completion of the pregnancy, and must notify the Sponsor immediately about the outcome of the pregnancy (either normal or abnormal outcome).

If the outcome of the pregnancy was abnormal (e.g., spontaneous or therapeutic abortion), the Investigator should report the abnormal outcome as an AE. If the abnormal outcome meets any of the serious criteria, it must be reported as an SAE within 24 hours of the Investigator's knowledge of the event using the SAE Report Form, or approved equivalent form.

All neonatal deaths that occur within 28 days of birth should be reported, without regard to causality, as SAEs. In addition, any infant death after 28 days that the Investigator suspects is related to the in utero exposure to the IP should also be reported within 24

hours of the Investigator's knowledge of the event using the SAE Report Form, or approved equivalent form.

9.6.1 Male Subjects

The investigator shall use their best efforts in order to ensure that male patients

- inform him/her if their partners get pregnant while the Patient is still treated with the IMP;
- provide him/her the contact details of the healthcare provider who follows the pregnancy in question.

If a female partner of a male subject taking the IMP becomes pregnant, the investigator shall:

- advise that the partner consults her general practitioner or gynecologist as soon as possible;
- (in case romidepsin exposure) provide the information collected to Celgene's Drug Safety Department, using the Pregnancy Reporting Form or an approved equivalent form.

9.7 SCIENTIFIC COMMITTEE

A Scientific committee will be formed to review clinical data and stopping rules. The committee will be composed of the coordinating investigator plus two physicians of the investigator team and one external physician. See attachment P for details

9.8 EXPEDITED REPORTING OF ADVERSE EVENTS

9.8.1 Reporting to Regulatory Authorities and the Ethics Committee

The sponsor will inform the Spanish Medicines Agency (Ministry of Health), the competent authorities of the autonomous region and the Ethics Committees implicated in the clinical trial about any important information of security of the IMPs.

The sponsor will inform the Spanish Medicines Agency (Ministry of Health) of any SUSAR which may be related to the study treatment.

The sponsor will inform competent authorities of the implicated autonomous region of any SUSAR which may be related to the study treatment, and that have been happened in patients in its autonomous region.

ANNEX 3, OP.02.01.FLS-RS.02. Pre-estudi 14/10/2014

The sponsor will inform the Ethics Committee of any SUSAR which may be related to the study treatment, and that have been happened in patients included in its sphere of action.

The Sponsor will inform relevant Regulatory Authorities and Ethics Committees;

- Of all relevant information about SUSAR that are fatal or life-threatening as soon as possible, and in any case no later than 7 days after knowledge of such a case. Relevant follow-up information for these cases will be subsequently be submitted within an additional eight days
- Of all other SUSAR as soon as possible, but within a maximum of 15 days of first knowledge by the investigator.

9.8.2 Immediate reporting by Investigator to Sponsor

The investigator will inform the Sponsor of all SAEs within 24 hours in order that the sponsor can fulfill their regulatory reporting obligations within the required timeframes.

Contact details for Sponsor
Roser Escrig Sarreta / Sílvia Gel Moretó
rescrig@fls-rs.com / sgel@fls-rs.com
Phone: +34 93 497 84 14, Fax. +34 93 465 76 02

9.8.3 Reporting by Sponsor to Celgene

The Sponsor will supply Celgene with a copy of all SAEs which involve exposure to romidepsin within 24 hours of being made aware of the event regardless of whether or not the event is listed in the reference document (e.g. IB, SmPC).

The Sponsor will provide Celgene with a copy of the annual periodic safety report e.g. Development Update Safety Report (DSUR) at the time of submission to the Regulatory Authority and Ethics Committee.

Contact details for Celgene
Celgene Drug Safety Department
Phone: +34 630 56 48 73, Fax: +34 91 422 90 95
Email: drugsafety-spain@celgene.com

9.8.4 Reporting by Sponsor to Coordinating investigator

The Sponsor will inform the Coordinating investigator of all SAEs within 24 hours in order that the Coordinating investigator can call the scientific committee to assess trial stopping rules.

Contact details for Coordinating investigator
Beatriz Mothe Pujadas
Phone: +34 934 656 374 (Ext 165), Fax: +34 934 653 968
Email: bmothe@irsicaixa.es

ANNEX 3, OP.02.01.FLS-RS.02. Pre-estudi 14/10/2014

ANNEX 3, OP.02.01.FLS-RS.02. Pre-estudi 14/10/2014

10 STATISTICS

The primary safety and tolerability analysis will include a description and summary of the number and percentage of grade ≥3 AE and SAE; their grading and imputability for both MVA.HIVconsv and RMD administrations.

10.1 RESPONSE EVALUATION (EFFICACY)

Two main efficacy endpoints will be evaluated in the BCN02-trial as follows:

Part A) Efficacy of the combined intervention (Kick & kill strategy) in the reduction of viral reservoir will be expressed by total HIV-1 DNA copies per million purified CD4+ T cells measured by quantitative digital droplet PCR (ddPCR) at baseline and 1 week after 3rd dose of RMD. Wilcoxon signed rank test will be used for comparisons.

Part B) Efficacy of HIVconsv vaccines in combination with RMD on viral rebound containment after treatment interruption will be evaluated by analysing the proportion of individuals who maintain sustained pVL <2,000 copies/ml at week 29 (12weeks after treatment cessation). Complementary, a survival analysis will be performed to describe time to rebound during the MAP period. Taking into consideration recent published data suggesting that early treatment initiation could favor a delayed viral rebound/spontaneous viral control in 10% in opposition to <5% in chronically-treated individuals, a positive efficacy signal has been established if at least >20% of individuals remain with pVL <2,000 copies/ml at w12 of MAP (pre-defined efficacy threshold).

Regarding virological and immunogenicity endpoints: Differences in the breadth and magnitude of different virological and immune parameters analyses between two longitudinal determinations in the same individuals will be assessed using a Wilcoxon signed-rank test. Correlations between the virologic, immune parameters and clinical outcome will be performed using Pearson test.

Population PK/PD analysis: Briefly, a pharmacokinetic model will be developed using a population approach by means of the software NONMEM 7.3. The population approach allows us, trough an intensive modelling exercise, to analyze the data from all participants at the same time but keeping their individual origin. Therefore, estimates for the pharmacokinetic parameters describing not only the mean tendency of the concentration-time course in the population but also at the individual levels will be obtained. The main steps to develop the proposed population pharmacokinetic model are: 1) The pharmacokinetic profile of RMD using the data from the experimental phase of the project will be established: one, two or three compartment models to describe RMD pharmacokinetics will be assessed; 2) Interindividual variability in RMD pharmacokinetic parameters as well as interoccasion variability will be determined, and; 3) The influence of individual characteristics (covariates) in RMD pharmacokinetic parameters will be incorporated into the model. This process will consist of two steps: *forward inclusion* and *backward elimination*. Statistical significance cut-offs for retaining a covariate into the model during the *forward inclusion* and the *backward elimination* steps will be 0.05 and 0.01, respectively. In addition, those covariates without clinical

plausibility will be removed from the model. Once the model had been developed, its predictive capacity will be evaluated by means of simulation and resampling methods (Monte Carlo and bootstrapping, respectively). The final model will be used to evaluate the relationship between RMD exposure and the magnitude of either inhibition of hystone deacetilation, HIV reactivation and CTL. Full RMD pharmacokinetic profiles after each RMD dose will be simulated, and area under the curve, maximum and minimum RMD concentrations in plasma will be determined at each occasion and for each participant. A PK/PD analysis will be carried out to evaluate the relationship between each PK parameter and each pharmacodynamic endpoint (lineal, exponential or maximum effects models will be tested). The results from this analysis will be used to determine, trough simulation exercises, the dose of RMD to be given to each particular patient to maximize the chances to get the desired effect with minimum risk of deleterious effects.

Correlates of control: By multivariate regression techniques and integration of omics data, biological and clinical markers that predict the effect in the control of viral rebound after the MAP will be analyzed. Both the identification of predictors and the calculation of predictive power of the model will be performed within a process of "internal validation" using resampling techniques (bootstrapping and cross-validation).

10.2 SECURITY ANALYSIS

In addition to safety description analysis, stopping rules will be established for the two parts of the study as follows:

a) Stopping rules during the Part A of the study will be based on the observation of an unexpected high proportion of individuals experiencing any AE/SAE to any of the vaccinations and/or RMD administration as described in section 5.6.

b) On the other side and in order to minimize risks of the intervention, an early interim and futility analyses is planned for each individual before the MAP phase. Acceptability for cART interruption after interim analyses will be reviewed. Treatment interruption would be discouraged individually if a) boosting MVA.HIVconsv was shown to be no longer immunogenic after RMD administration (no net increase in induced response compared to pre-vaccination levels with MVA.HIVconsv at BCN02-Romi baseline visit) and/or b) persistent detectable viremia. Limited MVA.HIVconsv immunogenicity after MVA2 may indicate RMD toxicity on CTL functional activity for instance; therefore, an improved viral control would not be foreseen. On the other hand, increases in immune activation shortly after immunization are expected to decrease 8 weeks after vaccination. However, in some cases, intermittent detectable plasma low-level viremia have also been reported to occur after vaccination, which may induce an extended immune activated status that could favor a faster and higher viral rebound after treatment cessation. Therefore, clinical team will discuss cases where treatment interruption could be also discouraged in cases with persistent detectable viremia (i.e. longer than 12 weeks after last immunization)

ANNEX 3, OP.02.01.FLS-RS.02. Pre-estudi 14/10/2014

In all cases where immune and/or virological futility criteria are not met and treatment interruption is discouraged, trial results would be focused on the viral reservoir and immunogenicity endpoints only until better correlates of control would be available to predict clinically relevant outcomes on which one could recommend treatment interruptions in a safer manner.

10.3 SAMPLE SIZE DESCRIPTION

Individuals that participated in the BCN01 trial and which are followed in the extension phase (BCN01-RO) will be offered to participate in the study. Therefore, the trial will enroll a maximum number of 24 subjects. The primary endpoint of the study is safety and tolerability of the combined kick & kill strategy in recently HIV-1-infected individuals under early suppressive therapy. In terms of total number of individuals exposed to each MVA.HIVconsv vaccines and confidence intervals to detect an AE (i-e- based on published results the probability of experiencing a grade 3 or 4 AE is thought to be <0.1). Information on expected rates of AEs related to RMD at the clinical dose proposed is still scarce. The trial has been designed as a pilot study and will only allow detection of a large effect among participants. However, the incidence of grade $\geq$ 3 or SAEs will be used as a measure of safety of the experimental strategy.

10.4 DEVIATION OF STATISTICAL PLAN

Any deviation from that presented statistical plan will be described and justified in the final report.

ANNEX 3, OP.02.01.FLS-RS.02. Pre-estudi 14/10/2014

11 DIRECT ACCESS TO SOURCE DATA / DOCUMENTS

Researchers and institutions will allow the monitoring, and audits by the Health Authorities or the Sponsor giving direct access to data and original source documents.

Access to personal patient information will be restricted to the Study physician / staff. To allow monitorings, audits and inspections, access to data to Health Authorities (Spanish Agency for Medicines and Health Products), the Ethics Committee and personnel authorized by the Sponsor, is guaranteed while maintaining the confidentiality thereof according to current legislation.

ANNEX 3, OP.02.01.FLS-RS.02. Pre-estudi 14/10/2014

12 QUALITY CONTROL AND QUALITY ASSURANCE

12.1 STUDY MONITORING

In accordance with applicable regulations and Good Clinical Practice (GCP), the monitor will visit or contact the center on a regular basis. The duration, nature and frequency of visits / contacts depend on the monitoring plan.

During these contacts, the monitor shall:

- monitor and evaluate the progress of the study;
- examining the data collected;
- carry out a verification of the source documents;
- identify any problems and find solutions;

The goal of the monitoring activity is to verify that:

- the rights and welfare of subjects are respected;
- survey data are accurate, complete and verifiable with the help of original documents;
- the study is performed according to the protocol and any amendment adopted, GCPs and regulations.

The investigator must agree to:

- grant to monitor direct access to all relevant documentation;
- devote part of his/her time and staff time to the monitor in order to discuss the results of the monitoring, as well as any other possible aspect.

The monitor should also contact the center before starting the study with the aim to discuss with staff the Protocol and procedures for data collection.

12.2 AUDITS AND INSPECTIONS

Sponsor can carry out an audit of quality control at its sole discretion. In this case, the investigator should agree to grant the auditor direct access to all relevant documentation and devote part of his/her time and staff time to the auditor in order to discuss the results of the monitoring, as well as any other possible aspect.

Moreover, regulatory authorities may also inspect the study. In this case, the investigator should agree to give the inspector direct access to all relevant documentation and devote part of his/her time and staff time to the inspector in order to discuss the results of the supervision, as well as any other possible aspect.

ANNEX 3, OP.02.01.FLS-RS.02. Pre-estudi 14/10/2014

12.3 CASE REPORT FORM

Data collection will be done through an electronic CRF with a system of access by username and password. The application has track changes (recording the user that has performed).

Accurate and reliable data collection is ensured by checking and cross checking the CRF front site records conducted by the study monitor (verification of source documents). The data collected will be added to a computer database which will be reviewed for possible inconsistencies to be resolved by the research team of the study in each site.

The content of the CRF is attached in Attachment Q

ANNEX 3, OP.02.01.FLS-RS.02. Pre-estudi 14/10/2014

13 ETHICS

13.1 GENERAL CONSIDERATIONS

The clinical trial will be conducted according to the principles of the Declaration of Helsinki, Fortaleza, Brasil, octubre 2013.

This study will be conducted according to Spanish regulations and the required documentation prior to the start will be:

- Protocol acceptance by the sponsor and the coordinating investigator
- Protocol approval by the Ethics Committee.
- Protocol authorization from the Spanish Drug Agency (Ministry of Health)

All subjects will be guaranteed continued medical and nursing supervision throughout the duration of the study.

This study will conform to the standards of "Good Clinical Practice".

Also, following the "Good participatory practice guidelines" (published by the Joint United Nations Programme on HIV/AIDS (UNAIDS)), representatives of the community-based detection centres have participated in the design of the protocol and the Participant Information Sheet, through the Community Advisory Committee (CAC).

13.2 PATIENT INFORMATION SHEET AND INFORMED CONSENT

Informed consent will be obtained before including the patient in the trial. The investigator is to inform the patient of the nature, duration and purpose of the study, as well as of all the obstacles and inconveniences which – within reason – may be expected from it. Furthermore, the patient is to receive information in writing. The participating patients must be legally competent to give informed consent, with the possibility of taking decisions at his/her own free will. The patient has the right to leave the study at any time.

Patient information sheet (PIS) and informed consent form (ICF) may be found in attachment R.

13.3 PAYMENT TO RESEARCH SUBJECTS

Volunteers will be reimbursed for their time, effort and time travels to study sites due to study participation. Reimbursement amounts will be documented in the PIS.

A payment of € 500 for meals, transfers and lost in productivity is foreseen and will be set in two times:

- € 200 at the end of first phase
- € 300 at the end of the second phase

ANNEX 3, OP.02.01.FLS-RS.02. Pre-estudi 14/10/2014

Additionally, a free taxi service will be offered to patients for vaccination and romidepsin administration visits performed in UPIC. Particularly, service will be offered for journeys to Hospital Universitari Germans Trias I Pujol (HGTIP)in visits MVA_1, RMD_1, RMD_2 RMD_3 and MVA_2 and return journeys in visits RMD_2 and RMD_3.

14 DATA HANDLING AND RECORD KEEPING

14.1 DATA HANDLING

The processing of the data to be compiled by the study sponsor during the trial will be subject to current legislation as regards data protection (LOPD, Ley Orgánica 15/1999, de 13 de diciembre de protección de datos de carácter personal). The patient will be identified in the records by the corresponding code number only. The patient is to be guaranteed anonymity, and is to be informed that all communication will take place between him/her and the investigator – not the sponsor of the trial.

Data transmitted to third countries and other countries will in no case contain personal data. In the event that such transfer occurs, it will be for the same purposes of the study described and ensuring confidentiality at least to the level of protection of the law in Spain.

14.2 RECORD KEEPING

14.2.1 Investigator file and document retention

The investigator must keep the investigator file with the proper and accurate records to enable the study to be fully documented and data subsequently verified.

The Investigator's study file will contain the protocol and its amendments, CRFs, questionnaires' forms, EC approval and authorization from the health authorities, samples of the patient information sheet and informed consent, staff curriculum, signatures' delegation log and listing of subjects, as well as other appropriate documents and correspondence.

Clinical source documents from subjects (usually predefined by the project to record key efficacy and safety parameters or documents that are not in the clinical record of the hospital) will be filed indicating the number of patient without personal data.

The investigator should retain these documents at least five years, according to SCO/ 256/2007, provided that the promoter does not express a greater period.

14.2.2 Source documents and basic data

The information contained in the CRF will be considered as primary data, except for patient filiation data and lab tests. Patient participation in the study will be collected on medical records, including assigned code number and identification of the different study

ANNEX 3, OP.02.01.FLS-RS.02. Pre-estudi 14/10/2014

visits that will take place throughout the study. At the end of the study, a copy of the CRF will be placed on the site.

ANNEX 3, OP.02.01.FLS-RS.02. Pre-estudi 14/10/2014

15 FINANCING AND INSURANCE

15.1 SOURCE OF FINANCING

The study will be funded partially by a competitive grant (PI15/01188), Lluita contra la SIDA Foundation and IrsiCaixa AIDS Research Institute – HIVACAT (Sponsor)

15.2 INSURANCE POLICY

In accordance with Article 8 of Royal Decree 223/2004, of 6 February, the trial sponsor has a policy of liability insurance with Zurich Insurance Company PLC Branch in Spain established in Barcelona. The sponsor shall extend this policy or another with equivalent coverage until the end of the trial. The policy will cover the damages to the people that could be set as a result of the trial by an insured amount of 300,000 € per patient tested to a maximum of € 3,000,000 per year and clinical trial. This policy also covers the responsibilities of the sponsor, the principal and his/her collaborators, as well as the hospital or site where they carry out the clinical trial.

The sponsor agrees to pay the premiums to cover the liability pertaining to the trial. It is presumed, unless proven otherwise, that damage affecting the health of the person subject to testing during implementation and in the following year the completion of treatment, have occurred as a result of the trial. However, once the year ended, the test subject is required to prove the link between the trial and damage.

The site and the principal investigator undertake to inform the sponsor of any claim or legal, real or potential action if known, linkable to trial.

Insurance policy may be found in attachment S.

ANNEX 3, OP.02.01.FLS-RS.02. Pre-estudi 14/10/2014

16 PUBLICATION POLICY

The publication of the trial results shall meet the requirements set out in Article 38 of Royal Decree 223/2004.

ANNEX 3, OP.02.01.FLS-RS.02. Pre-estudi 14/10/2014

ATTACHMENT A: STUDY DIAGRAM

ANNEX 3, OP.02.01.FLS-RS.02. Pre-estudi 14/10/2014

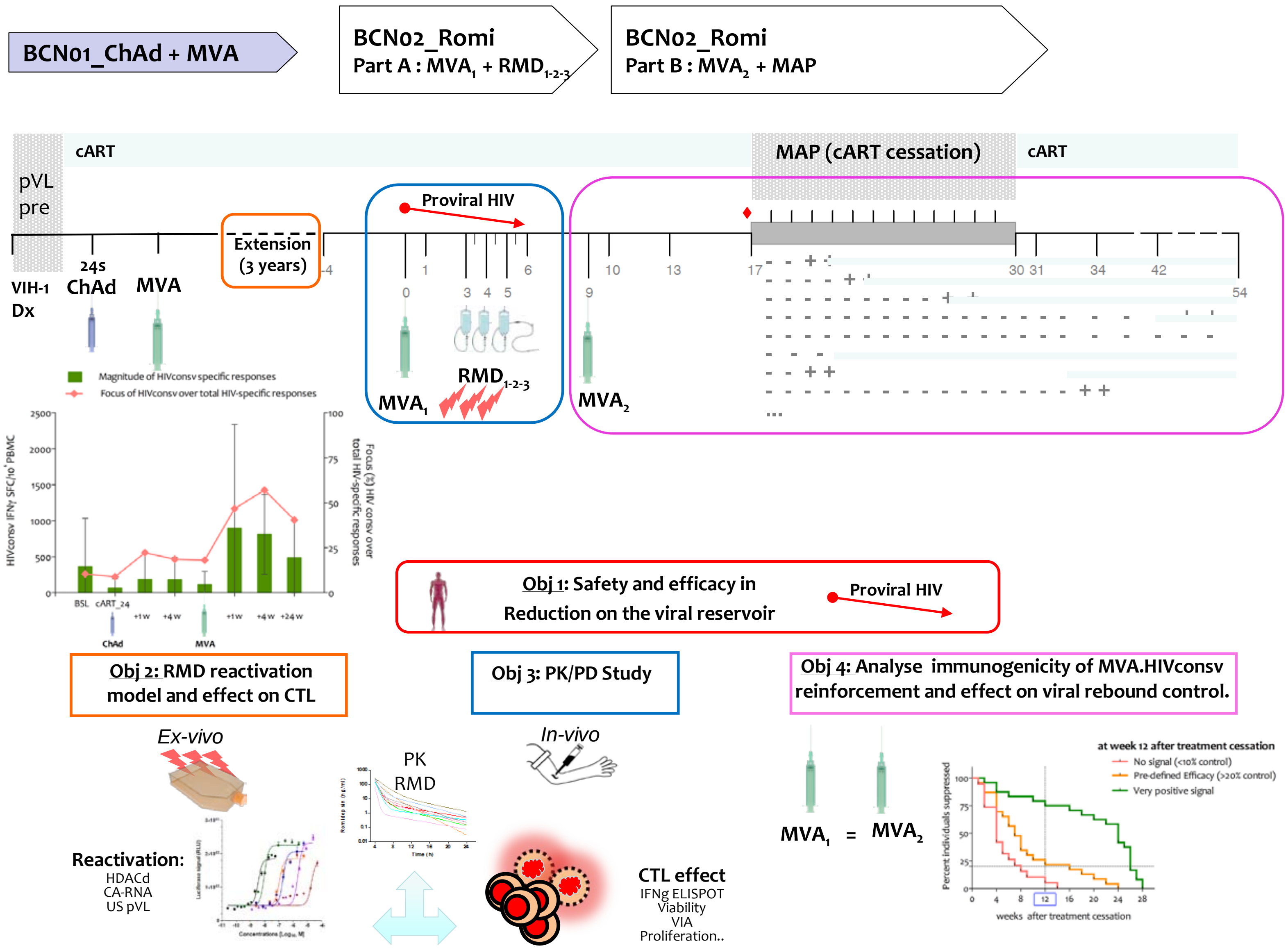

BCN01_ChAd + MVA
BCN02_Romi
Part A : MVA1 + RMD1-2-3
BCN02_Romi
Part B : MVA2 + MAP
cART
MAP (cART cessation)
cART
pVL
pre
Proviral HIV
Extension
(3 years)
VIH-1
Dx
24s
ChAd
MVA
MVA1
RMD1-2-3
MVA2
Magnitude of HIVconsv specific responses
Focus of HIVconsv over total HIV-specific responses
Obj 1: Safety and efficacy in
Reduction on the viral reservoir
Proviral HIV
Obj 2: RMD reactivation
model and effect on CTL
Obj 3: PK/PD Study
Obj 4: Analyse immunogenicity of MVA.HIVconsv
reinforcement and effect on viral rebound control.
Ex-vivo
PK
RMD
In-vivo
Reactivation:
HDACd
CA-RNA
US pVL
CTL effect
IFNg ELISPOT
Viability
VIA
Proliferation..
MVA1 = MVA2
at week 12 after treatment cessation
No signal (<10% control)
Pre-defined Efficacy (>20% control)
Very positive signal
weeks after treatment cessation

ATTACHMENT B: ROMIDEPSIN ADMINISTRATION FORM

(attached)

ATTACHMENT C: MVA.HIVCONSV ADMINISTRATION FORM

(attached)

ATTACHMENT D: ADAPTED SERAD QUESTIONAIRE

FORMULARIO DE ADHERENCIA SERAD

ID Paciente: __ __ __ __ __ __ __ __ __

Fecha: __ __ / __ __ / __ __

Indicar el total de pastillas diarias prescritas de cada antirretroviral y el número de pastillas olvidadas la última semana y el último mes.

Tratamiento antirretroviral	Núm. de pastillas que debe tomar cada día		Núm. de pastillas que ha olvidado durante la última semana		Núm. de pastillas que ha olvidado durante el último mes	
	Mañana	Tarde	Mañana	Tarde	Mañana	Tarde

Adherencia a la semana: _______ %

Adherencia al mes: ________ %

ATTACHMENT E: MAP PHASE MONITORING FORM

(attached)

ATTACHMENT F: MVA DIARY CARD

(attached)

ATTACHMENT G: ROMIDEPSIN DIARY CARD

(attached)

ATTACHMENT H: SOP FOR ROMIDEPSIN HANDLING AND ADMINISTRATION

(attached)

ATTACHMENT I: SOP FOR MVA.HIVCONSV HANDLING AND ADMINISTRATION

(attached)

ATTACHMENT J: SCHEDULE OF PROCEDURES

(attached)

ATTACHMENT K: SCHEME FOR PBMC/PLASMA STORAGE

(attached)

ATTACHMENT L: MVA.HIVCONSV PRESCRIPTION FORM

(attached)

ATTACHMENT M: MAP DECISIONS CHART

(attached)

ATTACHMENT N: SAE/PREGNANCY NOTIFICATION INSTRUCTIONS AND FORM

(attached)

ANNEX 3, OP.02.01.FLS-RS.02. Pre-estudi 14/10/2014

ATTACHMENT O: DIVISION OF AIDS TABLE FOR GRADING THE SEVERITY OF ADULT AND PEDIATRIC ADVERSE EVENTS

(attached)

ATTACHMENT P: SCIENTIFIC COMMITTEE

(attached)

ATTACHMENT Q: CRF CONTENT

(attached)

ATTACHMENT R: PATIENT INFORMATION AND WRITTEN INFORMED CONSENT

(attached)

ATTACHMENT S: INSURANCE

(attached)

ATTACHMENT T: ROMIDEPSIN INVESTIGATOR BROCHURE

(attached)

ATTACHMENT U: MVA.HIVCONSV INVESTIGATOR BROCHURE

(attached)

ATTACHMENT V: STUDY STAFF

(attached)

ATTACHMENT W: PK FORM

(attached)

ATTACHMENT X: 'INDICATION FOR MAP PHASE' FORM

(attached)

ANNEX 3, OP.02.01.FLS-RS.02. Pre-estudi 14/10/2014

ATTACHMENT Y: ONDANSETRON SUMMARY OF PRODUCT CHARACTERISTICS

(attached)

ATTACHMENT Z: SUBSTUDY BCN02-MICROBIOME PROTOCOL

(attached)

ATTACHMENT AA: SUBSTUDY BCN02-NEURO PROTOCOL

(attached)

ATTACHMENT AB: cART INTERRUPTION DECISION CHART

(attached)

ANNEX 3, OP.02.01.FLS-RS.02. Pre-estudi 14/10/2014
